# Investigating the shared genetic architecture between breast and ovarian cancers

**DOI:** 10.1590/1678-4685-GMB-2023-0181

**Published:** 2024-04-15

**Authors:** Xuezhong Shi, Anqi Bu, Yongli Yang, Yuping Wang, Chenyu Zhao, Jingwen Fan, Chaojun Yang, Xiaocan Jia

**Affiliations:** 1 Zhengzhou University, College of Public Health, Department of Epidemiology and Biostatistics, Zhengzhou, Henan, China.

**Keywords:** Genome-Wide Association Study, ovarian neoplasms, genetic pleiotropy, linkage disequilibrium, polymorphism

## Abstract

High heritability and strong correlation have been observed in breast and ovarian cancers. However, their shared genetic architecture remained unclear. Linkage disequilibrium score regression (LDSC) and heritability estimation from summary statistics (ρ-HESS) were applied to estimate heritability and genetic correlations. Bivariate causal mixture model (MiXeR) was used to qualify the polygenic overlap. Then, stratified-LDSC (S-LDSC) was used to identify tissue and cell type specificity. Meanwhile, the adaptive association test called MTaSPUsSet was performed to identify potential pleiotropic genes. The Single Nucleotide Polymorphisms (SNP) heritability was 13% for breast cancer and 5% for ovarian cancer. There was a significant genetic correlation between breast and ovarian cancers (r_g_=0.21). Breast and ovarian cancers exhibited polygenic overlap, sharing 0.4 K out 2.8 K of causal variants. Tissue and cell type specificity displayed significant enrichment in female breast mammary, uterus, kidney tissues, and adipose cell. Moreover, the 74 potential pleiotropic genes were identified between breast and ovarian cancers, which were related to the regulation of cell cycle and cell death. We quantified the shared genetic architecture between breast and ovarian cancers and shed light on the biological basis of the co-morbidity. Ultimately, these findings facilitated the understanding of disease etiology.

## Introduction

Breast and ovarian cancers were the major gynecologic malignancies with high morbidity and mortality (Smith et al., 2019). Breast cancer was the leading cause of cancer incidence worldwide in 2020, with an estimated 2.3 million new cases, representing 11.7% of all cancer cases in women (Sung et al., 2021). Ovarian cancer, one of major malignancies of the reproductive system, had the highest mortality rate among female cancers in North America, with 313,959 new cases and 207,252 deaths in 2020 (Sung *et al.*, 2021). And the incidences of breast cancer and ovarian cancer tended to be younger. They have become a major burden of cancer worldwide, greatly affecting women’s health and quality of life with high recurrences and low survival rates (Vergote et al., 2010; Valastyan and Weinberg, 2011). 

Although breast and ovarian cancers were clinically different types of malignant tumors, the number of patients with primary breast cancer combined with ovarian cancer was increasing. A Danish cohort study previously showed a significantly increased risk of breast cancer in women whose benign ovarian tumors were confined to solid ovarian tumors, suggesting some correlation between the two cancers (Gottschau et al., 2019). In addition, there were significant familial risks and the cumulative risks were higher in monozygotic than dizygotic twins, with heritability of 31% for breast cancer (Möller et al., 2016) and 22% for ovarian cancer (Mucci et al., 2016). Genome-wide association studies (GWAS) had identified more than 100 breast cancer susceptibility loci (Michailidou et al., 2013, 2017; Milne et al., 2017) and over 20 ovarian cancer susceptibility loci (Bojesen et al., 2013; Permuth-Wey *et al.*, 2013; Phelan et al., 2017). Meanwhile, there were over five loci associated with susceptibility of the both cancers (Merajver et al., 1995; Bojesen *et al.*, 2013). For example, carriers of *BRCA* (breast cancer susceptibility gene) genetic mutations had been found to be at high risk of breast and ovarian cancers (Merajver *et al.*, 1995). The pleiotropic genes had largely remained unknown due to limitations of traditional analytical methods. Moreover, univariate analysis could not comprehensively explore the genetic basis shared by the two cancers. Bivariate analysis could quantitatively estimate the shared genetic variants specific to cancers and improve our understanding of the polygenic structure and their relationships. Therefore, cross omics research could capitalize on this shared genetic architecture to identify pleiotropic variants in breast and ovarian cancers.

**Figure 1 -:**
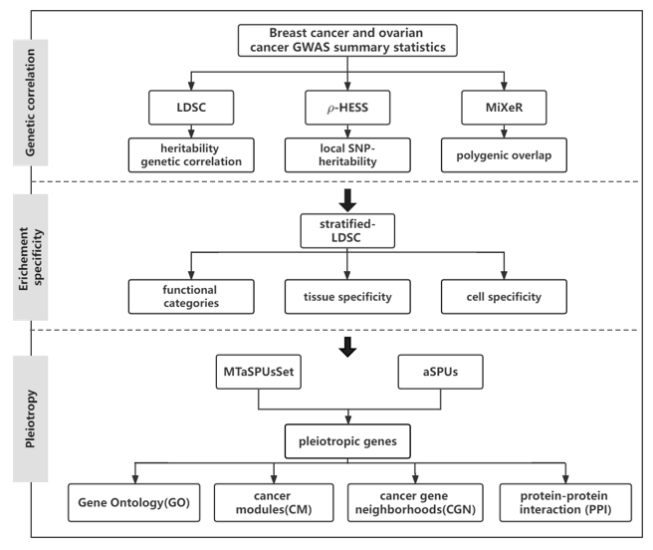
Flow chart of study design.

Here, we conducted an array of post-GWAS analyses to explore the genetic architecture of breast cancer and ovarian cancer including genetic correlations, tissue and cell type specificity, and pleiotropy. First, the MiXeR and ρ-HESS were applied to quantify the magnitude of genetic overlap and estimate the local genetic correlations, respectively. Then, the heritability proportion for specific functional categories was evaluated to identify tissue and cell type specificity by S-LDSC. Finally, the novel adaptive association analysis based on a class of sum of powered score (SPU) tests was applied to detect pleiotropic genes of breast and ovarian cancers (Kwak and Pan, 2017). A flowchart of our analysis strategy was provided in Figure 1.

## Material and Methods

### Study design and study samples

Summary statistics with breast cancer were obtained from a combined study including the Breast Cancer Association Consortium (BCAC, website: http://bcac.ccge.medschl.cam.ac.uk/), Discovery, Biology and Risk of Inherited Variants in Breast Cancer Consortium (DRIVE), Collaborative Oncological Gene-environment Study (iCOGS) and several other GWAS meta-analyses (Michailidou et al., 2017). It included 228,951 variates in 122,977 cases of breast cancer and 105,974 controls. Summary statistics with ovarian cancer were obtained from the Ovarian Cancer Association Consortium using an Illumina Custom Infinium array (OCAC, website: http://ocac.ccge.medschl.cam.ac.uk/). It included 66,450 variates in 25,509 cases and 40,941 controls (Phelan et al., 2017). The summary statistics were based on imputation to the 1,000 Genomes Project Phase 3 reference panel. The current results were for women of European ancestry only. The GWAS analysis for each disease was adjusted for principal components, including *P*, regression coefficients and standard error, using PLINK. More details about the cohorts and quality control (QC) process were explained in Michailidou *et al.* (2017) and Phelan *et al.* (2017). For the gene-level analysis, SNPs were removed with missing values or outliers, and end up with 10,723,398 SNPs for breast cancer and 18,169,480 SNPs for ovarian cancer left for analysis.

### LD score regression analysis

Linkage disequilibrium score regression provided heritability and confounding biases in SNPs based on summary statistics released from GWAS (Bulik-Sullivan et al., 2015; Zheng et al., 2017). Therefore, the SNP-based was apprised heritability due to genotyped and imputed SNPs (*h* ^*2*^_*SNP*_, i.e. the proportion of phenotypic variance in a trait can be explained by common genetic variants tagged on SNP arrays) of each cancer using LDSC (Python 2.7). And we used bivariate LDSC to evaluate genetic correlations (*r*_*g*_, i.e. the proportion of genetic variance shared by two traits divided by the square root of the product of their SNP heritability estimates) between breast and ovarian cancers. The 1,000 Genomes Project population of European ancestry was used as a reference group to ensure the quality of imputation (Auton et al., 2015).

### Assessing local SNP-heritability and genetic correlations

The ρ-HESS method (Shi et al., 2017) was used to evaluate local SNP-heritability and local genetic correlations from summary GWAS data (i.e. Z scores, effect sizes, and their SEs). This method divided the genome into 1,703 regions with an average size of nearly 1.5 MB (Berisa and Pickrell, 2016). Based on the 1,000 Genomes Europeans reference of hg19 genome build, the ρ-HESS could estimate the local genetic heritability per trait and genetic covariance between traits, then calculated the local genetic correlations from genetic heritability and covariance estimates.

### Quantification of polygenic overlap using MiXeR

Derived from causal mixture models applied to GWAS summary statistics, the MiXeR tool (https://github.com/precimed/mixer) was used to quantify the shared and unique polygenic components behind complex phenotypes. In cross-traits analysis, based on the assumption that only a small fraction of variants affected the trait, the bivariate MiXeR model additive genetic effects as a mixture of four components (Frei et al., 2019),

$${(\beta_{1j},\beta_{2j})\sim \pi_{0}N(0,0)+\pi_{1}N(0,\sum 1)+\pi_{2}N(0,\sum 2)+\pi_{12}N(0,\sum 12),}_{1}$$

$$\sum 1=\left[\begin{array}{cc} \sigma_{1}^{2} & 0\\ 0 & 0 \end{array}\right]\;,\;\;\;\;\;\;\;\sum 2=\left[\begin{array}{cc} 0 & 0\\ 0 & \sigma_{2}^{2} \end{array}\right],\;\;\;\;\;\;\;\;\sum 12=\left[\begin{array}{cc} \sigma_{1}^{2} & \rho_{12}\sigma_{1}\sigma_{2}\\ \rho_{12}\sigma_{1}\sigma_{2} & \sigma_{2}^{2} \end{array}\right]$$

where π_0_ was the proportion of null SNPs in the two traits. The π_1_ and π_2_ were proportions of SNPs with particular effects on the first and second trait, respectively, and π_12_ was the proportion of SNPs with an effect on both traits. In a variance-covariance ρ_12_ matrix, denoted the correlation of effect sizes in the shared component, and σ_1_^2^ and σ_2_^2^ indicated the variance of effect sizes of effective SNPs for the two traits. To estimate the number of effective variants, 1,000 genomes of Europeans was used as a reference panel. 

### Partitioning heritability

The method S-LDSC (Finucane et al., 2015) which partitioned heritability into different categories was used to calculate category-specific enrichments to identify the genomic function, tissue and cell type specificity. A ‘full baseline model’ was created from 24 publicly available master annotations that contained a total of 53 functional categories. To prevent our estimates from being biased upwards by enrichment in neighboring regions (Gusev et al., 2014), the full baseline model also included 500-bp windows around each functional category, as well as 100-bp windows around ChIP-seq peaks appropriately (Finucane et al., 2015). Based on the full baseline model, genomic functional specificity was performed. The tissue specificity analyses was used based on Genotype-Tissue Expression (GTEx) data that described variation in gene expression levels across 53 non-diseased human primary tissues (Battle et al., 2017). Then cell-type-specific analyses were conducted from the four histone marks H3K4me1, H3K4me3, H3K9ac, and H3K27ac. Each cell-type-specific annotation corresponded to a histone mark in a single cell type, with a total of 220 annotations (Finucane et al., 2015). The 220 cell-type-specific annotations were grouped into 10 new cell-type group annotations including adrenal/pancreas, central nervous system, cardiovascular, connective/bone, gastrointestinal, immune/hematopoietic, kidney, liver, skeletal muscle, and other (Finucane et al., 2015).

### MTaSPUsSet test for pleiotropic genes

There were d SNPs with additive genotype scores g = (g_1_,...g_*d*_), where g_*j*_ was the number of minor alleles of the j^th^ SNP; there were m > 1 quantitative or binary phenotypes Y = (Y_1_,...Y_*m*_)' let c = (c_1_,...c_*l*_)' denoted a set of covariates. This method considered a phenotype Y_*b*_ by applying a generalized linear model:

$$g\left[E(Y_{b})\right]=\beta_{b0}+\sum_{j=1}^{d}g_{j}\beta_{bj}+\alpha_{h}^{'}c$$

where g() was a canonical link function. This method was interested in testing H0:β_*bj*_ = 0 for all b = 1,...,m and j = 1,...,d. For a given dataset {(Y_*ib*_ ,g_*i*_ ,c_*i*_ ):i = 1,...,n} with n subjects, the score Z_*b*_ for β_*b*_ was

$$Z_{b}=\beta_{b}/se(\beta_{b})$$

Based on summary statistics, we conducted the multiple traits-single gene association analysis and single trait-single gene association analysis, respectively, and the calculation formula was as follows (Kwak & Pan, 2017)

$$SPUs\left(\gamma_{1};Z_{\left(b\right)}\right)={\left\|Z_{\left(b\right)}\right\|}_{\gamma_{1}}={\left(\sum_{j=1}^{d}Z_{bj}^{\gamma_{1}}\right)}^{1/\gamma_{1}}$$

$$MTSPUsSet\left(\gamma_{1},\gamma_{2};Z\right)=\sum_{b=1}^{m}{\left(SPUs\left(\gamma_{1};Z_{\left(b\right)}\right)\right)}^{\gamma_{2}}$$

where Z_*(b)*_ was the b^th^ row vector of the matrix Z such as the Z scores for the b^th^ traits (b ∈ {1,2}).

Finally, this method defined adjustive tests with γ_1_∈ Γ_1_= {1,2,4,8} and γ_2_∈ Γ_2_={1,2,4,8} to choose adaptively:

$$aSPUs(Z)=\underset{\gamma_{1}\in \Gamma_{1}}{min}p_{SPUs(\gamma_{1};Z)}$$

$$MTaSPUsSet(Z)=\underset{\gamma_{1}\in \Gamma_{1},\gamma_{2}\in \Gamma_{2}}{min}p_{MTSPUsSet\left(\gamma_{1},\gamma_{2};Z\right)}$$

where p_*SPUs(γ1;Z)*_ was the P value for aSPUs(γ_1_;Z). The p_*MTSPUsSet(γ1,γ2;Z)*_ was the P value for MTSPUsSet(γ_1_,γ_2_;Z). 

We explored marker-multiple cancers association using the Z-score for each SNP to narrow the differences between cancers. The SPU tests gave higher weight to larger Z-scores as their corresponding traits were more likely to be associated with the SNPs. The association between a single gene and a single trait was conducted by aSPUs tests. And the associations between a single gene and multiple traits were performed by MTaSPUsSet tests. To enable these tests, several data processing steps were conducted, including pruning SNPs and gene annotation. Firstly, a linkage disequilibrium (LD)-based SNP pruning method was employed to eliminate large pairwise correlated SNPs and keep a set of 140,722 SNPs. The HapMap 3 CEU genotypes were used as the reference panel. Secondly, based on the hg19 human dataset which was downloaded from the website: http://www.genome.ucsc.edu/cgi-bin/hgTables, gene annotation was performed for the pruned SNPs. A total of 67,657 common SNPs were located in 12,553 gene regions, which were eventually used to identify polymorphic variants.

### Functional annotation

The gene set analyses were implemented by using gene annotations of Gene Ontology (GO) functional categories, cancer gene neighborhoods (CGN) and cancer modules (CM) to investigate the biological insights of the pleiotropic genes. Moreover, protein-protein interaction (PPI) analysis was conducted to provide crucial protein functional associations of pleiotropic genes for visualization and molecular discovery (Szklarczyk et al., 2017) by using an available STRING dataset (website: ).

### Statistics analysis

The threshold was adjusted for nominal significance using the Bonferroni correction method (Ranstam, 2016). For the ρ-HESS method, the significance threshold of local SNP-heritability and local genetic correlations was set at P<0.05/1,703=2.9×10^-5^. And the significance threshold was set at P<0.05/53=9.4×10^-4^ for genomic function and tissue specificity. The significance thresholds were set at P<0.05/220=2.3×10^-4^ for cell-type specificity and at P<0.05/10=5×10^-3^ for cell-type-group specificity. For the MTaSPUsSet test and aSPUs test, P<3.75×10^-6^ (=0.05/12,553) was confirmed significant. All statistical analysis methods were preformed with LDSC software (version v1.0.1), the aSPU package of PLINK 1.9 and R 4.0.4. 

## Results

### Heritability estimates of breast cancer and ovarian cancer

LDSC analysis results showed that the liability-scale SNP heritability (without constrained intercept) was 13% for breast cancer and 5% for ovarian cancer. After removing genome-wide significant (P < 5×10^−8^) loci, 32% decrease in SNP-heritability for breast cancer and 13% decrease for ovarian cancer were observed, despite the fact that only 0.2% (breast cancer) to 0.03% (ovarian cancer) of the genome were excluded. And SNP-heritability were partitioned into 53 genomic functional annotations. The Conserved_LindbladToh showed a significant enrichment for breast cancer. The enrichment results of genomic functional annotations for breast and ovarian cancers were presented in Table S1.

### Genetic correlations between breast cancer and ovarian cancer

The genome-wide genetic correlation genetic correlations analysis showed that there was a statistically significant between breast cancer and ovarian cancer (r_*g*_ =0.21, se=0.06). A total of 37 statistically significant regions were identified for breast cancer and 1 statistically significant region was identified for ovarian cancer, with a significance threshold of P < 0.05/1,703=2.9×10^-5^ (Table S2). For local genetic correlations the ρ-HESS showed that no significant local genetic correlations was identified between breast cancer and ovarian cancer.

### Genetic overlap between breast cancer and ovarian cancer

**Figure 2 -:**
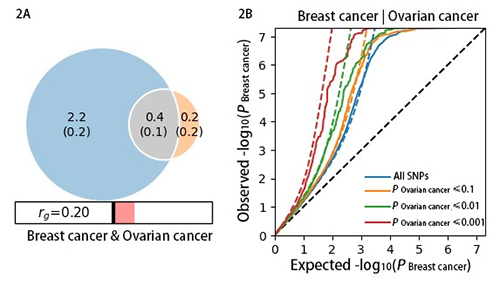
Venn diagrams of unique and shared polygenic variants between cancers. 2A: The gray presented polygenic overlap between two cancers, the blue represented unique variants of breast cancer, and the orange represented unique variants of ovarian cancer. The numbers indicated the estimated quantity of effective variants (in thousands) per component, explain 90% of SNP heritability in each cancer, followed by the standard error. The size of the circle reflected the degree of polygenicity. 2B: Conditional Q-Q plots of observed versus expected -log10(P) values in the primary trait as a function of significance of association with a secondary trait at the level of P < 0.1, P < 0.01, P < 0.001, respectively.

In the conditional Q-Q plot, each line displayed a leftward separation, indicating a polygenic overlap between breast cancer and ovarian cancer. The results of the Venn diagram, which represented the polygenic components showed that the breast cancer and ovarian cancer exhibited polygenic overlap, sharing 0.4 K out 2.8 K of causal variants (Figure 2). In addition, the genetic correlation estimated by MiXeR was generally consistent with the result by LDSC. The polygenic overlap was quantifying by this method, which could supplement genetic correlation analysis and improve our understanding of cross-trait genetic architectures.

### Tissue and cell type specificity for breast cancer and ovarian cancer

**Figure 3 -:**
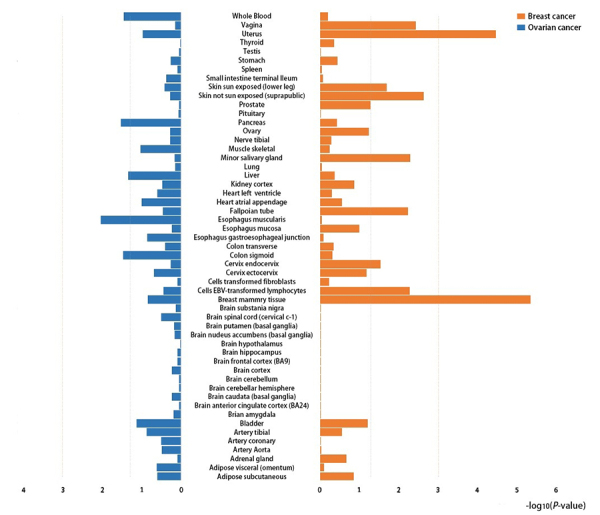
Tissue type-specific enrichment of SNP heritability for cancers. The x-axis represented each of the 53 tissue types, y-axis represented the log-transformed P-values of coefficient Z scores. The horizontal grey dash line indicated P-threshold of 0.05; horizontal red dash line indicated P-threshold of 0.05/53.

For tissue specificity, significant heritability enrichment was observed in breast mammary and uterus tissues in breast cancer, with a significance threshold of P<0.05/53=9.4×10^-4^. And there was no significant heritability enrichment for ovarian cancer (Figure 3, Table S3). The results of heritability enrichment shown that five systems were significantly enriched in breast cancer including gastrointestinal, cardiovascular, kidney, connective bone and other systems, with a significance threshold of P<0.05/10=P<5×10^-3^. But there was no system significantly enriched in ovarian cancer. Additionally, compared with other tissues, ovarian cancer also exhibited substantial enrichment in kidney tissue, although yielding only nominal significance (Table S4, Figure S1). There were 3 significant cell type enrichments for breast cancer and no significant cell type enrichments for ovarian cancer, with a significance threshold of P<0.05/220= 2.3×10^-4^ (Table S5, Figure S2).

### Pleiotropic genes between breast cancer and ovarian cancer

**Figure 4 -:**
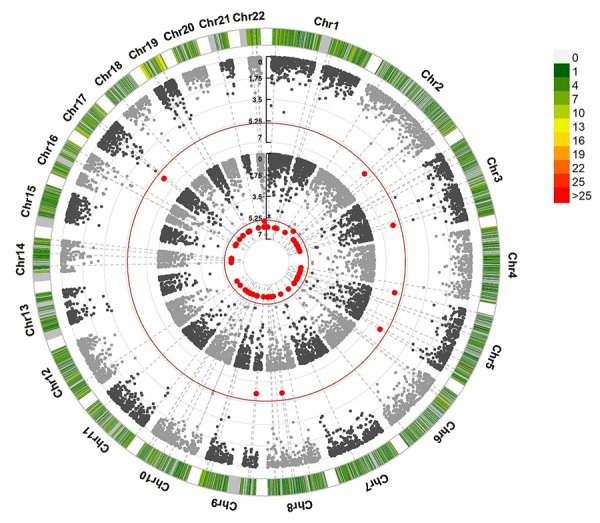
Manhattan plot with -log10(P) values of aSPUs test. The black line is a threshold with the -log10(P) value of 5.40 corresponding to P < 3.75×10^-6^. If the -log10(P) value of a certain gene was >5.40, this gene was identified as significant for the trait. From the inside out, the first ring is the aSPUs test for gene with breast cancer association, the second ring is gene with ovarian cancer.

In total, 140,721 SNPs were mapped to 12,553 genes. The MTaSPUsSet tests showed a total of 81 genes were associated with breast cancer and ovarian cancer (Bonferroni correction P<3.75×10^-6^) (Figure S3). The aSPUs tests showed there were 78 genes associated with breast cancer and 8 genes associated with ovarian cancer (Bonferroni correction P<3.75×10^-6^) (Figure 4). By aggregating the results of these two tests, the pleiotropic genes those were statistically significant were defined by the MTaSPUsSet test and statistically significant by the aSPUs test for at least one cancer. Eventually, 74 potential pleiotropic genes were found (Table S6). Notably, the gene TERT was significantly associated with both breast cancer and ovarian cancer detected by the two tests.

### Functional annotations of pleiotropic genes

**Figure 5 -:**
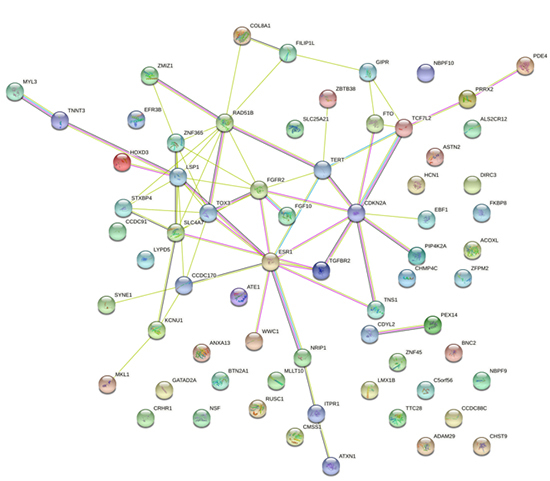
The protein-protein interactions across pleiotropic genes for breast cancer and ovarian cancer. The network nodes are proteins. The edges represent the predicted functional associations. Red line indicates the presence of fusion evidence; Green line indicates neighborhood evidence; Blue line indicates cooccurrence evidence; Purple line indicates experimental evidence; Yellow line indicates text-mining evidence; Light blue line indicates database evidence; Black line indicates co-expression evidence.

To explore the biological pathways of pleiotropic gene enrichment, gene-set analyses were conducted. We identified 23 significant GO functional terms focusing on the regulation of cell cycle, cell death and female sex differentiation. In addtion, 19 cancer gene neighborhoods and one cancer module were found for both cancers. The information on significant pathways was shown in Table S7. Considering that the activity and function of proteins were usually modulated by other interacting proteins, the PPI analysis was applied to visualize the interaction of pleiotropic genes. And we observed two major different gene clusters containing LSP1 cluster and ESR1 cluster, which were related to the regulation of cell cycle, cell death and female sex differentiation (Figure 5).

## Discussion

In the present study, a comprehensive analysis was performed to explore the shared genetic architecture and pleiotropy of breast and ovarian cancers. It verified the strong genetic correlation between the two cancers and the heritability was enriched in breast mammary, uterus, kidney and adipose cells. Based on integration of the resultes of aSPUs and MTaSPUsSet tests, 74 potential pleiotropic genes which were related to the regulation of cell cycle, cell death and female sex differentiation were detected. These findings provided methodological insights into the analysis of the shared genetic structure of diseases with similar genetic factors for reference, thus potentially provided intervention targets.

The occurrence of breast cancer and ovarian cancer was known to be influenced by genetic factors (Hulka, 1997), consistent with the heritability analysis in our study. Most of the genetic effects were attributed to other undiscovered variants, although genome-wide significant variants accounted for a portion of the heritability of breast cancer and ovarian cancer. The significant heritability enrichment of conserved regions revealed the biological importance for breast cancer, consistent with the fact that ultra-conserved regions of transcription tended to be located at vulnerable sites (Finucane et al., 2015). A significant genetic correlation (r_*g*_ =0.21) and polygenic overlap between breast and ovarian cancer was observed, which were broadly in line with previous estimates (Jiang et al., 2019; Si et al., 2021). These evidences suggested that these cancers could not be regarded as completely independent diseases (Amundadottir et al., 2004; Frank et al., 2017) and genetic factors made a strong contribution to the comorbidity of breast cancer and ovarian cancer. Tissue type specific analysis revealed substantial heritable enrichment of breast, uterus and kidney tissue in women with breast cancer and ovarian cancer. It revealed that alterations in these regions of women were responsible for triggering cancers. This result was consistent with the fact that these regions affected the levels of sex hormones (Miller, 2009; Gibson et al., 2020) and thus played roles in the growth of breast and ovarian cancer (Key et al., 2013; Brown and Hankinson, 2015). Cell type specific analysis revealed the prominent role of adipose cells, which was associated with the rich adipose tissue in the female breast. The breast adipose tissue played a major role in the communication of all components of the breast microenvironment. The interaction between breast adipose tissue surrounding cancer cells and vice-versa modified the tumor microenvironment in favor of cancer development (Kothari et al., 2020). 

The 74 potential pleiotropic genes were detected to provide further support for the shared genetic architecture of breast and ovarian cancers. And these pleiotropic genes were found to be associated with the regulation of cell cycle, cell death and female sex differentiation by gene-set analyses. There were some univariate studies of breast cancer and ovarian cancer being reported, respectively. However, no studies had quantified common genetic variation between these two cancers. Additionally, comparing the results from the previous univariate studies, it suggested that 54 genes have been reported to be associated with breast cancer or ovarian cancer. Our study not only validated those previous univariate studies, but also identified some new pleiotropic genes through adaptive association analysis. For example, overexpression of CCDC170 in breast cancer cells increased the protein levels of IRE1α which was an important determinant of cell death and survival in previous study (Maurel et al., 2014). It was consistent with the results of functional annotation by gene set analyses, where the gene was found to be involved in the regulation of cell death, female sex differentiation and cancer. Moreover, a previous study demonstrated that CCDC170 was fused to ESR1 in breast cancer (Veeraraghavan et al., 2014). The fused gene was found to promote a more aggressive phenotype. At the same time, we found that there were functional interactions between the expressed protein of CCDC170 and the expressed protein of ESR1, which was a validation of the previous study. Additionally, the TERT locus was previously reported to be associated with breast cancer and ovarian cancer (Bojesen et al., 2013). Research in the past few decades had revealed that the telomerase holoenzyme was tightly regulated by repressing its rate-limiting component, telomerase reverse transcriptase (TERT) (Saretzki, 2014). And cells lacking telomerase holoenzyme showed increased radiation sensitivity and reduced DNA repair capacity (Masutomi et al., 2005), which corresponded to our understanding of the TERT gene involved in regulation of cell cycle, cell death and sequence specific DNA binding. In addition to the few pleiotropic genes already identified, the present study inspected 20 novel genes by adaptive association tests. For instance, our data demonstrated the significance of the CRHR1 gene in the GO pathway for negative regulation of cell death and in the cancer gene neighborhoods pathway based on the gene-set analyses. Prior studies had revealed that activation of CRH receptors reduced vascular endothelial growth factor synthesis and cell proliferation in different tumor entities (Bale et al., 2002; Hao et al., 2008). A previous prospective study found the contribution of genetic variants in hypothalamic-pituitary-adrenal (HPA) axis genes including CRHR1 to the risk of developing breast cancer (Nan et al., 2015), which was consistent with our result. Moreover, our study provided new evidence to support previous studies on the role of CRHR1 in tumorigenesis progression of breast and ovarian cancer. Expression of the annexin family had been studied in a wide range of cancers, including ANXA1, ANXA2 and ANXA13 (Mussunoor and Murray, 2008) Annexin family members were involved in signal transduction, cellular differentiation, proliferation and thus in tumorigenesis (Lizarbe et al., 2013). Our study also suggested that ANXA13 played a role in tumorigenesis. The novel gene, FKBP8, was an intrinsic inhibitor of mTOR kinase that exerted an anti-apoptotic function (Bai et al., 2007). As one of the most frequently modified signaling pathways, the PI3K-Akt-mTOR axis activation maintained cancer growth (LoRusso, 2016). These findings provided novel evidence supporting shared etiology and pathogenesis for breast cancer and ovarian cancer. In summary, the underlyig pleiotropic genes may influence the regulation of the cell cycle, cell death and female sex differentiation, and thus play a role in cancer development through telomerase, protein and other pathways. These findings shed light on the underlying genetic mechanisms that on the common etiology and pathogenesis of breast and ovarian cancers.

Our study has several strengths. We quantified the genetic correlations by leveraging large GWAS summary statistics. Furthermore, compared to univariate statistical analysis, our bivariate analysis was more powerful and adaptive by aggregating the multiple association signals and reducing the burden of multiple testing. It is worth noting that the following limitations should be taken into account when interpreting results using the MTaSPUsSet tests. Firstly, due to the lack of biological information at the individual level, we were unable to determine whether the pleiotropic genes had a direct or indirect effect on cancer risk. Secondly, the biological mechanisma underlying breast and ovarian cancers remained understood. Therefore, further experimental studies based on our findings are needed.

## Conclusion

Our study revealed strong genetic correlations and 74 common pleiotropic genes across breast and ovarian cancers. These findings provided important clues to explore the common molecular mechanisms and biological processes underlying breast and ovarian cancers, as well as a novel statistical analysis strategy for studying complex diseases.

## Supplementary material

The following online material is available for this article:

Table S1 - Summary of SNP enrichment estimates in functional category annotations for breast cancer and ovarian cancer.

Table S2 - The regions for each cancer that reach the 2.93×e^-5^ threshold (P-values).

Table S3 - Summary of SNP enrichment in tissue types for breast cancer and ovarian cancer.

Table S4 - Summary of SNP enrichment in cell type groups for breast cancer and ovarian cancer.

Table S5 - Summary of SNP enrichment in cell types for breast cancer and ovarian cancer.

Table S6 - The genes identified by the MTaSPUsSet and aSPUs test.

Table S7 - The significant pathway enrichment of the pleiotropic genes.

Figure S1 - Cell-type-group-specific enrichment of SNP heritability for cancers.

Figure S2 - Cell-type-specific enrichment of SNP heritability for cancers.

Figure S3 - Manhattan plot with -log10(P) values of MTaSPUsSet test for single gene with two cancers analysis.
