# Erratum: Investigating the shared genetic architecture between breast and ovarian cancers

**DOI:** 10.1590/1678-4685-GMB-2023-0181er

**Published:** 2024-08-16

In the article “Investigating the shared genetic architecture between breast and ovarian cancers”, with DOI code number https://doi.org/10.1590/1678-4685-GMB-2023-0181, published in the Genetics and Molecular Biology, 47(2):e20230181 (2024):

**Where it was written:**

**Acknowledgements**

The authors expressed gratitude to Breast Cancer Association Consortium, Ovarian Cancer Association Consortium, Genotype-Tissue Expression for providing data and national supercomputing center in Zhengzhou for data platform.

**Should read:**

**Acknowledgements**

The authors expressed gratitude to Breast Cancer Association Consortium, Ovarian Cancer Association Consortium, Genotype-Tissue Expression for providing data and national supercomputing center in Zhengzhou for data platform.

This research was supported by the National Natural Science Foundation of China (No.82073670) and Key Scientific Research Program of Higher Education Institutions of Henan Province (23A330003).

