# Supplementary material for: Investigating the shared genetic architecture between breast and ovarian cancers
Source: Genet Mol Biol. 2024 Apr 15;47(2):e20230181. doi: 10.1590/1678-4685-GMB-2023-0181 (PMC11021043; doi:10.1590/1678-4685-GMB-2023-0181)
Supplement: Table S1 - [file 1415-4757-GMB-47-02-e20230181-s1.pdf]

Genetics and Molecular Biology

https://doi.org/10.1590/1678-4685-GMB-2023-0181

Supplementary Material to "Investigating the shared genetic architecture between breast and ovarian cancers"

Table S1 - Summary of SNP enrichment estimates in functional category annotations for breast cancer and ovarian cancer.

**Phenotypes**	**Category**	**Proportion of SNPs**	**Proportion of h^2**	**Proportion of h^2 SE**	**Enrichment**	**Enrichment SE**	**Enrichment *P*-value**	**Coefficient**	**Coefficient SE**	**Coefficient *Z*-value**	**Coefficient *P*-value**
Brest cancer	base	1.00E+00	1.00E+00	0.00E+00	1.00E+00	0.00E+00	NA	8.58E-10	8.62E-09	9.96E-02	4.60E-01
Brest cancer	Coding_UCSC.bed	1.43E-02	5.05E-02	2.84E-02	3.54E+00	1.99E+00	2.04E-01	-3.31E-08	4.14E-08	-7.99E-01	7.88E-01
Brest cancer	Coding_UCSC.extend.500.bed	6.37E-02	1.41E-01	3.02E-02	2.22E+00	4.74E-01	1.04E-02	-9.03E-10	1.52E-08	-5.93E-02	5.24E-01
**Brest cancer**	**Conserved_LindbladToh.bed**	**2.57E-02**	**2.64E-01**	**5.15E-02**	**1.03E+01**	**2.01E+00**	**7.42E-06**	**1.37E-07**	**3.56E-08**	**3.85E+00**	**5.81E-05**
Brest cancer	Conserved_LindbladToh.extend.500.bed	3.30E-01	6.64E-01	7.45E-02	2.01E+00	2.26E-01	1.67E-05	3.80E-09	7.43E-09	5.12E-01	3.04E-01
Brest cancer	CTCF_Hoffman.bed	2.39E-02	-1.90E-02	3.77E-02	-7.97E-01	1.58E+00	2.53E-01	-4.82E-08	3.51E-08	-1.38E+00	9.16E-01
Brest cancer	CTCF_Hoffman.extend.500.bed	7.08E-02	7.68E-03	4.51E-02	1.08E-01	6.36E-01	1.59E-01	-2.32E-08	1.60E-08	-1.45E+00	9.26E-01
Brest cancer	DGF_ENCODE.bed	1.36E-01	4.04E-01	9.57E-02	2.97E+00	7.04E-01	6.70E-03	-1.58E-09	1.75E-08	-9.03E-02	5.36E-01
Brest cancer	DGF_ENCODE.extend.500.bed	5.38E-01	1.04E+00	6.12E-02	1.93E+00	1.14E-01	9.55E-14	1.78E-08	7.38E-09	2.41E+00	7.87E-03

Phenotypes	Category	Proportion of SNPs	Proportion of h^2	Proportion of h^2 SE	Enrichment	Enrichment SE	Enrichment *P*-value	Coefficient	Coefficient SE	Coefficient *Z*-value	Coefficient *P*-value
Brest cancer	DHS_peaks_Trynka.bed	1.11E-01	3.42E-01	8.55E-02	3.10E+00	7.73E-01	8.65E-03	-1.87E-08	2.61E-08	-7.19E-01	7.64E-01
Brest cancer	DHS_Trynka.bed	1.66E-01	5.31E-01	1.09E-01	3.20E+00	6.56E-01	1.14E-03	1.01E-08	2.63E-08	3.85E-01	3.50E-01
Brest cancer	DHS_Trynka.extend.500.bed	4.96E-01	8.34E-01	7.06E-02	1.68E+00	1.42E-01	4.11E-06	-8.66E-09	9.85E-09	-8.79E-01	8.10E-01
Brest cancer	Enhancer_Andersson.bed	4.33E-03	3.83E-02	1.97E-02	8.86E+00	4.55E+00	8.66E-02	4.64E-08	8.69E-08	5.33E-01	2.97E-01
Brest cancer	Enhancer_Andersson.extend.500.bed	1.90E-02	1.02E-01	2.68E-02	5.37E+00	1.41E+00	2.01E-03	2.57E-08	3.10E-08	8.31E-01	2.03E-01
Brest cancer	Enhancer_Hoffman.bed	4.20E-02	1.38E-01	4.59E-02	3.29E+00	1.09E+00	3.80E-02	-2.44E-08	3.08E-08	-7.93E-01	7.86E-01
Brest cancer	Enhancer_Hoffman.extend.500.bed	8.98E-02	2.70E-01	4.64E-02	3.00E+00	5.17E-01	1.24E-04	6.30E-09	2.09E-08	3.01E-01	3.82E-01
Brest cancer	FetalDHS_Trynka.bed	8.39E-02	3.42E-01	8.17E-02	4.07E+00	9.74E-01	2.05E-03	1.03E-08	2.77E-08	3.73E-01	3.55E-01
Brest cancer	FetalDHS_Trynka.extend.500.bed	2.83E-01	6.19E-01	7.11E-02	2.18E+00	2.51E-01	6.79E-06	-5.17E-10	9.81E-09	-5.27E-02	5.21E-01
Brest cancer	H3K27ac_Hnisz.bed	3.89E-01	7.67E-01	3.93E-02	1.97E+00	1.01E-01	8.39E-18	-2.85E-08	2.27E-08	-1.26E+00	8.95E-01
Brest cancer	H3K27ac_Hnisz.extend.500.bed	4.20E-01	8.49E-01	4.21E-02	2.02E+00	1.00E-01	7.38E-20	3.22E-08	2.26E-08	1.42E+00	7.76E-02
Brest cancer	H3K27ac_PGC2.bed	2.69E-01	5.94E-01	6.75E-02	2.21E+00	2.51E-01	3.73E-06	-3.34E-08	1.91E-08	-1.75E+00	9.60E-01
Brest cancer	H3K27ac_PGC2.extend.500.bed	3.35E-01	7.78E-01	4.70E-02	2.32E+00	1.40E-01	1.51E-16	3.05E-08	1.62E-08	1.88E+00	2.98E-02
Brest cancer	H3K4me1_peaks_Trynka.bed	1.70E-01	4.97E-01	9.37E-02	2.93E+00	5.52E-01	6.76E-04	1.52E-09	1.54E-08	9.86E-02	4.61E-01
Brest cancer	H3K4me1_Trynka.bed	4.24E-01	9.41E-01	6.40E-02	2.22E+00	1.51E-01	1.86E-13	1.77E-08	1.08E-08	1.65E+00	4.97E-02
Brest cancer	H3K4me1_Trynka.extend.500.bed	6.06E-01	9.11E-01	4.74E-02	1.50E+00	7.82E-02	3.11E-09	-1.41E-08	8.74E-09	-1.62E+00	9.47E-01
Brest cancer	H3K4me3_peaks_Trynka.bed	4.16E-02	2.72E-01	6.23E-02	6.54E+00	1.50E+00	2.08E-04	5.34E-08	3.30E-08	1.62E+00	5.29E-02
Brest cancer	H3K4me3_Trynka.bed	1.33E-01	4.69E-01	6.31E-02	3.53E+00	4.74E-01	3.68E-07	8.85E-10	2.00E-08	4.42E-02	4.82E-01
Brest cancer	H3K4me3_Trynka.extend.500.bed	2.55E-01	5.80E-01	5.67E-02	2.27E+00	2.22E-01	8.65E-08	-7.28E-09	1.16E-08	-6.26E-01	7.34E-01
Brest cancer	H3K9ac_peaks_Trynka.bed	3.85E-02	1.72E-01	6.14E-02	4.46E+00	1.60E+00	3.17E-02	-1.36E-08	3.35E-08	-4.06E-01	6.58E-01
Brest cancer	H3K9ac_Trynka.bed	1.25E-01	5.02E-01	6.87E-02	4.00E+00	5.48E-01	1.17E-07	3.08E-08	2.01E-08	1.53E+00	6.28E-02

**Phenotypes**	**Category**	**Proportion of SNPs**	**Proportion of h^2**	**Proportion of h^2 SE**	**Enrichment**	**Enrichment SE**	**Enrichment *P*-value**	**Coefficient**	**Coefficient SE**	**Coefficient *Z*-value**	**Coefficient *P*-value**
Brest cancer	H3K9ac_Trynka.extend.500.bed	2.30E-01	5.88E-01	5.90E-02	2.56E+00	2.57E-01	1.35E-08	-5.51E-09	1.40E-08	-3.94E-01	6.53E-01
Brest cancer	Intron_UCSC.bed	3.87E-01	4.48E-01	3.18E-02	1.16E+00	8.21E-02	5.80E-02	2.32E-09	6.33E-08	3.67E-02	4.85E-01
Brest cancer	Intron_UCSC.extend.500.bed	3.97E-01	4.99E-01	2.44E-02	1.26E+00	6.16E-02	4.04E-05	-5.22E-09	6.34E-08	-8.23E-02	5.33E-01
Brest cancer	Promoter_UCSC.bed	3.07E-02	7.73E-02	2.46E-02	2.52E+00	8.03E-01	5.91E-02	-3.45E-08	5.93E-08	-5.82E-01	7.20E-01
Brest cancer	Promoter_UCSC.extend.500.bed	3.81E-02	1.04E-01	2.19E-02	2.74E+00	5.75E-01	2.45E-03	2.98E-08	5.21E-08	5.72E-01	2.84E-01
Brest cancer	PromoterFlanking_Hoffman.bed	8.31E-03	5.90E-04	2.29E-02	7.10E-02	2.75E+00	7.36E-01	-4.59E-08	5.97E-08	-7.68E-01	7.79E-01
Brest cancer	PromoterFlanking_Hoffman.extend.500.bed	3.32E-02	6.48E-02	3.23E-02	1.95E+00	9.73E-01	3.31E-01	-9.39E-09	2.66E-08	-3.54E-01	6.38E-01
Brest cancer	Repressed_Hoffman.bed	4.61E-01	1.29E-01	7.24E-02	2.81E-01	1.57E-01	1.07E-05	-9.92E-09	8.92E-09	-1.11E+00	8.67E-01
Brest cancer	Repressed_Hoffman.extend.500.bed	7.19E-01	4.53E-01	3.52E-02	6.30E-01	4.91E-02	2.87E-13	8.15E-09	6.73E-09	1.21E+00	1.13E-01
Brest cancer	SuperEnhancer_Hnisz.bed	1.67E-01	4.18E-01	2.31E-02	2.50E+00	1.38E-01	1.39E-21	3.95E-09	7.82E-08	5.05E-02	4.80E-01
Brest cancer	SuperEnhancer_Hnisz.extend.500.bed	1.70E-01	4.24E-01	2.33E-02	2.49E+00	1.37E-01	7.08E-22	1.70E-09	7.71E-08	2.21E-02	4.91E-01
Brest cancer	TFBS_ENCODE.bed	1.31E-01	5.74E-01	9.61E-02	4.37E+00	7.32E-01	1.02E-05	3.59E-08	2.06E-08	1.74E+00	4.06E-02
Brest cancer	TFBS_ENCODE.extend.500.bed	3.41E-01	8.39E-01	7.00E-02	2.46E+00	2.05E-01	3.87E-11	1.31E-08	9.68E-09	1.35E+00	8.88E-02
Brest cancer	Transcribed_Hoffman.bed	3.46E-01	4.51E-01	6.37E-02	1.30E+00	1.84E-01	9.91E-02	5.00E-09	8.23E-09	6.07E-01	2.72E-01
Brest cancer	Transcribed_Hoffman.extend.500.bed	7.62E-01	6.15E-01	5.58E-02	8.07E-01	7.32E-02	9.70E-03	-1.02E-08	6.43E-09	-1.59E+00	9.44E-01
Brest cancer	TSS_Hoffman.bed	1.78E-02	1.51E-01	3.20E-02	8.49E+00	1.80E+00	4.63E-05	8.33E-08	4.79E-08	1.74E+00	4.08E-02
Brest cancer	TSS_Hoffman.extend.500.bed	3.44E-02	1.51E-01	3.34E-02	4.40E+00	9.72E-01	5.13E-04	-3.97E-08	3.21E-08	-1.24E+00	8.92E-01
Brest cancer	UTR_3_UCSC.bed	1.12E-02	1.19E-02	1.74E-02	1.07E+00	1.56E+00	9.66E-01	-1.65E-08	4.53E-08	-3.65E-01	6.42E-01
Brest cancer	UTR_3_UCSC.extend.500.bed	2.65E-02	4.94E-02	2.26E-02	1.87E+00	8.53E-01	3.11E-01	7.34E-09	2.54E-08	2.89E-01	3.86E-01
Brest cancer	UTR_5_UCSC.bed	5.48E-03	4.86E-02	1.79E-02	8.86E+00	3.26E+00	1.69E-02	1.04E-07	7.16E-08	1.45E+00	7.29E-02
Brest cancer	UTR_5_UCSC.extend.500.bed	2.69E-02	8.69E-02	2.44E-02	3.23E+00	9.08E-01	1.43E-02	-2.24E-08	2.34E-08	-9.55E-01	8.30E-01

Phenotypes	Category	Proportion of SNPs	Proportion of h^2	Proportion of h^2 SE	Enrichment	Enrichment SE	Enrichment *P*-value	Coefficient	Coefficient SE	Coefficient *Z*-value	Coefficient *P*-value
Brest cancer	WeakEnhancer_Hoffman.bed	2.10E-02	8.34E-02	3.82E-02	3.97E+00	1.82E+00	1.04E-01	1.42E-08	3.68E-08	3.86E-01	3.50E-01
Brest cancer	WeakEnhancer_Hoffman.extend.500.bed	8.88E-02	1.83E-01	4.68E-02	2.07E+00	5.27E-01	4.55E-02	-1.61E-08	1.44E-08	-1.12E+00	8.69E-01
Ovarian cancer	base	1.00E+00	1.00E+00	1.13E-07	1.00E+00	1.13E-07	NA	-1.64E-08	8.71E-09	-1.88E+00	9.70E-01
Ovarian cancer	Coding_UCSC.bed	1.43E-02	1.59E-02	1.14E-01	1.12E+00	7.99E+00	9.88E-01	-1.56E-07	9.48E-08	-1.64E+00	9.50E-01
Ovarian cancer	Coding_UCSC.extend.500.bed	6.37E-02	2.52E-01	1.17E-01	3.96E+00	1.84E+00	1.09E-01	-9.49E-09	2.69E-08	-3.52E-01	6.38E-01
Ovarian cancer	Conserved_LindbladToh.bed	2.57E-02	5.03E-01	2.38E-01	1.96E+01	9.28E+00	6.67E-02	1.74E-07	8.95E-08	1.94E+00	2.60E-02
Ovarian cancer	Conserved_LindbladToh.extend.500.bed	3.30E-01	7.95E-01	2.17E-01	2.41E+00	6.55E-01	3.61E-02	6.54E-10	1.11E-08	5.89E-02	4.77E-01
Ovarian cancer	CTCF_Hoffman.bed	2.39E-02	-2.29E-01	2.05E-01	-9.61E+00	8.58E+00	1.79E-01	-8.42E-08	8.77E-08	-9.60E-01	8.32E-01
Ovarian cancer	CTCF_Hoffman.extend.500.bed	7.08E-02	-2.74E-01	2.28E-01	-3.87E+00	3.22E+00	9.67E-02	-3.58E-08	3.95E-08	-9.06E-01	8.17E-01
Ovarian cancer	DGF_ENCODE.bed	1.36E-01	5.72E-02	4.51E-01	4.20E-01	3.31E+00	8.60E-01	-5.55E-08	4.08E-08	-1.36E+00	9.13E-01
Ovarian cancer	DGF_ENCODE.extend.500.bed	5.38E-01	1.32E+00	2.51E-01	2.45E+00	4.66E-01	4.82E-03	2.49E-08	1.53E-08	1.63E+00	5.18E-02
Ovarian cancer	DHS_peaks_Trynka.bed	1.11E-01	6.72E-01	4.14E-01	6.08E+00	3.75E+00	1.77E-01	1.28E-07	6.17E-08	2.07E+00	1.91E-02
Ovarian cancer	DHS_Trynka.bed	1.66E-01	3.40E-01	4.02E-01	2.04E+00	2.42E+00	6.68E-01	-3.22E-08	4.81E-08	-6.70E-01	7.48E-01
Ovarian cancer	DHS_Trynka.extend.500.bed	4.96E-01	1.14E+00	2.76E-01	2.30E+00	5.56E-01	2.72E-02	9.68E-10	2.03E-08	4.78E-02	4.81E-01
Ovarian cancer	Enhancer_Andersson.bed	4.33E-03	2.53E-02	8.89E-02	5.85E+00	2.05E+01	8.12E-01	2.81E-08	2.02E-07	1.39E-01	4.45E-01
Ovarian cancer	Enhancer_Andersson.extend.500.bed	1.90E-02	6.99E-02	1.10E-01	3.67E+00	5.80E+00	6.44E-01	2.58E-09	6.54E-08	3.95E-02	4.84E-01
Ovarian cancer	Enhancer_Hoffman.bed	4.20E-02	9.18E-02	1.76E-01	2.19E+00	4.19E+00	7.78E-01	-6.40E-08	6.89E-08	-9.28E-01	8.23E-01
Ovarian cancer	Enhancer_Hoffman.extend.500.bed	8.98E-02	6.10E-01	1.83E-01	6.79E+00	2.04E+00	6.07E-03	6.05E-08	4.43E-08	1.37E+00	8.61E-02
Ovarian cancer	FetalDHS_Trynka.bed	8.39E-02	-1.47E-01	3.54E-01	-1.75E+00	4.22E+00	4.85E-01	-1.07E-07	5.64E-08	-1.89E+00	9.71E-01
Ovarian cancer	FetalDHS_Trynka.extend.500.bed	2.83E-01	6.63E-01	2.78E-01	2.34E+00	9.80E-01	1.96E-01	1.07E-08	2.20E-08	4.87E-01	3.13E-01
Ovarian cancer	H3K27ac_Hnisz.bed	3.89E-01	7.40E-01	1.45E-01	1.90E+00	3.72E-01	2.04E-02	-5.19E-09	4.69E-08	-1.11E-01	5.44E-01

**Phenotypes**	**Category**	**Proportion of SNPs**	**Proportion of h^2**	**Proportion of h^2 SE**	**Enrichment**	**Enrichment SE**	**Enrichment *P*-value**	**Coefficient**	**Coefficient SE**	**Coefficient *Z*-value**	**Coefficient *P*-value**
Ovarian cancer	H3K27ac_Hnisz.extend.500.bed	4.20E-01	8.34E-01	1.71E-01	1.98E+00	4.08E-01	2.27E-02	-1.61E-09	4.71E-08	-3.42E-02	5.14E-01
Ovarian cancer	H3K27ac_PGC2.bed	2.69E-01	1.05E+00	2.75E-01	3.90E+00	1.02E+00	3.67E-03	4.58E-08	3.82E-08	1.20E+00	1.15E-01
Ovarian cancer	H3K27ac_PGC2.extend.500.bed	3.35E-01	8.24E-01	1.91E-01	2.46E+00	5.69E-01	1.80E-02	-3.79E-08	3.38E-08	-1.12E+00	8.69E-01
Ovarian cancer	H3K4me1_peaks_Trynka.bed	1.70E-01	3.82E-01	3.65E-01	2.25E+00	2.15E+00	5.65E-01	2.14E-09	2.91E-08	7.34E-02	4.71E-01
Ovarian cancer	H3K4me1_Trynka.bed	4.24E-01	9.00E-01	2.79E-01	2.12E+00	6.59E-01	1.04E-01	-8.07E-10	2.29E-08	-3.51E-02	5.14E-01
Ovarian cancer	H3K4me1_Trynka.extend.500.bed	6.06E-01	8.28E-01	1.62E-01	1.37E+00	2.68E-01	1.85E-01	-2.12E-08	1.75E-08	-1.21E+00	8.87E-01
Ovarian cancer	H3K4me3_peaks_Trynka.bed	4.16E-02	5.23E-02	2.56E-01	1.26E+00	6.14E+00	9.66E-01	-6.24E-10	6.46E-08	-9.66E-03	5.04E-01
Ovarian cancer	H3K4me3_Trynka.bed	1.33E-01	4.07E-01	2.26E-01	3.06E+00	1.70E+00	2.38E-01	-8.10E-09	3.60E-08	-2.25E-01	5.89E-01
Ovarian cancer	H3K4me3_Trynka.extend.500.bed	2.55E-01	9.14E-01	1.95E-01	3.58E+00	7.64E-01	9.59E-04	1.21E-08	2.06E-08	5.85E-01	2.79E-01
Ovarian cancer	H3K9ac_peaks_Trynka.bed	3.85E-02	-1.77E-01	2.60E-01	-4.60E+00	6.75E+00	3.82E-01	-7.38E-08	7.39E-08	-9.99E-01	8.41E-01
Ovarian cancer	H3K9ac_Trynka.bed	1.25E-01	2.27E-01	2.69E-01	1.81E+00	2.14E+00	7.06E-01	-4.29E-08	4.21E-08	-1.02E+00	8.46E-01
Ovarian cancer	H3K9ac_Trynka.extend.500.bed	2.30E-01	1.07E+00	2.05E-01	4.64E+00	8.94E-01	7.20E-05	5.53E-08	2.45E-08	2.25E+00	1.21E-02
Ovarian cancer	Intron_UCSC.bed	3.87E-01	6.30E-01	1.33E-01	1.62E+00	3.42E-01	4.90E-02	5.38E-08	1.30E-07	4.14E-01	3.39E-01
Ovarian cancer	Intron_UCSC.extend.500.bed	3.97E-01	6.09E-01	9.67E-02	1.54E+00	2.44E-01	1.80E-02	-5.06E-08	1.30E-07	-3.90E-01	6.52E-01
Ovarian cancer	Promoter_UCSC.bed	3.07E-02	3.63E-01	1.37E-01	1.18E+01	4.47E+00	1.54E-02	8.91E-08	1.46E-07	6.12E-01	2.70E-01
Ovarian cancer	Promoter_UCSC.extend.500.bed	3.81E-02	3.60E-01	1.04E-01	9.46E+00	2.73E+00	2.41E-03	-2.04E-08	1.19E-07	-1.71E-01	5.68E-01
Ovarian cancer	PromoterFlanking_Hoffman.bed	8.31E-03	7.52E-02	1.21E-01	9.06E+00	1.45E+01	5.66E-01	7.37E-08	1.48E-07	4.98E-01	3.09E-01
Ovarian cancer	PromoterFlanking_Hoffman.extend.500.bed	3.32E-02	5.35E-02	1.27E-01	1.61E+00	3.83E+00	8.73E-01	-6.45E-08	5.53E-08	-1.16E+00	8.78E-01
Ovarian cancer	Repressed_Hoffman.bed	4.61E-01	-2.75E-01	3.65E-01	-5.96E-01	7.92E-01	4.67E-02	-1.73E-08	1.78E-08	-9.73E-01	8.35E-01
Ovarian cancer	Repressed_Hoffman.extend.500.bed	7.19E-01	3.45E-01	1.31E-01	4.80E-01	1.82E-01	5.73E-03	2.20E-08	1.57E-08	1.41E+00	7.97E-02
Ovarian cancer	SuperEnhancer_Hnisz.bed	1.67E-01	3.60E-01	8.79E-02	2.15E+00	5.26E-01	4.16E-02	-4.04E-07	1.78E-07	-2.28E+00	9.89E-01

Phenotypes	Category	Proportion of SNPs	Proportion of h^2	Proportion of h^2 SE	Enrichment	Enrichment SE	Enrichment *P*-value	Coefficient	Coefficient SE	Coefficient *Z*-value	Coefficient *P*-value
Ovarian cancer	SuperEnhancer_Hnisz.extend.500.bed	1.70E-01	5.28E-01	8.96E-02	3.10E+00	5.26E-01	3.65E-04	4.03E-07	1.76E-07	2.29E+00	1.09E-02
Ovarian cancer	TFBS_ENCODE.bed	1.31E-01	7.21E-01	4.19E-01	5.50E+00	3.19E+00	1.70E-01	4.87E-08	4.45E-08	1.09E+00	1.37E-01
Ovarian cancer	TFBS_ENCODE.extend.500.bed	3.41E-01	1.04E+00	3.01E-01	3.06E+00	8.81E-01	2.56E-02	2.73E-09	2.09E-08	1.31E-01	4.48E-01
Ovarian cancer	Transcribed_Hoffman.bed	3.46E-01	8.55E-01	3.11E-01	2.47E+00	8.98E-01	7.87E-02	1.03E-08	1.37E-08	7.53E-01	2.26E-01
Ovarian cancer	Transcribed_Hoffman.extend.500.bed	7.62E-01	8.25E-01	1.81E-01	1.08E+00	2.37E-01	7.19E-01	-2.06E-09	1.12E-08	-1.84E-01	5.73E-01
Ovarian cancer	TSS_Hoffman.bed	1.78E-02	8.33E-02	1.51E-01	4.67E+00	8.46E+00	6.66E-01	-5.62E-08	1.21E-07	-4.64E-01	6.79E-01
Ovarian cancer	TSS_Hoffman.extend.500.bed	3.44E-02	3.00E-01	1.38E-01	8.75E+00	4.03E+00	6.11E-02	7.14E-08	8.13E-08	8.78E-01	1.90E-01
Ovarian cancer	UTR_3_UCSC.bed	1.12E-02	2.03E-01	7.94E-02	1.82E+01	7.10E+00	7.03E-03	2.21E-07	9.20E-08	2.40E+00	8.27E-03
Ovarian cancer	UTR_3_UCSC.extend.500.bed	2.65E-02	2.50E-01	8.50E-02	9.46E+00	3.21E+00	4.21E-03	-2.64E-09	4.29E-08	-6.15E-02	5.25E-01
Ovarian cancer	UTR_5_UCSC.bed	5.48E-03	-2.46E-02	6.43E-02	-4.49E+00	1.17E+01	6.31E-01	-1.03E-07	1.18E-07	-8.69E-01	8.08E-01
Ovarian cancer	UTR_5_UCSC.extend.500.bed	2.69E-02	2.49E-01	1.08E-01	9.28E+00	4.02E+00	4.43E-02	8.68E-08	4.48E-08	1.94E+00	2.64E-02
Ovarian cancer	WeakEnhancer_Hoffman.bed	2.10E-02	2.33E-01	1.86E-01	1.11E+01	8.88E+00	2.51E-01	8.97E-08	9.51E-08	9.43E-01	1.73E-01
Ovarian cancer	WeakEnhancer_Hoffman.extend.500.bed	8.88E-02	2.35E-01	1.93E-01	2.65E+00	2.18E+00	4.59E-01	-3.04E-08	3.61E-08	-8.44E-01	8.01E-01
