# Supplementary material for: Investigating the shared genetic architecture between breast and ovarian cancers
Source: Genet Mol Biol. 2024 Apr 15;47(2):e20230181. doi: 10.1590/1678-4685-GMB-2023-0181 (PMC11021043; doi:10.1590/1678-4685-GMB-2023-0181)
Supplement: Table S2 - [file 1415-4757-GMB-47-02-e20230181-s2.pdf]

Genetics and Molecular Biology

https://doi.org/10.1590/1678-4685-GMB-2023-0181

Supplementary Material to "Investigating the shared genetic architecture between breast and ovarian cancers"

Table S2 - The regions for each cancer that reach the $2.93\times e^{-5}$ threshold (*P*-values).

**Phenotypes**	**Chromosome**	**Region start**	**Region end**	**Num snp**	**Local_h2g**	**SE**	***Z*-value**	***P*-value**
Breast cancer	1	9365199	10806984	1818	6.48E-04	1.40E-04	4.64E+00	1.74E-06
Breast cancer	1	118839067	144977494	4504	9.70E-04	1.54E-04	6.29E+00	1.63E-10
Breast cancer	2	118367466	121303783	5106	7.53E-04	1.45E-04	5.21E+00	9.55E-08
Breast cancer	2	201576284	202818637	1445	6.55E-04	1.40E-04	4.68E+00	1.42E-06
Breast cancer	2	217715661	218395480	1257	3.03E-03	2.27E-04	1.34E+01	4.67E-41
Breast cancer	3	26877769	27840910	1896	1.63E-03	1.81E-04	9.01E+00	9.85E-20
Breast cancer	4	174264132	176570716	4129	5.45E-04	1.35E-04	4.05E+00	2.58E-05
Breast cancer	5	43983499	50163398	4696	2.36E-03	2.06E-04	1.14E+01	1.27E-30
Breast cancer	5	55417349	56621102	2294	2.84E-03	2.21E-04	1.28E+01	4.94E-38
Breast cancer	5	156628700	158825698	4258	8.11E-04	1.47E-04	5.51E+00	1.80E-08

**Phenotypes**	**Chromosome**	**Region start**	**Region end**	**Num snp**	**Local_h2g**	**SE**	***Z*-value**	***P*-value**
Breast cancer	6	151912703	153094496	2429	2.51E-03	2.11E-04	1.19E+01	6.12E-33
Breast cancer	8	29327896	31133729	2876	5.67E-04	1.36E-04	4.18E+00	1.45E-05
Breast cancer	8	75445064	76456542	1740	1.31E-03	1.68E-04	7.77E+00	3.93E-15
Breast cancer	8	126410917	128659111	4674	1.40E-03	1.72E-04	8.14E+00	1.96E-16
Breast cancer	9	20463534	22206559	3019	7.75E-04	1.46E-04	5.33E+00	5.00E-08
Breast cancer	9	110695062	112778024	4723	1.27E-03	1.67E-04	7.62E+00	1.31E-14
Breast cancer	10	63341695	65794114	4409	1.25E-03	1.66E-04	7.55E+00	2.20E-14
Breast cancer	10	78706814	80876749	3843	5.42E-04	1.34E-04	4.03E+00	2.78E-05
Breast cancer	10	122407323	123231465	1704	6.11E-04	1.38E-04	4.43E+00	4.62E-06
Breast cancer	10	123231465	123900545	1547	6.91E-03	3.21E-04	2.15E+01	1.08E-102
Breast cancer	11	1213590	3665481	6674	5.76E-04	1.36E-04	4.23E+00	1.17E-05
Breast cancer	11	68005825	69516130	2655	2.47E-03	2.10E-04	1.18E+01	1.93E-32
Breast cancer	12	27799773	29651255	3821	1.83E-03	1.88E-04	9.73E+00	1.08E-22
Breast cancer	12	115503216	117087471	3136	1.06E-03	1.58E-04	6.70E+00	1.06E-11
Breast cancer	14	35859593	38667725	3969	5.76E-04	1.36E-04	4.23E+00	1.16E-05
Breast cancer	14	67992317	71131957	5657	8.34E-04	1.48E-04	5.63E+00	9.24E-09
Breast cancer	14	91296860	93132299	3803	5.66E-04	1.36E-04	4.17E+00	1.49E-05
Breast cancer	16	52035823	53382572	2275	4.42E-03	2.65E-04	1.67E+01	5.59E-63
Breast cancer	16	53382572	55903774	5378	6.10E-04	1.38E-04	4.43E+00	4.79E-06
Breast cancer	16	80297374	81772536	3953	5.91E-04	1.37E-04	4.32E+00	7.76E-06
Breast cancer	17	51826118	53599432	3153	6.95E-04	1.42E-04	4.90E+00	4.73E-07
Breast cancer	18	24026191	25927682	3110	9.07E-04	1.51E-04	5.99E+00	1.08E-09

Phenotypes	Chromosome	Region start	Region end	Num snp	Local_h2g	SE	*Z*-value	*P*-value
Breast cancer	19	18409862	19877471	2190	7.73E-04	1.45E-04	5.31E+00	5.40E-08
Breast cancer	19	43862455	44744108	1721	5.70E-04	1.36E-04	4.20E+00	1.35E-05
Breast cancer	21	15950982	18053165	3271	7.01E-04	1.42E-04	4.94E+00	4.00E-07
Breast cancer	22	27834752	29651799	2479	5.50E-04	1.35E-04	4.08E+00	2.24E-05
Breast cancer	22	40545797	42690818	2707	8.90E-04	1.51E-04	5.90E+00	1.77E-09
Ovarian cancer	3	156008700	157312028	2278	2.29E-03	5.69E-04	4.03E+00	2.82E-05
