# Supplementary material for: Investigating the shared genetic architecture between breast and ovarian cancers
Source: Genet Mol Biol. 2024 Apr 15;47(2):e20230181. doi: 10.1590/1678-4685-GMB-2023-0181 (PMC11021043; doi:10.1590/1678-4685-GMB-2023-0181)
Supplement: Table S3 - [file 1415-4757-GMB-47-02-e20230181-s3.pdf]

Genetics and Molecular Biology

https://doi.org/10.1590/1678-4685-GMB-2023-0181

Supplementary Material to "Investigating the shared genetic architecture between breast and ovarian cancers"

Table S3 - Summary of SNP enrichment in tissue types for breast cancer and ovarian cancer.

**Phenotypes**	**Category**	**Coefficient**	**Coefficient SE**	**Coefficient *P*-value**
Brest cancer	Adipose_Subcutaneous	1.87E-09	1.72E-09	1.39E-01
Brest cancer	Adipose_Visceral_(Omentum)	-1.38E-09	1.86E-09	7.70E-01
Brest cancer	Adrenal_Gland	1.60E-09	1.96E-09	2.08E-01
Brest cancer	Artery_Aorta	-2.62E-09	1.76E-09	9.32E-01
Brest cancer	Artery_Coronary	-3.02E-09	1.61E-09	9.69E-01
Brest cancer	Artery_Tibial	9.86E-10	1.66E-09	2.77E-01
Brest cancer	Bladder	3.09E-09	2.00E-09	6.15E-02
Brest cancer	Brain_Amygdala	-4.81E-09	1.44E-09	1.00E+00
Brest cancer	Brain_Anterior_cingulate_cortex_(BA24)	-4.29E-09	1.39E-09	9.99E-01
Brest cancer	Brain_Caudate_(basal_ganglia)	-4.90E-09	1.46E-09	1.00E+00

Phenotypes	Category	Coefficient	Coefficient SE	Coefficient *P*-value
Brest cancer	Brain_Cerebellar_Hemisphere	-3.99E-09	1.60E-09	9.94E-01
Brest cancer	Brain_Cerebellum	-3.33E-09	1.52E-09	9.86E-01
Brest cancer	Brain_Cortex	-4.68E-09	1.38E-09	1.00E+00
Brest cancer	Brain_Frontal_Cortex_(BA9)	-4.43E-09	1.39E-09	9.99E-01
Brest cancer	Brain_Hippocampus	-3.91E-09	1.36E-09	9.98E-01
Brest cancer	Brain_Hypothalamus	-3.91E-09	1.48E-09	9.96E-01
Brest cancer	Brain_Nucleus_accumbens_(basal_ganglia)	-4.78E-09	1.50E-09	9.99E-01
Brest cancer	Brain_Putamen_(basal_ganglia)	-3.94E-09	1.45E-09	9.97E-01
Brest cancer	Brain_Spinal_cord_(cervical_c-1)	-4.97E-09	1.56E-09	9.99E-01
Brest cancer	Brain_Substantia_nigra	-4.45E-09	1.50E-09	9.98E-01
**Brest cancer**	**Breast_Mammary_Tissue**	**9.17E-09**	**2.06E-09**	**4.39E-06**
Brest cancer	Cells_EBV-transformed_lymphocytes	5.66E-09	2.20E-09	5.16E-03
Brest cancer	Cells_Transformed_fibroblasts	-4.11E-10	1.85E-09	5.88E-01
Brest cancer	Cervix_Ectocervix	3.11E-09	2.06E-09	6.59E-02
Brest cancer	Cervix_Endocervix	4.22E-09	2.23E-09	2.89E-02
Brest cancer	Colon_Sigmoid	8.03E-11	1.89E-09	4.83E-01
Brest cancer	Colon_Transverse	3.06E-10	2.14E-09	4.43E-01
Brest cancer	Esophagus_Gastroesophageal_Junction	-1.60E-09	1.81E-09	8.12E-01
Brest cancer	Esophagus_Mucosa	2.75E-09	2.13E-09	9.92E-02
Brest cancer	Esophagus_Muscularis	-2.33E-09	1.90E-09	8.90E-01
Brest cancer	Fallopian_Tube	5.96E-09	2.36E-09	5.78E-03
Brest cancer	Heart_Atrial_Appendage	1.13E-09	1.91E-09	2.77E-01

**Phenotypes**	**Category**	**Coefficient**	**Coefficient SE**	**Coefficient *P*-value**
Brest cancer	Heart_Left_Ventricle	1.68E-12	1.95E-09	5.00E-01
Brest cancer	Kidney_Cortex	2.32E-09	2.09E-09	1.34E-01
Brest cancer	Liver	3.77E-10	1.89E-09	4.21E-01
Brest cancer	Lung	-2.46E-09	2.01E-09	8.90E-01
Brest cancer	Minor_Salivary_Gland	5.74E-09	2.23E-09	5.07E-03
Brest cancer	Muscle_Skeletal	-2.96E-10	2.03E-09	5.58E-01
Brest cancer	Nerve_Tibial	-1.54E-11	1.83E-09	5.03E-01
Brest cancer	Ovary	3.28E-09	2.08E-09	5.75E-02
Brest cancer	Pancreas	5.87E-10	1.79E-09	3.72E-01
Brest cancer	Pituitary	-3.88E-09	1.70E-09	9.89E-01
Brest cancer	Prostate	3.85E-09	2.36E-09	5.09E-02
Brest cancer	Skin_Not_Sun_Exposed_(Suprapubic)	6.68E-09	2.36E-09	2.30E-03
Brest cancer	Skin_Sun_Exposed_(Lower_leg)	4.75E-09	2.31E-09	2.01E-02
Brest cancer	Small_Intestine_Terminal_Ileum	-2.22E-09	2.27E-09	8.36E-01
Brest cancer	Spleen	-2.54E-09	2.11E-09	8.86E-01
Brest cancer	Stomach	7.88E-10	2.09E-09	3.53E-01
Brest cancer	Testis	-2.91E-09	1.57E-09	9.68E-01
Brest cancer	Thyroid	2.93E-10	1.72E-09	4.33E-01
**Brest cancer**	**Uterus**	**9.56E-09**	**2.40E-09**	**3.32E-05**
Brest cancer	Vagina	5.64E-09	2.10E-09	3.66E-03
Brest cancer	Whole_Blood	-6.85E-10	2.06E-09	6.30E-01
Ovarian cancer	Adipose_Subcutaneous	2.14E-09	3.17E-09	2.50E-01

**Phenotypes**	**Category**	**Coefficient**	**Coefficient SE**	**Coefficient *P*-value**
Ovarian cancer	Adipose_Visceral_(Omentum)	2.32E-09	3.30E-09	2.41E-01
Ovarian cancer	Adrenal_Gland	-2.91E-09	3.22E-09	8.17E-01
Ovarian cancer	Artery_Aorta	1.32E-09	2.93E-09	3.26E-01
Ovarian cancer	Artery_Coronary	1.52E-09	3.03E-09	3.08E-01
Ovarian cancer	Artery_Tibial	3.50E-09	3.14E-09	1.32E-01
Ovarian cancer	Bladder	4.37E-09	3.03E-09	7.50E-02
Ovarian cancer	Brain_Amygdala	-1.05E-09	3.00E-09	6.37E-01
Ovarian cancer	Brain_Anterior_cingulate_cortex_(BA24)	-3.49E-09	2.93E-09	8.83E-01
Ovarian cancer	Brain_Caudate_(basal_ganglia)	-6.73E-10	2.98E-09	5.89E-01
Ovarian cancer	Brain_Cerebellar_Hemisphere	-3.61E-09	2.85E-09	8.97E-01
Ovarian cancer	Brain_Cerebellum	-3.70E-09	2.91E-09	8.98E-01
Ovarian cancer	Brain_Cortex	-6.19E-10	3.08E-09	5.80E-01
Ovarian cancer	Brain_Frontal_Cortex_(BA9)	-2.60E-09	3.05E-09	8.03E-01
Ovarian cancer	Brain_Hippocampus	-2.67E-09	3.00E-09	8.13E-01
Ovarian cancer	Brain_Hypothalamus	-4.26E-09	2.78E-09	9.37E-01
Ovarian cancer	Brain_Nucleus_accumbens_(basal_ganglia)	-1.44E-09	3.08E-09	6.80E-01
Ovarian cancer	Brain_Putamen_(basal_ganglia)	-1.31E-09	3.04E-09	6.67E-01
Ovarian cancer	Brain_Spinal_cord_(cervical_c-1)	1.43E-09	2.87E-09	3.09E-01
Ovarian cancer	Brain_Substantia_nigra	-1.83E-09	3.03E-09	7.27E-01
Ovarian cancer	Breast_Mammary_Tissue	3.50E-09	3.27E-09	1.42E-01
Ovarian cancer	Cells_EBV-transformed_lymphocytes	1.47E-09	3.97E-09	3.56E-01
Ovarian cancer	Cells_Transformed_fibroblasts	-3.02E-09	3.66E-09	7.95E-01

**Phenotypes**	**Category**	**Coefficient**	**Coefficient SE**	**Coefficient *P*-value**
Ovarian cancer	Cervix_Ectocervix	2.99E-09	3.60E-09	2.03E-01
Ovarian cancer	Cervix_Endocervix	-3.78E-10	4.09E-09	5.37E-01
Ovarian cancer	Colon_Sigmoid	6.39E-09	3.50E-09	3.38E-02
Ovarian cancer	Colon_Transverse	1.07E-09	3.98E-09	3.94E-01
Ovarian cancer	Esophagus_Gastroesophageal_Junction	3.71E-09	3.41E-09	1.38E-01
Ovarian cancer	Esophagus_Mucosa	-7.60E-10	3.72E-09	5.81E-01
Ovarian cancer	Esophagus_Muscularis	8.41E-09	3.55E-09	8.95E-03
Ovarian cancer	Fallopian_Tube	1.63E-09	4.18E-09	3.48E-01
Ovarian cancer	Heart_Atrial_Appendage	4.16E-09	3.26E-09	1.01E-01
Ovarian cancer	Heart_Left_Ventricle	2.33E-09	3.42E-09	2.48E-01
Ovarian cancer	Kidney_Cortex	1.62E-09	3.63E-09	3.28E-01
Ovarian cancer	Liver	6.07E-09	3.59E-09	4.56E-02
Ovarian cancer	Lung	-2.04E-09	3.54E-09	7.18E-01
Ovarian cancer	Minor_Salivary_Gland	-1.70E-09	3.40E-09	6.92E-01
Ovarian cancer	Muscle_Skeletal	3.94E-09	2.98E-09	9.31E-02
Ovarian cancer	Nerve_Tibial	-2.50E-10	3.58E-09	5.28E-01
Ovarian cancer	Ovary	-2.34E-10	3.62E-09	5.26E-01
Ovarian cancer	Pancreas	6.42E-09	3.39E-09	2.91E-02
Ovarian cancer	Pituitary	-3.61E-09	3.14E-09	8.75E-01
Ovarian cancer	Prostate	-4.69E-09	3.69E-09	8.98E-01
Ovarian cancer	Skin_Not_Sun_Exposed_(Suprapubic)	-1.89E-10	3.36E-09	5.22E-01
Ovarian cancer	Skin_Sun_Exposed_(Lower_leg)	1.02E-09	3.43E-09	3.84E-01

Phenotypes	Category	Coefficient	Coefficient SE	Coefficient *P*-value
Ovarian cancer	Small_Intestine_Terminal_Ileum	7.36E-10	3.53E-09	4.17E-01
Ovarian cancer	Spleen	-3.72E-09	4.28E-09	8.08E-01
Ovarian cancer	Stomach	-4.18E-10	3.78E-09	5.44E-01
Ovarian cancer	Testis	-3.89E-09	3.00E-09	9.03E-01
Ovarian cancer	Thyroid	-5.20E-09	3.33E-09	9.41E-01
Ovarian cancer	Uterus	5.09E-09	4.05E-09	1.05E-01
Ovarian cancer	Vagina	-1.61E-09	3.11E-09	6.98E-01
Ovarian cancer	Whole_Blood	6.82E-09	3.75E-09	3.45E-02
