# Supplementary material for: Investigating the shared genetic architecture between breast and ovarian cancers
Source: Genet Mol Biol. 2024 Apr 15;47(2):e20230181. doi: 10.1590/1678-4685-GMB-2023-0181 (PMC11021043; doi:10.1590/1678-4685-GMB-2023-0181)
Supplement: Table S4 - [file 1415-4757-GMB-47-02-e20230181-s4.pdf]

Genetics and Molecular Biology

https://doi.org/10.1590/1678-4685-GMB-2023-0181

Supplementary Material to "Investigating the shared genetic architecture between breast and ovarian cancers"

Table S4 - Summary of SNP enrichment in cell type groups for breast cancer and ovarian cancer.

**Phenotypes**	**Category**	**Proportion of SNPs**	**Proportion of h^2**	**Proportion of h^2 SE**	**Enrichment**	**Enrichment SE**	**Enrichment *P*-value**	**Coefficient**	**Coefficient SE**	**Coefficient *Z*-value**	**Coefficient *P*-value**
Brest cancer	Skeletal Muscle	1.04E-01	4.19E-01	4.67E-02	4.03E+00	4.50E-01	4.68E-10	2.06E-08	9.49E-09	2.17E+00	1.49E-02
**Brest cancer**	**Other**	**2.03E-01**	**7.53E-01**	**5.82E-02**	**3.72E+00**	**2.87E-01**	**2.53E-18**	**3.73E-08**	**8.12E-09**	**4.59E+00**	**2.21E-06**
Brest cancer	Liver	7.22E-02	2.76E-01	3.56E-02	3.82E+00	4.93E-01	1.92E-08	1.01E-08	9.03E-09	1.12E+00	1.32E-01
Brest cancer	Adrenal/Pancreas	9.36E-02	3.81E-01	4.13E-02	4.07E+00	4.42E-01	7.86E-11	2.05E-08	9.01E-09	2.28E+00	1.14E-02
**Brest cancer**	**Kidney**	**4.26E-02**	**2.57E-01**	**3.35E-02**	**6.03E+00**	**7.85E-01**	**4.25E-10**	**4.58E-08**	**1.37E-08**	**3.34E+00**	**4.23E-04**
Brest cancer	Immune	2.33E-01	5.15E-01	5.48E-02	2.21E+00	2.35E-01	5.26E-07	-2.15E-09	6.82E-09	-3.15E-01	6.24E-01
**Brest cancer**	**Gastrointestinal**	**1.68E-01**	**5.77E-01**	**5.53E-02**	**3.44E+00**	**3.30E-01**	**5.06E-12**	**2.39E-08**	**8.09E-09**	**2.95E+00**	**1.60E-03**
**Brest cancer**	**Connective Bone**	**1.15E-01**	**4.97E-01**	**4.58E-02**	**4.32E+00**	**3.98E-01**	**1.77E-14**	**3.26E-08**	**8.05E-09**	**4.05E+00**	**2.55E-05**
Brest cancer	Central Nervous System	1.49E-01	3.53E-01	4.43E-02	2.37E+00	2.98E-01	9.44E-06	-1.12E-08	6.48E-09	-1.72E+00	9.57E-01
**Brest cancer**	**Cardiovascular**	**1.11E-01**	**4.75E-01**	**5.03E-02**	**4.27E+00**	**4.53E-01**	**1.78E-11**	**3.05E-08**	**9.22E-09**	**3.31E+00**	**4.62E-04**

**Phenotypes**	**Category**	**Proportion of SNPs**	**Proportion of h^2**	**Proportion of h^2 SE**	**Enrichment**	**Enrichment SE**	**Enrichment *P*-value**	**Coefficient**	**Coefficient SE**	**Coefficient *Z*-value**	**Coefficient *P*-value**
Ovarian cancer	Skeletal Muscle	1.04E-01	2.58E-01	1.61E-01	2.48E+00	1.55E+00	3.62E-01	-8.40E-09	1.57E-08	-5.36E-01	7.04E-01
Ovarian cancer	Other	2.03E-01	4.65E-01	1.87E-01	2.29E+00	9.21E-01	1.81E-01	-8.68E-09	1.44E-08	-6.01E-01	7.26E-01
Ovarian cancer	Liver	7.22E-02	3.39E-01	1.25E-01	4.70E+00	1.73E+00	4.51E-02	1.58E-08	1.58E-08	9.98E-01	1.59E-01
Ovarian cancer	Adrenal/Pancreas	9.36E-02	3.75E-01	1.56E-01	4.01E+00	1.67E+00	8.78E-02	1.08E-08	1.74E-08	6.19E-01	2.68E-01
Ovarian cancer	Kidney	4.26E-02	4.19E-01	1.39E-01	9.84E+00	3.26E+00	1.42E-02	6.85E-08	2.78E-08	2.46E+00	6.87E-03
Ovarian cancer	Immune	2.33E-01	6.84E-01	2.10E-01	2.93E+00	9.02E-01	4.13E-02	-1.67E-09	1.45E-08	-1.15E-01	5.46E-01
Ovarian cancer	Gastrointestinal	1.68E-01	6.50E-01	2.08E-01	3.87E+00	1.24E+00	2.90E-02	1.44E-08	1.55E-08	9.29E-01	1.76E-01
Ovarian cancer	Connective Bone	1.15E-01	2.82E-01	1.46E-01	2.45E+00	1.27E+00	2.75E-01	-2.14E-08	1.43E-08	-1.50E+00	9.33E-01
Ovarian cancer	Central Nervous System	1.49E-01	3.14E-01	1.61E-01	2.11E+00	1.08E+00	3.21E-01	-1.12E-08	1.32E-08	-8.43E-01	8.00E-01
Ovarian cancer	Cardiovascular	1.11E-01	2.84E-01	1.62E-01	2.55E+00	1.46E+00	3.02E-01	-1.42E-09	1.94E-08	-7.34E-02	5.29E-01
