# Supplementary material for: Investigating the shared genetic architecture between breast and ovarian cancers
Source: Genet Mol Biol. 2024 Apr 15;47(2):e20230181. doi: 10.1590/1678-4685-GMB-2023-0181 (PMC11021043; doi:10.1590/1678-4685-GMB-2023-0181)
Supplement: Table S5 - [file 1415-4757-GMB-47-02-e20230181-s5.pdf]

Genetics and Molecular Biology

https://doi.org/10.1590/1678-4685-GMB-2023-0181

Supplementary Material to “Investigating the shared genetic architecture between breast and ovarian cancers”

Table S5 - Summary of SNP enrichment in cell types for breast cancer and ovarian cancer.

**Phenotypes**	**Mark**	**Category**	**Proportion of SNPs**	**Proportion of h^2**	**Proportion of h^2 SE**	**Enrichment**	**Enrichment SE**	**Enrichment *P*-value**	**Coefficient**	**Coefficient SE**	**Coefficient *Z*-value**	**Coefficient *P*-value**
**Brest cancer**	**H3K27ac**	**Adipose_nuclei**	**2.65E-02**	**1.49E-01**	**2.45E-02**	**5.61E+00**	**9.26E-01**	**8.16E-07**	**5.80E-08**	**1.53E-08**	**3.78E+00**	**7.74E-05**
Brest cancer	H3K27ac	Angular_gyrus	2.37E-02	-3.18E-03	1.64E-02	-1.35E-01	6.94E-01	1.02E-01	-4.28E-08	1.19E-08	-3.61E+00	1.00E+00
Brest cancer	H3K27ac	Anterior_caudate	2.16E-02	1.73E-02	1.89E-02	8.01E-01	8.74E-01	8.20E-01	-2.69E-08	1.49E-08	-1.80E+00	9.64E-01
Brest cancer	H3K27ac	CD14	2.84E-02	3.38E-02	1.71E-02	1.19E+00	6.00E-01	7.53E-01	-2.27E-08	1.05E-08	-2.17E+00	9.85E-01
Brest cancer	H3K27ac	CD19	3.66E-02	8.75E-02	3.01E-02	2.39E+00	8.24E-01	9.01E-02	8.88E-09	1.42E-08	6.25E-01	2.66E-01
Brest cancer	H3K27ac	CD20	2.87E-02	6.79E-02	2.24E-02	2.37E+00	7.80E-01	7.81E-02	7.71E-09	1.29E-08	5.96E-01	2.76E-01
Brest cancer	H3K27ac	CD25-_CD45RA+_naive	2.72E-02	2.10E-02	2.27E-02	7.72E-01	8.36E-01	7.85E-01	-2.80E-08	1.40E-08	-2.00E+00	9.77E-01
Brest cancer	H3K27ac	CD25-_IL17-_Th_stim_MACS	2.27E-02	1.64E-02	1.94E-02	7.25E-01	8.56E-01	7.49E-01	-2.79E-08	1.46E-08	-1.91E+00	9.72E-01
Brest cancer	H3K27ac	CD25-_IL17+_Th17_stim	2.72E-02	4.56E-02	2.45E-02	1.68E+00	9.03E-01	4.51E-01	-1.12E-08	1.54E-08	-7.31E-01	7.68E-01

Phenotypes	Mark	Category	Proportion of SNPs	Proportion of h^2	Proportion of h^2 SE	Enrichment	Enrichment SE	Enrichment *P*-value	Coefficient	Coefficient SE	Coefficient *Z*-value	Coefficient *P*-value
Brest cancer	H3K27ac	CD25+_CD127-_Treg	3.13E-02	5.16E-02	2.45E-02	1.65E+00	7.83E-01	4.08E-01	-4.34E-09	1.36E-08	-3.20E-01	6.25E-01
Brest cancer	H3K27ac	CD25int_CD127+_Tmem	3.21E-02	3.64E-02	3.13E-02	1.13E+00	9.74E-01	8.92E-01	-1.26E-08	1.67E-08	-7.55E-01	7.75E-01
Brest cancer	H3K27ac	CD3_primary	3.19E-02	2.23E-02	2.55E-02	6.97E-01	7.99E-01	7.05E-01	-2.63E-08	1.38E-08	-1.90E+00	9.72E-01
Brest cancer	H3K27ac	Chondrogenic_dif	2.65E-02	1.09E-01	2.08E-02	4.09E+00	7.85E-01	8.82E-05	2.78E-08	1.35E-08	2.06E+00	1.98E-02
Brest cancer	H3K27ac	Cingulate_gyrus	2.48E-02	1.67E-02	1.56E-02	6.74E-01	6.30E-01	6.05E-01	-2.75E-08	1.10E-08	-2.49E+00	9.94E-01
Brest cancer	H3K27ac	Colon_smooth_muscle	2.54E-02	1.03E-01	2.35E-02	4.05E+00	9.26E-01	1.30E-03	3.01E-08	1.56E-08	1.93E+00	2.68E-02
Brest cancer	H3K27ac	Colonic_mucosa	2.15E-02	9.59E-02	2.36E-02	4.45E+00	1.10E+00	1.84E-03	2.98E-08	1.85E-08	1.61E+00	5.39E-02
Brest cancer	H3K27ac	Duodenum_mucosa	2.59E-02	9.92E-02	2.74E-02	3.83E+00	1.06E+00	8.37E-03	2.40E-08	1.84E-08	1.31E+00	9.57E-02
Brest cancer	H3K27ac	Duodenum_smooth_muscle	2.09E-02	8.49E-02	2.01E-02	4.07E+00	9.62E-01	1.76E-03	2.48E-08	1.60E-08	1.55E+00	6.10E-02
Brest cancer	H3K27ac	Hippocampus_middle	2.04E-02	2.47E-02	1.56E-02	1.21E+00	7.66E-01	7.83E-01	-2.32E-08	1.31E-08	-1.77E+00	9.62E-01
Brest cancer	H3K27ac	Inferior_temporal_lobe	2.60E-02	-9.87E-04	1.68E-02	-3.79E-02	6.43E-01	1.07E-01	-3.87E-08	1.11E-08	-3.48E+00	1.00E+00
Brest cancer	H3K27ac	Kidney	2.51E-02	1.15E-01	2.19E-02	4.57E+00	8.70E-01	4.56E-05	3.67E-08	1.45E-08	2.53E+00	5.74E-03
Brest cancer	H3K27ac	Liver	2.61E-02	7.40E-02	1.88E-02	2.83E+00	7.21E-01	1.15E-02	6.25E-09	1.25E-08	5.02E-01	3.08E-01
Brest cancer	H3K27ac	Mid_frontal_lobe	1.94E-02	-2.51E-03	1.68E-02	-1.29E-01	8.66E-01	1.89E-01	-4.73E-08	1.46E-08	-3.24E+00	9.99E-01
Brest cancer	H3K27ac	Mobilized_CD34	3.18E-02	8.74E-02	2.90E-02	2.75E+00	9.13E-01	5.43E-02	1.32E-08	1.57E-08	8.41E-01	2.00E-01
Brest cancer	H3K27ac	Neurosphere	3.15E-02	8.39E-02	2.29E-02	2.66E+00	7.27E-01	2.56E-02	5.07E-09	1.22E-08	4.16E-01	3.39E-01
Brest cancer	H3K27ac	Osteoblast	2.86E-02	9.92E-02	1.95E-02	3.47E+00	6.83E-01	2.89E-04	1.55E-08	1.18E-08	1.31E+00	9.44E-02
Brest cancer	H3K27ac	Pancreatic_islets	2.80E-02	9.09E-02	1.85E-02	3.24E+00	6.61E-01	8.19E-04	1.44E-08	1.09E-08	1.32E+00	9.34E-02
Brest cancer	H3K27ac	Rectal_mucosa	2.20E-02	8.04E-02	2.26E-02	3.66E+00	1.03E+00	9.81E-03	1.69E-08	1.72E-08	9.79E-01	1.64E-01
Brest cancer	H3K27ac	Rectal_smooth_muscle	2.55E-02	8.94E-02	2.23E-02	3.50E+00	8.75E-01	5.07E-03	2.05E-08	1.46E-08	1.41E+00	8.00E-02

**Phenotypes**	**Mark**	**Category**	**Proportion of SNPs**	**Proportion of h^2**	**Proportion of h^2 SE**	**Enrichment**	**Enrichment SE**	**Enrichment *P*-value**	**Coefficient**	**Coefficient SE**	**Coefficient *Z*-value**	**Coefficient *P*-value**
Brest cancer	H3K27ac	Skeletal_muscle	2.37E-02	1.03E-01	2.38E-02	4.34E+00	1.00E+00	1.04E-03	3.24E-08	1.65E-08	1.96E+00	2.47E-02
Brest cancer	H3K27ac	Stomach_smooth_muscle	2.67E-02	8.63E-02	2.09E-02	3.24E+00	7.85E-01	5.00E-03	1.82E-08	1.31E-08	1.40E+00	8.15E-02
Brest cancer	H3K27ac	Substantia_nigra	2.51E-02	2.63E-02	1.54E-02	1.04E+00	6.12E-01	9.42E-01	-1.99E-08	1.09E-08	-1.83E+00	9.66E-01
Brest cancer	H3K27ac	Th0	3.18E-02	3.73E-02	2.29E-02	1.17E+00	7.20E-01	8.10E-01	-1.56E-08	1.29E-08	-1.21E+00	8.87E-01
Brest cancer	H3K27ac	Th1	3.19E-02	6.63E-02	2.23E-02	2.08E+00	7.00E-01	1.24E-01	3.06E-09	1.25E-08	2.44E-01	4.03E-01
Brest cancer	H3K27ac	Th2	3.17E-02	6.09E-02	2.38E-02	1.92E+00	7.51E-01	2.22E-01	1.51E-09	1.35E-08	1.12E-01	4.55E-01
**Brest cancer**	**H3K4me1**	**Adipose_nuclei**	**8.79E-02**	**4.38E-01**	**4.81E-02**	**4.99E+00**	**5.47E-01**	**4.28E-12**	**4.42E-08**	**1.17E-08**	**3.78E+00**	**7.93E-05**
Brest cancer	H3K4me1	Angular_gyrus	1.32E-02	4.04E-02	1.92E-02	3.06E+00	1.46E+00	1.58E-01	-2.70E-08	2.63E-08	-1.03E+00	8.48E-01
Brest cancer	H3K4me1	Anterior_caudate	3.90E-02	1.22E-01	3.03E-02	3.12E+00	7.78E-01	6.47E-03	-1.31E-08	1.52E-08	-8.60E-01	8.05E-01
Brest cancer	H3K4me1	Breast_fibroblast_primary	1.03E-02	1.04E-01	2.43E-02	1.01E+01	2.35E+00	9.37E-05	1.06E-07	3.79E-08	2.80E+00	2.58E-03
Brest cancer	H3K4me1	Breast_luminal_epithelial	1.92E-03	3.89E-02	1.41E-02	2.03E+01	7.35E+00	9.13E-03	2.63E-07	1.19E-07	2.21E+00	1.35E-02
Brest cancer	H3K4me1	Breast_myoepithelial	4.76E-02	2.54E-01	3.70E-02	5.34E+00	7.78E-01	7.75E-08	4.91E-08	1.54E-08	3.19E+00	7.18E-04
Brest cancer	H3K4me1	Breast_vHMEC	3.43E-02	1.98E-01	3.43E-02	5.78E+00	1.00E+00	2.15E-06	4.80E-08	1.84E-08	2.60E+00	4.65E-03
Brest cancer	H3K4me1	CD14_primary	4.01E-02	1.63E-01	2.86E-02	4.07E+00	7.12E-01	2.58E-05	9.80E-09	1.19E-08	8.23E-01	2.05E-01
Brest cancer	H3K4me1	CD15_primary	3.64E-02	1.47E-01	2.50E-02	4.03E+00	6.85E-01	1.53E-05	7.06E-09	1.08E-08	6.52E-01	2.57E-01
Brest cancer	H3K4me1	CD19_primary_(BI)	4.09E-02	1.86E-01	3.21E-02	4.56E+00	7.86E-01	8.43E-06	1.56E-08	1.41E-08	1.11E+00	1.34E-01
Brest cancer	H3K4me1	CD19_primary_(UW)	4.10E-02	1.63E-01	3.16E-02	3.98E+00	7.69E-01	1.41E-04	9.24E-09	1.42E-08	6.50E-01	2.58E-01
Brest cancer	H3K4me1	CD3_primary_(BI)	2.99E-02	9.70E-02	2.61E-02	3.24E+00	8.71E-01	1.08E-02	-9.59E-09	1.50E-08	-6.38E-01	7.38E-01
Brest cancer	H3K4me1	CD3_primary_(UW)	3.65E-02	1.16E-01	2.97E-02	3.18E+00	8.15E-01	8.58E-03	-1.57E-08	1.43E-08	-1.10E+00	8.64E-01
Brest cancer	H3K4me1	CD34_primary	3.24E-02	1.67E-01	3.64E-02	5.17E+00	1.12E+00	2.18E-04	1.93E-08	2.05E-08	9.40E-01	1.74E-01

Phenotypes	Mark	Category	Proportion of SNPs	Proportion of h^2	Proportion of h^2 SE	Enrichment	Enrichment SE	Enrichment *P*-value	Coefficient	Coefficient SE	Coefficient *Z*-value	Coefficient *P*-value
Brest cancer	H3K4me1	CD4_memory_primary	4.12E-02	1.12E-01	2.58E-02	2.72E+00	6.25E-01	6.53E-03	-1.29E-08	1.13E-08	-1.14E+00	8.72E-01
Brest cancer	H3K4me1	CD4_naive_primary	3.24E-02	8.78E-02	2.51E-02	2.70E+00	7.72E-01	2.89E-02	-1.68E-08	1.34E-08	-1.25E+00	8.94E-01
Brest cancer	H3K4me1	CD4+_CD25-_CD45R0+_memory_primary	3.45E-02	1.09E-01	2.63E-02	3.17E+00	7.61E-01	4.97E-03	-1.23E-08	1.32E-08	-9.31E-01	8.24E-01
Brest cancer	H3K4me1	CD4+_CD25-_CD45RA+_naive_primary	3.84E-02	1.13E-01	2.57E-02	2.95E+00	6.67E-01	4.03E-03	-1.28E-08	1.11E-08	-1.16E+00	8.76E-01
Brest cancer	H3K4me1	CD4+_CD25-_IL17-_PMA_Ionomycin_stim_MACS_Th_sprimary	5.24E-02	1.55E-01	2.75E-02	2.96E+00	5.25E-01	2.47E-04	-7.69E-09	9.28E-09	-8.29E-01	7.96E-01
Brest cancer	H3K4me1	CD4+_CD25-_IL17+_PMA_Ionomycin_stim_Th17_primary	3.95E-02	1.37E-01	2.85E-02	3.46E+00	7.21E-01	7.65E-04	-4.12E-09	1.28E-08	-3.21E-01	6.26E-01
Brest cancer	H3K4me1	CD4+_CD25-_Th_primary	4.31E-02	1.42E-01	2.86E-02	3.29E+00	6.63E-01	6.94E-04	-1.08E-08	1.12E-08	-9.62E-01	8.32E-01
Brest cancer	H3K4me1	CD4+_CD25+_CD127-_Treg_primary	2.86E-02	1.15E-01	2.66E-02	4.01E+00	9.30E-01	1.46E-03	-3.93E-09	1.60E-08	-2.46E-01	5.97E-01
Brest cancer	H3K4me1	CD4+_CD25int_CD127+_Tmem_primary	1.59E-02	6.91E-02	2.25E-02	4.36E+00	1.42E+00	1.90E-02	-4.46E-09	2.36E-08	-1.89E-01	5.75E-01
Brest cancer	H3K4me1	CD56_primary	3.49E-02	1.14E-01	3.14E-02	3.27E+00	8.99E-01	1.29E-02	-1.51E-08	1.56E-08	-9.64E-01	8.33E-01
Brest cancer	H3K4me1	CD8_memory_primary	3.18E-02	8.65E-02	2.48E-02	2.72E+00	7.78E-01	2.90E-02	-1.61E-08	1.37E-08	-1.18E+00	8.81E-01
Brest cancer	H3K4me1	CD8_naive_primary_(BI)	3.19E-02	8.59E-02	2.57E-02	2.70E+00	8.06E-01	3.71E-02	-1.85E-08	1.37E-08	-1.35E+00	9.12E-01
Brest cancer	H3K4me1	CD8_naive_primary_(UCSF-UBC)	2.48E-02	6.61E-02	2.45E-02	2.66E+00	9.87E-01	9.47E-02	-2.40E-08	1.73E-08	-1.39E+00	9.17E-01
Brest cancer	H3K4me1	Cingulate_gyrus	4.10E-02	1.02E-01	2.75E-02	2.48E+00	6.70E-01	2.67E-02	-2.72E-08	1.30E-08	-2.09E+00	9.82E-01
Brest cancer	H3K4me1	Colon_smooth_muscle	3.52E-02	1.99E-01	3.10E-02	5.67E+00	8.80E-01	4.29E-07	4.59E-08	1.61E-08	2.85E+00	2.18E-03
Brest cancer	H3K4me1	Colonic_mucosa	2.17E-02	1.48E-01	2.96E-02	6.81E+00	1.37E+00	2.71E-05	5.20E-08	2.39E-08	2.17E+00	1.49E-02
Brest cancer	H3K4me1	Duodenum_Mucosa	4.83E-02	2.28E-01	3.65E-02	4.72E+00	7.55E-01	1.22E-06	2.77E-08	1.50E-08	1.85E+00	3.24E-02
Brest cancer	H3K4me1	Duodenum_smooth_muscle	6.00E-03	7.75E-02	2.13E-02	1.29E+01	3.55E+00	9.08E-04	1.48E-07	6.03E-08	2.46E+00	6.98E-03
Brest cancer	H3K4me1	Esophagus	5.79E-03	4.93E-02	2.22E-02	8.51E+00	3.83E+00	5.05E-02	6.73E-08	6.31E-08	1.07E+00	1.43E-01
Brest cancer	H3K4me1	Fetal_adrenal	4.34E-02	2.14E-01	3.57E-02	4.94E+00	8.23E-01	3.74E-06	2.83E-08	1.58E-08	1.79E+00	3.65E-02

Phenotypes	Mark	Category	Proportion of SNPs	Proportion of h^2	Proportion of h^2 SE	Enrichment	Enrichment SE	Enrichment *P*-value	Coefficient	Coefficient SE	Coefficient *Z*-value	Coefficient *P*-value
Brest cancer	H3K4me1	Fetal_brain	3.32E-02	6.91E-02	1.76E-02	2.09E+00	5.32E-01	4.00E-02	2.91E-09	1.10E-08	2.64E-01	3.96E-01
Brest cancer	H3K4me1	Fetal_heart	4.15E-02	1.76E-01	2.74E-02	4.25E+00	6.62E-01	1.47E-06	1.97E-08	1.13E-08	1.74E+00	4.06E-02
Brest cancer	H3K4me1	Fetal_large_intestine	3.04E-02	1.68E-01	2.89E-02	5.51E+00	9.50E-01	3.23E-06	4.36E-08	1.69E-08	2.57E+00	5.03E-03
Brest cancer	H3K4me1	Fetal_leg_muscle	4.09E-02	2.05E-01	3.84E-02	5.03E+00	9.41E-01	4.64E-05	2.84E-08	1.76E-08	1.61E+00	5.35E-02
Brest cancer	H3K4me1	Fetal_lung	6.99E-02	3.36E-01	4.17E-02	4.80E+00	5.97E-01	4.95E-09	3.71E-08	1.09E-08	3.40E+00	3.37E-04
Brest cancer	H3K4me1	Fetal_placenta	2.53E-02	1.71E-01	3.03E-02	6.77E+00	1.20E+00	2.92E-06	6.00E-08	2.18E-08	2.75E+00	3.00E-03
Brest cancer	H3K4me1	Fetal_small_intestine	3.38E-02	1.86E-01	2.96E-02	5.52E+00	8.76E-01	5.37E-07	4.40E-08	1.58E-08	2.78E+00	2.71E-03
Brest cancer	H3K4me1	Fetal_stomach	3.43E-02	1.79E-01	3.43E-02	5.21E+00	9.98E-01	6.48E-05	2.88E-08	1.79E-08	1.61E+00	5.37E-02
Brest cancer	H3K4me1	Fetal_thymus	3.62E-02	1.24E-01	2.75E-02	3.42E+00	7.61E-01	1.69E-03	-8.90E-09	1.25E-08	-7.10E-01	7.61E-01
Brest cancer	H3K4me1	Fetal_trunk_muscle	4.14E-02	1.51E-01	3.77E-02	3.65E+00	9.10E-01	4.76E-03	-3.88E-09	1.67E-08	-2.32E-01	5.92E-01
Brest cancer	H3K4me1	Gastric	1.36E-02	6.73E-02	2.50E-02	4.95E+00	1.84E+00	3.20E-02	1.20E-08	3.14E-08	3.83E-01	3.51E-01
Brest cancer	H3K4me1	Hippocampus_middle	5.18E-02	1.46E-01	3.25E-02	2.83E+00	6.28E-01	3.94E-03	-2.13E-08	1.22E-08	-1.75E+00	9.60E-01
Brest cancer	H3K4me1	Inferior_temporal_lobe	4.76E-02	1.11E-01	3.02E-02	2.34E+00	6.35E-01	3.53E-02	-3.29E-08	1.23E-08	-2.68E+00	9.96E-01
Brest cancer	H3K4me1	Kidney	8.50E-03	8.59E-02	1.92E-02	1.01E+01	2.26E+00	4.76E-05	1.05E-07	3.84E-08	2.73E+00	3.16E-03
Brest cancer	H3K4me1	Left_Ventricle	2.66E-02	1.28E-01	2.58E-02	4.81E+00	9.70E-01	1.03E-04	2.54E-08	1.75E-08	1.45E+00	7.31E-02
Brest cancer	H3K4me1	Liver_(BI)	5.50E-02	2.08E-01	3.23E-02	3.78E+00	5.87E-01	2.64E-06	9.41E-09	1.11E-08	8.50E-01	1.98E-01
Brest cancer	H3K4me1	Liver_(UCSD)	1.46E-03	2.35E-02	1.16E-02	1.61E+01	7.96E+00	5.76E-02	1.92E-07	1.28E-07	1.49E+00	6.76E-02
Brest cancer	H3K4me1	Lung	1.75E-02	1.17E-01	2.45E-02	6.68E+00	1.40E+00	5.10E-05	5.26E-08	2.50E-08	2.11E+00	1.76E-02
Brest cancer	H3K4me1	Mid_frontal_lobe	3.47E-03	2.22E-02	1.18E-02	6.40E+00	3.39E+00	1.13E-01	2.75E-08	5.87E-08	4.69E-01	3.20E-01
Brest cancer	H3K4me1	Mobilized_CD34_primary	6.92E-02	2.66E-01	4.17E-02	3.85E+00	6.03E-01	3.09E-06	8.45E-09	1.11E-08	7.64E-01	2.22E-01

Phenotypes	Mark	Category	Proportion of SNPs	Proportion of h^2	Proportion of h^2 SE	Enrichment	Enrichment SE	Enrichment *P*-value	Coefficient	Coefficient SE	Coefficient *Z*-value	Coefficient *P*-value
Brest cancer	H3K4me1	Ovary	8.71E-03	4.67E-02	2.12E-02	5.36E+00	2.44E+00	7.41E-02	3.37E-08	4.12E-08	8.19E-01	2.06E-01
Brest cancer	H3K4me1	Pancreas	3.00E-02	1.55E-01	2.97E-02	5.18E+00	9.91E-01	3.50E-05	3.05E-08	1.88E-08	1.63E+00	5.20E-02
Brest cancer	H3K4me1	Pancreatic_islets	6.28E-03	3.91E-02	1.81E-02	6.23E+00	2.88E+00	6.69E-02	4.64E-08	4.80E-08	9.66E-01	1.67E-01
Brest cancer	H3K4me1	Pancreatic_islets	8.52E-03	3.95E-02	1.72E-02	4.64E+00	2.01E+00	7.16E-02	3.10E-08	3.30E-08	9.41E-01	1.73E-01
Brest cancer	H3K4me1	Penis_foreskin_fibroblast_primary	7.73E-02	3.44E-01	3.90E-02	4.45E+00	5.04E-01	1.35E-10	2.67E-08	1.06E-08	2.53E+00	5.75E-03
Brest cancer	H3K4me1	Penis_foreskin_keratinocyte_primary	6.09E-02	2.81E-01	3.97E-02	4.62E+00	6.52E-01	5.04E-08	2.57E-08	1.26E-08	2.03E+00	2.12E-02
Brest cancer	H3K4me1	Penis_foreskin_melanocyte_primary	6.53E-02	2.44E-01	3.39E-02	3.73E+00	5.19E-01	4.19E-07	1.10E-08	9.26E-09	1.19E+00	1.17E-01
Brest cancer	H3K4me1	Peripheralblood_mononuclear_primary	8.97E-03	6.90E-02	2.94E-02	7.69E+00	3.27E+00	4.27E-02	9.68E-09	5.53E-08	1.75E-01	4.31E-01
Brest cancer	H3K4me1	Placenta_amnion	4.05E-03	3.67E-02	1.78E-02	9.05E+00	4.39E+00	6.94E-02	7.85E-08	7.30E-08	1.07E+00	1.41E-01
Brest cancer	H3K4me1	Placenta_chorion	7.54E-03	3.70E-02	1.90E-02	4.91E+00	2.52E+00	1.23E-01	2.40E-08	4.19E-08	5.73E-01	2.83E-01
Brest cancer	H3K4me1	Psoas_muscle	3.93E-03	3.13E-02	1.66E-02	7.97E+00	4.23E+00	9.94E-02	6.63E-08	6.82E-08	9.72E-01	1.66E-01
Brest cancer	H3K4me1	Rectal_mucosa	3.50E-02	1.95E-01	3.55E-02	5.57E+00	1.01E+00	8.50E-06	3.62E-08	1.87E-08	1.93E+00	2.65E-02
Brest cancer	H3K4me1	Rectal_smooth_muscle	9.29E-03	9.01E-02	2.05E-02	9.69E+00	2.20E+00	1.21E-04	9.65E-08	3.75E-08	2.58E+00	4.99E-03
Brest cancer	H3K4me1	Right_atrium	9.22E-03	9.14E-02	2.00E-02	9.92E+00	2.17E+00	4.71E-05	1.06E-07	3.67E-08	2.90E+00	1.89E-03
Brest cancer	H3K4me1	Right_ventricle	8.10E-04	1.18E-02	9.87E-03	1.45E+01	1.22E+01	2.67E-01	1.57E-07	1.95E-07	8.04E-01	2.11E-01
Brest cancer	H3K4me1	Sigmoid_colon	3.01E-03	1.82E-02	1.54E-02	6.05E+00	5.11E+00	3.23E-01	2.19E-08	8.55E-08	2.56E-01	3.99E-01
Brest cancer	H3K4me1	Skeletal_muscle	6.68E-02	2.96E-01	3.89E-02	4.43E+00	5.82E-01	2.15E-08	2.35E-08	1.19E-08	1.98E+00	2.36E-02
Brest cancer	H3K4me1	Small_intestine	1.04E-03	1.29E-02	9.82E-03	1.25E+01	9.45E+00	2.27E-01	1.39E-07	1.53E-07	9.05E-01	1.83E-01
Brest cancer	H3K4me1	Spleen	4.59E-02	1.98E-01	3.46E-02	4.31E+00	7.54E-01	2.07E-05	1.84E-08	1.44E-08	1.28E+00	1.00E-01
Brest cancer	H3K4me1	Stomach_mucosa	3.36E-02	2.12E-01	3.31E-02	6.31E+00	9.85E-01	1.28E-07	5.73E-08	1.82E-08	3.16E+00	7.98E-04

Phenotypes	Mark	Category	Proportion of SNPs	Proportion of h^2	Proportion of h^2 SE	Enrichment	Enrichment SE	Enrichment *P*-value	Coefficient	Coefficient SE	Coefficient *Z*-value	Coefficient *P*-value
Brest cancer	H3K4me1	Stomach_smooth_muscle	2.39E-02	1.62E-01	2.87E-02	6.76E+00	1.20E+00	3.54E-06	5.36E-08	2.24E-08	2.39E+00	8.43E-03
Brest cancer	H3K4me1	Substantia_nigra	3.79E-02	1.13E-01	2.65E-02	2.97E+00	6.98E-01	4.74E-03	-1.20E-08	1.32E-08	-9.06E-01	8.18E-01
Brest cancer	H3K4me1	Thymus	8.80E-03	2.04E-02	1.50E-02	2.32E+00	1.71E+00	4.40E-01	-3.76E-08	2.87E-08	-1.31E+00	9.05E-01
Brest cancer	H3K4me3	Adipose_nuclei	2.93E-02	1.92E-01	2.98E-02	6.56E+00	1.02E+00	1.03E-07	3.63E-08	1.70E-08	2.14E+00	1.62E-02
Brest cancer	H3K4me3	Angular_gyrus	1.07E-02	8.49E-02	2.41E-02	7.91E+00	2.24E+00	2.66E-03	-3.68E-09	4.87E-08	-7.56E-02	5.30E-01
Brest cancer	H3K4me3	Anterior_caudate	1.92E-02	1.38E-01	3.14E-02	7.19E+00	1.64E+00	2.38E-04	1.30E-08	3.41E-08	3.80E-01	3.52E-01
Brest cancer	H3K4me3	Aorta	8.53E-03	1.12E-01	2.16E-02	1.32E+01	2.53E+00	2.92E-06	1.03E-07	4.82E-08	2.14E+00	1.63E-02
Brest cancer	H3K4me3	Breast_fibroblast_primary	5.80E-03	8.43E-02	2.14E-02	1.45E+01	3.70E+00	2.98E-04	1.07E-07	6.80E-08	1.58E+00	5.74E-02
Brest cancer	H3K4me3	Breast_myoepithelial	1.39E-02	1.58E-01	3.30E-02	1.13E+01	2.37E+00	2.41E-05	1.10E-07	4.95E-08	2.23E+00	1.30E-02
Brest cancer	H3K4me3	Breast_vHMEC	7.75E-03	1.03E-01	2.37E-02	1.33E+01	3.06E+00	7.17E-05	1.10E-07	5.59E-08	1.96E+00	2.49E-02
Brest cancer	H3K4me3	CD14_primary	8.37E-03	9.93E-02	2.74E-02	1.19E+01	3.27E+00	1.02E-03	7.53E-08	5.41E-08	1.39E+00	8.21E-02
Brest cancer	H3K4me3	CD15_primary	1.40E-02	1.27E-01	3.00E-02	9.07E+00	2.14E+00	2.16E-04	5.11E-08	3.37E-08	1.51E+00	6.51E-02
Brest cancer	H3K4me3	CD19_primary_(BI)	1.21E-02	1.20E-01	2.98E-02	9.92E+00	2.46E+00	3.54E-04	5.70E-08	4.55E-08	1.25E+00	1.05E-01
Brest cancer	H3K4me3	CD19_primary_(UW)	8.86E-03	8.98E-02	2.94E-02	1.01E+01	3.32E+00	6.24E-03	3.41E-08	5.96E-08	5.73E-01	2.83E-01
Brest cancer	H3K4me3	CD3_primary_(BI)	1.42E-02	8.10E-02	2.81E-02	5.71E+00	1.98E+00	1.90E-02	-4.10E-08	3.67E-08	-1.12E+00	8.68E-01
Brest cancer	H3K4me3	CD3_primary_(UW)	1.06E-02	7.25E-02	2.84E-02	6.82E+00	2.67E+00	3.18E-02	-3.93E-08	4.89E-08	-8.02E-01	7.89E-01
Brest cancer	H3K4me3	CD34_primary	1.07E-02	1.30E-01	2.81E-02	1.22E+01	2.63E+00	3.56E-05	1.03E-07	4.99E-08	2.07E+00	1.93E-02
Brest cancer	H3K4me3	CD4_memory_primary	1.17E-02	8.67E-02	2.72E-02	7.40E+00	2.32E+00	6.60E-03	-1.30E-08	4.14E-08	-3.13E-01	6.23E-01
Brest cancer	H3K4me3	CD4_naive_primary	1.27E-02	8.01E-02	2.80E-02	6.33E+00	2.21E+00	1.75E-02	-3.38E-08	3.92E-08	-8.64E-01	8.06E-01
Brest cancer	H3K4me3	CD4_primary	1.27E-02	8.15E-02	2.90E-02	6.44E+00	2.29E+00	1.91E-02	-3.81E-08	4.03E-08	-9.44E-01	8.27E-01

Phenotypes	Mark	Category	Proportion of SNPs	Proportion of h^2	Proportion of h^2 SE	Enrichment	Enrichment SE	Enrichment *P*-value	Coefficient	Coefficient SE	Coefficient *Z*-value	Coefficient *P*-value
Brest cancer	H3K4me3	CD4+_CD25-_CD45R0+_memory_primary	8.79E-03	7.89E-02	2.45E-02	8.98E+00	2.79E+00	4.93E-03	1.23E-08	5.07E-08	2.42E-01	4.04E-01
Brest cancer	H3K4me3	CD4+_CD25-_CD45RA+_naive_primary	1.31E-02	8.47E-02	3.06E-02	6.48E+00	2.35E+00	2.12E-02	-3.25E-08	4.25E-08	-7.65E-01	7.78E-01
Brest cancer	H3K4me3	CD4+_CD25-_IL17-_PMA_Ionomycin_stim_MACS_Th_sprimary	1.72E-02	1.07E-01	2.83E-02	6.24E+00	1.64E+00	1.56E-03	-1.27E-08	2.91E-08	-4.37E-01	6.69E-01
Brest cancer	H3K4me3	CD4+_CD25-_IL17+_PMA_Ionomycin_stim_Th17_primary	1.26E-02	1.08E-01	2.87E-02	8.56E+00	2.28E+00	1.01E-03	2.04E-08	3.92E-08	5.20E-01	3.01E-01
Brest cancer	H3K4me3	CD4+_CD25-_Th_primary	1.27E-02	9.31E-02	3.09E-02	7.33E+00	2.43E+00	1.05E-02	-1.16E-08	4.54E-08	-2.55E-01	6.01E-01
Brest cancer	H3K4me3	CD4+_CD25+_CD127-_Treg_primary	1.42E-02	1.02E-01	3.03E-02	7.17E+00	2.13E+00	4.41E-03	-8.26E-09	3.75E-08	-2.20E-01	5.87E-01
Brest cancer	H3K4me3	CD4+_CD25int_CD127+_Tmem_primary	1.08E-02	9.18E-02	2.95E-02	8.47E+00	2.73E+00	6.97E-03	5.50E-09	5.02E-08	1.10E-01	4.56E-01
Brest cancer	H3K4me3	CD56_primary	8.42E-03	8.21E-02	2.76E-02	9.75E+00	3.28E+00	8.53E-03	2.18E-08	5.97E-08	3.64E-01	3.58E-01
Brest cancer	H3K4me3	CD8_memory_primary	1.11E-02	8.59E-02	2.78E-02	7.71E+00	2.50E+00	8.32E-03	-3.51E-09	4.56E-08	-7.69E-02	5.31E-01
Brest cancer	H3K4me3	CD8_naive_primary_(BI)	1.14E-02	7.97E-02	2.86E-02	6.98E+00	2.51E+00	1.89E-02	-2.56E-08	4.65E-08	-5.51E-01	7.09E-01
Brest cancer	H3K4me3	CD8_naive_primary_(UCSF-UBC)	6.19E-03	6.07E-02	2.23E-02	9.80E+00	3.61E+00	1.64E-02	6.60E-09	6.19E-08	1.07E-01	4.58E-01
Brest cancer	H3K4me3	CD8_primary	1.04E-02	7.14E-02	2.79E-02	6.87E+00	2.69E+00	3.14E-02	-3.73E-08	4.95E-08	-7.53E-01	7.74E-01
Brest cancer	H3K4me3	Cingulate_gyrus	1.77E-02	1.19E-01	2.84E-02	6.71E+00	1.61E+00	5.52E-04	-4.85E-09	3.27E-08	-1.48E-01	5.59E-01
Brest cancer	H3K4me3	Colon_smooth_muscle	1.44E-02	1.74E-01	2.90E-02	1.21E+01	2.01E+00	1.47E-07	1.27E-07	3.89E-08	3.28E+00	5.25E-04
Brest cancer	H3K4me3	Colonic_mucosa	1.20E-02	1.58E-01	3.06E-02	1.31E+01	2.55E+00	3.93E-06	1.44E-07	5.13E-08	2.81E+00	2.48E-03
Brest cancer	H3K4me3	Duodenum_Mucosa	2.02E-02	1.82E-01	3.18E-02	9.01E+00	1.58E+00	7.58E-07	7.38E-08	3.24E-08	2.28E+00	1.14E-02
Brest cancer	H3K4me3	Duodenum_smooth_muscle	2.03E-02	1.56E-01	2.56E-02	7.70E+00	1.26E+00	2.08E-07	4.28E-08	2.15E-08	1.99E+00	2.33E-02
Brest cancer	H3K4me3	Esophagus	8.70E-03	1.14E-01	2.77E-02	1.31E+01	3.18E+00	1.93E-04	1.04E-07	5.82E-08	1.79E+00	3.70E-02
Brest cancer	H3K4me3	Fetal_adrenal	9.83E-03	1.11E-01	2.63E-02	1.13E+01	2.67E+00	1.89E-04	7.31E-08	5.44E-08	1.34E+00	8.93E-02
Brest cancer	H3K4me3	Fetal_brain	1.29E-02	1.01E-01	2.71E-02	7.84E+00	2.11E+00	1.64E-03	8.38E-09	4.28E-08	1.96E-01	4.22E-01

Phenotypes	Mark	Category	Proportion of SNPs	Proportion of h^2	Proportion of h^2 SE	Enrichment	Enrichment SE	Enrichment *P*-value	Coefficient	Coefficient SE	Coefficient *Z*-value	Coefficient *P*-value
Brest cancer	H3K4me3	Fetal_brain	4.09E-03	3.66E-02	1.76E-02	8.95E+00	4.31E+00	6.67E-02	8.56E-09	8.21E-08	1.04E-01	4.58E-01
Brest cancer	H3K4me3	Fetal_heart	5.82E-03	5.85E-02	1.81E-02	1.01E+01	3.11E+00	3.49E-03	4.91E-08	5.63E-08	8.71E-01	1.92E-01
Brest cancer	H3K4me3	Fetal_large_intestine	1.10E-02	1.37E-01	2.62E-02	1.25E+01	2.39E+00	4.16E-06	1.19E-07	4.56E-08	2.60E+00	4.68E-03
Brest cancer	H3K4me3	Fetal_leg_muscle	9.82E-03	1.35E-01	2.78E-02	1.38E+01	2.83E+00	1.49E-05	1.33E-07	5.31E-08	2.50E+00	6.20E-03
Brest cancer	H3K4me3	Fetal_lung	1.07E-02	1.15E-01	2.41E-02	1.08E+01	2.25E+00	2.79E-05	7.72E-08	4.55E-08	1.70E+00	4.47E-02
Brest cancer	H3K4me3	Fetal_placenta	8.25E-03	1.15E-01	2.77E-02	1.40E+01	3.36E+00	1.59E-04	1.30E-07	6.29E-08	2.06E+00	1.97E-02
Brest cancer	H3K4me3	Fetal_small_intestine	1.12E-02	1.36E-01	2.72E-02	1.22E+01	2.43E+00	1.11E-05	1.11E-07	4.68E-08	2.37E+00	8.85E-03
Brest cancer	H3K4me3	Fetal_stomach	1.02E-02	1.32E-01	2.80E-02	1.29E+01	2.74E+00	3.38E-05	1.16E-07	5.62E-08	2.06E+00	1.99E-02
Brest cancer	H3K4me3	Fetal_thymus	9.28E-03	8.91E-02	2.50E-02	9.60E+00	2.70E+00	1.77E-03	2.63E-08	4.87E-08	5.39E-01	2.95E-01
Brest cancer	H3K4me3	Fetal_trunk_muscle	8.87E-03	1.14E-01	2.69E-02	1.29E+01	3.03E+00	1.49E-04	1.01E-07	5.54E-08	1.83E+00	3.37E-02
Brest cancer	H3K4me3	Gastric	5.91E-03	8.89E-02	2.41E-02	1.50E+01	4.08E+00	6.62E-04	1.19E-07	7.26E-08	1.63E+00	5.13E-02
Brest cancer	H3K4me3	Germinal_matrix	1.16E-02	9.35E-02	2.68E-02	8.09E+00	2.32E+00	2.86E-03	4.81E-09	4.94E-08	9.74E-02	4.61E-01
Brest cancer	H3K4me3	Hippocampus_middle	1.89E-02	1.31E-01	2.91E-02	6.93E+00	1.54E+00	1.82E-04	1.83E-09	3.06E-08	5.99E-02	4.76E-01
Brest cancer	H3K4me3	Inferior_temporal_lobe	1.75E-02	1.14E-01	2.93E-02	6.50E+00	1.68E+00	1.32E-03	-8.69E-09	3.36E-08	-2.59E-01	6.02E-01
Brest cancer	H3K4me3	Kidney	1.74E-02	1.88E-01	2.88E-02	1.08E+01	1.65E+00	9.53E-09	1.11E-07	3.37E-08	3.29E+00	5.06E-04
Brest cancer	H3K4me3	Left_Ventricle	8.83E-03	1.06E-01	2.49E-02	1.20E+01	2.82E+00	1.61E-04	7.70E-08	5.28E-08	1.46E+00	7.24E-02
Brest cancer	H3K4me3	Liver_(BI)	1.63E-02	1.15E-01	2.15E-02	7.09E+00	1.32E+00	4.54E-06	2.26E-08	2.30E-08	9.84E-01	1.63E-01
Brest cancer	H3K4me3	Liver_(UCSD)	1.19E-02	1.21E-01	2.55E-02	1.01E+01	2.15E+00	3.24E-05	5.84E-08	4.01E-08	1.46E+00	7.24E-02
Brest cancer	H3K4me3	Lung	5.63E-03	9.13E-02	2.43E-02	1.62E+01	4.33E+00	5.64E-04	1.42E-07	7.88E-08	1.81E+00	3.54E-02
Brest cancer	H3K4me3	Mid_frontal_lobe	1.67E-02	1.21E-01	3.02E-02	7.27E+00	1.81E+00	7.91E-04	7.14E-09	3.82E-08	1.87E-01	4.26E-01

Phenotypes	Mark	Category	Proportion of SNPs	Proportion of h^2	Proportion of h^2 SE	Enrichment	Enrichment SE	Enrichment *P*-value	Coefficient	Coefficient SE	Coefficient *Z*-value	Coefficient *P*-value
Brest cancer	H3K4me3	Mobilized_CD34_primary	2.26E-02	1.73E-01	4.04E-02	7.67E+00	1.79E+00	2.47E-04	3.74E-08	3.60E-08	1.04E+00	1.49E-01
Brest cancer	H3K4me3	Ovary	9.60E-03	1.12E-01	2.56E-02	1.17E+01	2.66E+00	9.16E-05	7.76E-08	5.75E-08	1.35E+00	8.84E-02
Brest cancer	H3K4me3	Pancreas	8.35E-03	9.24E-02	2.32E-02	1.11E+01	2.78E+00	3.88E-04	5.32E-08	5.53E-08	9.62E-01	1.68E-01
Brest cancer	H3K4me3	Pancreatic_islets	1.14E-02	1.08E-01	2.66E-02	9.49E+00	2.34E+00	3.93E-04	3.96E-08	4.76E-08	8.34E-01	2.02E-01
Brest cancer	H3K4me3	Pancreatic_islets	1.01E-02	5.86E-02	2.29E-02	5.82E+00	2.28E+00	3.68E-02	2.57E-09	4.12E-08	6.22E-02	4.75E-01
Brest cancer	H3K4me3	Penis_foreskin_fibroblast_primary	3.74E-02	2.42E-01	3.74E-02	6.47E+00	1.00E+00	1.97E-07	5.65E-08	2.05E-08	2.76E+00	2.89E-03
Brest cancer	H3K4me3	Penis_foreskin_keratinocyte_primary	1.92E-02	1.70E-01	3.54E-02	8.84E+00	1.84E+00	3.73E-05	6.69E-08	3.58E-08	1.87E+00	3.09E-02
Brest cancer	H3K4me3	Penis_foreskin_melanocyte_primary	1.79E-02	1.50E-01	3.39E-02	8.40E+00	1.90E+00	1.61E-04	5.32E-08	3.53E-08	1.51E+00	6.61E-02
Brest cancer	H3K4me3	Peripheralblood_mononuclear_primary	9.72E-03	9.15E-02	3.05E-02	9.41E+00	3.14E+00	8.69E-03	1.82E-08	5.81E-08	3.14E-01	3.77E-01
Brest cancer	H3K4me3	Placenta_amnion	6.38E-03	1.18E-01	2.59E-02	1.85E+01	4.05E+00	2.73E-05	2.06E-07	7.40E-08	2.79E+00	2.63E-03
Brest cancer	H3K4me3	Placenta_chorion	8.67E-03	1.08E-01	2.72E-02	1.24E+01	3.14E+00	3.15E-04	9.84E-08	5.92E-08	1.66E+00	4.84E-02
Brest cancer	H3K4me3	Psoas_muscle	9.91E-03	1.06E-01	2.46E-02	1.07E+01	2.48E+00	1.64E-04	5.63E-08	4.99E-08	1.13E+00	1.30E-01
Brest cancer	H3K4me3	Rectal_mucosa	1.84E-02	1.86E-01	3.58E-02	1.01E+01	1.95E+00	5.97E-06	9.47E-08	4.23E-08	2.24E+00	1.25E-02
Brest cancer	H3K4me3	Rectal_smooth_muscle	1.30E-02	1.73E-01	3.19E-02	1.33E+01	2.45E+00	1.53E-06	1.56E-07	5.17E-08	3.01E+00	1.29E-03
Brest cancer	H3K4me3	Right_atrium	1.11E-02	1.35E-01	2.55E-02	1.22E+01	2.30E+00	3.27E-06	1.01E-07	4.75E-08	2.14E+00	1.63E-02
Brest cancer	H3K4me3	Right_ventricle	1.08E-02	1.42E-01	2.64E-02	1.31E+01	2.44E+00	2.09E-06	1.23E-07	4.85E-08	2.54E+00	5.49E-03
Brest cancer	H3K4me3	Sigmoid_colon	5.57E-03	1.06E-01	2.37E-02	1.90E+01	4.25E+00	3.19E-05	2.04E-07	7.23E-08	2.82E+00	2.40E-03
Brest cancer	H3K4me3	Skeletal_muscle	2.21E-02	1.89E-01	3.27E-02	8.58E+00	1.48E+00	8.24E-07	6.20E-08	3.03E-08	2.04E+00	2.05E-02
Brest cancer	H3K4me3	Small_intestine	4.95E-03	1.05E-01	2.37E-02	2.12E+01	4.79E+00	3.51E-05	2.40E-07	8.23E-08	2.91E+00	1.78E-03
Brest cancer	H3K4me3	Spleen	4.17E-03	7.87E-02	2.32E-02	1.89E+01	5.55E+00	1.38E-03	1.77E-07	9.45E-08	1.88E+00	3.02E-02

Phenotypes	Mark	Category	Proportion of SNPs	Proportion of h^2	Proportion of h^2 SE	Enrichment	Enrichment SE	Enrichment *P*-value	Coefficient	Coefficient SE	Coefficient *Z*-value	Coefficient *P*-value
Brest cancer	H3K4me3	Stomach_mucosa	5.91E-03	6.73E-02	2.16E-02	1.14E+01	3.65E+00	5.00E-03	4.81E-08	6.94E-08	6.93E-01	2.44E-01
Brest cancer	H3K4me3	Stomach_smooth_muscle	2.01E-02	1.99E-01	3.26E-02	9.92E+00	1.62E+00	1.80E-07	9.07E-08	3.31E-08	2.74E+00	3.11E-03
Brest cancer	H3K4me3	Substantia_nigra	1.44E-02	1.08E-01	2.58E-02	7.49E+00	1.79E+00	3.62E-04	3.29E-09	3.43E-08	9.57E-02	4.62E-01
Brest cancer	H3K4me3	Treg_primary	1.78E-02	9.11E-02	2.36E-02	5.12E+00	1.33E+00	2.28E-03	-9.09E-09	2.43E-08	-3.74E-01	6.46E-01
**Brest cancer**	**H3K9ac**	**Adipose_nuclei**	**2.86E-02**	**2.66E-01**	**3.86E-02**	**9.31E+00**	**1.35E+00**	**1.43E-09**	**1.10E-07**	**2.61E-08**	**4.20E+00**	**1.32E-05**
Brest cancer	H3K9ac	Angular_gyrus	1.10E-02	8.67E-02	2.42E-02	7.90E+00	2.21E+00	2.18E-03	2.13E-08	4.49E-08	4.73E-01	3.18E-01
Brest cancer	H3K9ac	Anterior_caudate	1.46E-02	1.25E-01	2.76E-02	8.58E+00	1.89E+00	8.78E-05	5.04E-08	3.63E-08	1.39E+00	8.26E-02
Brest cancer	H3K9ac	Breast_myoepithelial	4.64E-03	5.26E-02	1.84E-02	1.13E+01	3.97E+00	9.36E-03	5.74E-08	6.81E-08	8.44E-01	1.99E-01
Brest cancer	H3K9ac	CD8_naive_primary_(UCSF-UBC)	2.18E-03	1.98E-02	1.42E-02	9.04E+00	6.51E+00	2.20E-01	-1.93E-08	1.10E-07	-1.75E-01	5.69E-01
Brest cancer	H3K9ac	Cingulate_gyrus	1.92E-02	1.25E-01	2.85E-02	6.49E+00	1.48E+00	2.81E-04	1.26E-08	2.98E-08	4.22E-01	3.36E-01
Brest cancer	H3K9ac	Colon_smooth_muscle	5.67E-03	8.66E-02	1.84E-02	1.53E+01	3.24E+00	1.41E-05	1.43E-07	5.51E-08	2.59E+00	4.73E-03
Brest cancer	H3K9ac	Colonic_mucosa	1.81E-02	1.86E-01	3.26E-02	1.02E+01	1.79E+00	5.08E-07	1.04E-07	3.28E-08	3.18E+00	7.35E-04
Brest cancer	H3K9ac	Duodenum_Mucosa	1.51E-02	1.64E-01	3.00E-02	1.09E+01	1.98E+00	1.11E-06	1.07E-07	3.64E-08	2.93E+00	1.68E-03
Brest cancer	H3K9ac	Fetal_brain	1.27E-02	1.14E-01	2.80E-02	8.99E+00	2.21E+00	3.91E-04	5.45E-08	4.36E-08	1.25E+00	1.06E-01
Brest cancer	H3K9ac	Fetal_heart	2.05E-02	1.62E-01	3.02E-02	7.89E+00	1.47E+00	3.85E-06	4.62E-08	2.56E-08	1.80E+00	3.56E-02
Brest cancer	H3K9ac	Fetal_kidney	5.04E-03	7.70E-02	2.01E-02	1.53E+01	3.98E+00	5.00E-04	1.26E-07	7.71E-08	1.63E+00	5.11E-02
Brest cancer	H3K9ac	Fetal_lung	1.30E-02	1.65E-01	3.09E-02	1.27E+01	2.38E+00	2.24E-06	1.32E-07	4.45E-08	2.96E+00	1.55E-03
Brest cancer	H3K9ac	Hippocampus_middle	1.79E-02	1.20E-01	2.92E-02	6.71E+00	1.63E+00	5.73E-04	1.36E-08	3.11E-08	4.37E-01	3.31E-01
Brest cancer	H3K9ac	Inferior_temporal_lobe	1.68E-02	1.07E-01	2.74E-02	6.36E+00	1.62E+00	1.16E-03	2.73E-09	3.15E-08	8.68E-02	4.65E-01
Brest cancer	H3K9ac	Kidney	1.08E-02	1.58E-01	3.03E-02	1.47E+01	2.82E+00	1.33E-06	1.75E-07	5.48E-08	3.19E+00	7.08E-04

Phenotypes	Mark	Category	Proportion of SNPs	Proportion of h^2	Proportion of h^2 SE	Enrichment	Enrichment SE	Enrichment *P*-value	Coefficient	Coefficient SE	Coefficient *Z*-value	Coefficient *P*-value
Brest cancer	H3K9ac	Liver_(BI)	1.41E-02	1.28E-01	2.43E-02	9.07E+00	1.72E+00	3.86E-06	5.89E-08	2.90E-08	2.03E+00	2.12E-02
Brest cancer	H3K9ac	Mid_frontal_lobe	1.51E-02	9.66E-02	2.64E-02	6.41E+00	1.75E+00	2.41E-03	-1.88E-09	3.53E-08	-5.34E-02	5.21E-01
Brest cancer	H3K9ac	Pancreatic_islets	6.24E-03	5.42E-02	2.01E-02	8.67E+00	3.22E+00	1.85E-02	1.04E-09	5.89E-08	1.77E-02	4.93E-01
Brest cancer	H3K9ac	Penis_foreskin_keratinocyte_primary	3.27E-02	1.58E-01	4.06E-02	4.84E+00	1.24E+00	2.33E-03	2.10E-09	2.22E-08	9.45E-02	4.62E-01
Brest cancer	H3K9ac	Peripheralblood_mononuclear_primary	1.32E-02	1.05E-01	3.37E-02	7.94E+00	2.55E+00	7.22E-03	1.05E-08	4.41E-08	2.38E-01	4.06E-01
Brest cancer	H3K9ac	Rectal_mucosa	1.61E-02	1.71E-01	3.19E-02	1.06E+01	1.98E+00	2.10E-06	1.00E-07	3.62E-08	2.77E+00	2.82E-03
Brest cancer	H3K9ac	Rectal_smooth_muscle	3.57E-03	5.60E-02	1.96E-02	1.57E+01	5.48E+00	8.48E-03	1.11E-07	9.59E-08	1.16E+00	1.23E-01
Brest cancer	H3K9ac	Skeletal_muscle	2.76E-02	2.07E-01	3.77E-02	7.48E+00	1.37E+00	4.28E-06	5.44E-08	2.58E-08	2.11E+00	1.74E-02
Brest cancer	H3K9ac	Stomach_mucosa	1.31E-02	1.47E-01	2.80E-02	1.13E+01	2.14E+00	2.08E-06	9.99E-08	3.71E-08	2.69E+00	3.54E-03
Brest cancer	H3K9ac	Stomach_smooth_muscle	1.40E-02	1.46E-01	2.66E-02	1.04E+01	1.90E+00	1.84E-06	8.51E-08	3.35E-08	2.54E+00	5.60E-03
Brest cancer	H3K9ac	Substantia_nigra	1.69E-02	1.16E-01	2.79E-02	6.87E+00	1.65E+00	4.29E-04	1.95E-08	3.03E-08	6.44E-01	2.60E-01
Ovarian cancer	H3K27ac	Adipose_nuclei	2.65E-02	4.59E-02	6.01E-02	1.73E+00	2.27E+00	7.45E-01	-2.05E-08	2.05E-08	-9.99E-01	8.41E-01
Ovarian cancer	H3K27ac	Angular_gyrus	2.37E-02	8.75E-02	6.82E-02	3.70E+00	2.88E+00	3.45E-01	6.68E-09	2.37E-08	2.82E-01	3.89E-01
Ovarian cancer	H3K27ac	Anterior_caudate	2.16E-02	1.45E-01	8.03E-02	6.72E+00	3.72E+00	1.20E-01	3.28E-08	3.09E-08	1.06E+00	1.44E-01
Ovarian cancer	H3K27ac	CD14	2.84E-02	2.34E-02	6.44E-02	8.22E-01	2.26E+00	9.37E-01	-2.72E-08	2.07E-08	-1.31E+00	9.06E-01
Ovarian cancer	H3K27ac	CD19	3.66E-02	8.15E-02	8.55E-02	2.23E+00	2.34E+00	5.95E-01	-1.74E-08	2.06E-08	-8.45E-01	8.01E-01
Ovarian cancer	H3K27ac	CD20	2.87E-02	4.31E-02	6.61E-02	1.50E+00	2.30E+00	8.26E-01	-2.57E-08	2.08E-08	-1.24E+00	8.92E-01
Ovarian cancer	H3K27ac	CD25-_CD45RA+_naive	2.72E-02	1.58E-01	7.87E-02	5.83E+00	2.90E+00	8.24E-02	1.34E-08	2.56E-08	5.25E-01	3.00E-01
Ovarian cancer	H3K27ac	CD25-_IL17-_Th_stim_MACS	2.27E-02	1.07E-01	6.01E-02	4.72E+00	2.65E+00	1.59E-01	6.95E-09	2.39E-08	2.91E-01	3.85E-01
Ovarian cancer	H3K27ac	CD25-_IL17+_Th17_stim	2.72E-02	3.89E-02	9.73E-02	1.43E+00	3.58E+00	9.03E-01	-2.43E-08	3.18E-08	-7.64E-01	7.77E-01

Phenotypes	Mark	Category	Proportion of SNPs	Proportion of h^2	Proportion of h^2 SE	Enrichment	Enrichment SE	Enrichment *P*-value	Coefficient	Coefficient SE	Coefficient *Z*-value	Coefficient *P*-value
Ovarian cancer	H3K27ac	CD25+_CD127-_Treg	3.13E-02	1.33E-01	8.16E-02	4.26E+00	2.61E+00	1.99E-01	1.48E-09	2.36E-08	6.28E-02	4.75E-01
Ovarian cancer	H3K27ac	CD25int_CD127+_Tmem	3.21E-02	9.58E-02	9.11E-02	2.98E+00	2.84E+00	4.82E-01	-7.60E-09	2.59E-08	-2.94E-01	6.16E-01
Ovarian cancer	H3K27ac	CD3_primary	3.19E-02	1.29E-01	7.60E-02	4.03E+00	2.38E+00	1.97E-01	3.19E-09	2.23E-08	1.43E-01	4.43E-01
Ovarian cancer	H3K27ac	Chondrogenic_dif	2.65E-02	1.05E-01	7.06E-02	3.96E+00	2.66E+00	2.60E-01	-2.45E-09	2.41E-08	-1.02E-01	5.41E-01
Ovarian cancer	H3K27ac	Cingulate_gyrus	2.48E-02	7.73E-02	6.82E-02	3.12E+00	2.76E+00	4.44E-01	2.69E-09	2.28E-08	1.18E-01	4.53E-01
Ovarian cancer	H3K27ac	Colon_smooth_muscle	2.54E-02	1.50E-01	7.33E-02	5.90E+00	2.89E+00	1.01E-01	2.21E-08	2.36E-08	9.38E-01	1.74E-01
Ovarian cancer	H3K27ac	Colonic_mucosa	2.15E-02	2.28E-01	1.07E-01	1.06E+01	4.97E+00	5.62E-02	5.63E-08	4.10E-08	1.37E+00	8.49E-02
Ovarian cancer	H3K27ac	Duodenum_mucosa	2.59E-02	3.28E-01	1.50E-01	1.27E+01	5.80E+00	4.28E-02	7.81E-08	4.85E-08	1.61E+00	5.35E-02
Ovarian cancer	H3K27ac	Duodenum_smooth_muscle	2.09E-02	1.42E-01	8.42E-02	6.82E+00	4.04E+00	1.44E-01	2.61E-08	3.41E-08	7.67E-01	2.21E-01
Ovarian cancer	H3K27ac	Hippocampus_middle	2.04E-02	9.48E-02	6.73E-02	4.64E+00	3.29E+00	2.75E-01	1.28E-08	2.73E-08	4.69E-01	3.20E-01
Ovarian cancer	H3K27ac	Inferior_temporal_lobe	2.60E-02	9.58E-02	7.00E-02	3.68E+00	2.69E+00	3.17E-01	7.07E-09	2.24E-08	3.16E-01	3.76E-01
Ovarian cancer	H3K27ac	Kidney	2.51E-02	2.98E-01	9.66E-02	1.18E+01	3.84E+00	7.39E-03	7.04E-08	3.28E-08	2.14E+00	1.60E-02
Ovarian cancer	H3K27ac	Liver	2.61E-02	1.60E-01	6.73E-02	6.12E+00	2.57E+00	4.26E-02	1.43E-08	2.25E-08	6.36E-01	2.62E-01
Ovarian cancer	H3K27ac	Mid_frontal_lobe	1.94E-02	9.36E-02	7.08E-02	4.83E+00	3.65E+00	2.89E-01	1.41E-08	3.10E-08	4.54E-01	3.25E-01
Ovarian cancer	H3K27ac	Mobilized_CD34	3.18E-02	2.17E-02	9.85E-02	6.82E-01	3.10E+00	9.18E-01	-3.32E-08	2.65E-08	-1.25E+00	8.94E-01
Ovarian cancer	H3K27ac	Neurosphere	3.15E-02	1.58E-01	8.09E-02	5.01E+00	2.57E+00	1.19E-01	1.19E-08	2.17E-08	5.47E-01	2.92E-01
Ovarian cancer	H3K27ac	Osteoblast	2.86E-02	1.21E-01	6.55E-02	4.24E+00	2.29E+00	1.37E-01	-3.84E-10	1.97E-08	-1.95E-02	5.08E-01
Ovarian cancer	H3K27ac	Pancreatic_islets	2.80E-02	1.35E-01	7.58E-02	4.82E+00	2.70E+00	1.43E-01	3.77E-09	2.23E-08	1.69E-01	4.33E-01
Ovarian cancer	H3K27ac	Rectal_mucosa	2.20E-02	2.06E-01	1.07E-01	9.36E+00	4.87E+00	8.50E-02	4.52E-08	4.03E-08	1.12E+00	1.31E-01
Ovarian cancer	H3K27ac	Rectal_smooth_muscle	2.55E-02	1.54E-01	6.89E-02	6.03E+00	2.70E+00	7.21E-02	2.39E-08	2.29E-08	1.04E+00	1.48E-01

Phenotypes	Mark	Category	Proportion of SNPs	Proportion of h^2	Proportion of h^2 SE	Enrichment	Enrichment SE	Enrichment *P*-value	Coefficient	Coefficient SE	Coefficient *Z*-value	Coefficient *P*-value
Ovarian cancer	H3K27ac	Skeletal_muscle	2.37E-02	1.59E-01	7.00E-02	6.71E+00	2.95E+00	5.69E-02	2.56E-08	2.34E-08	1.10E+00	1.37E-01
Ovarian cancer	H3K27ac	Stomach_smooth_muscle	2.67E-02	4.14E-02	6.89E-02	1.55E+00	2.59E+00	8.32E-01	-1.85E-08	2.20E-08	-8.43E-01	8.00E-01
Ovarian cancer	H3K27ac	Substantia_nigra	2.51E-02	9.04E-02	6.74E-02	3.60E+00	2.68E+00	3.35E-01	4.93E-09	2.22E-08	2.22E-01	4.12E-01
Ovarian cancer	H3K27ac	Th0	3.18E-02	1.90E-01	1.02E-01	5.99E+00	3.20E+00	1.13E-01	2.30E-08	2.69E-08	8.55E-01	1.96E-01
Ovarian cancer	H3K27ac	Th1	3.19E-02	1.62E-01	1.01E-01	5.08E+00	3.17E+00	1.98E-01	1.36E-08	2.71E-08	5.02E-01	3.08E-01
Ovarian cancer	H3K27ac	Th2	3.17E-02	1.40E-01	1.08E-01	4.41E+00	3.42E+00	3.18E-01	8.49E-09	2.94E-08	2.89E-01	3.86E-01
Ovarian cancer	H3K4me1	Adipose_nuclei	8.79E-02	1.54E-01	1.56E-01	1.75E+00	1.78E+00	6.75E-01	-1.88E-08	1.89E-08	-9.95E-01	8.40E-01
Ovarian cancer	H3K4me1	Angular_gyrus	1.32E-02	4.82E-02	7.81E-02	3.65E+00	5.92E+00	6.55E-01	1.91E-08	5.45E-08	3.51E-01	3.63E-01
Ovarian cancer	H3K4me1	Anterior_caudate	3.90E-02	2.96E-02	1.17E-01	7.60E-01	3.00E+00	9.36E-01	-1.95E-08	3.10E-08	-6.28E-01	7.35E-01
Ovarian cancer	H3K4me1	Breast_fibroblast_primary	1.03E-02	2.72E-02	6.92E-02	2.63E+00	6.71E+00	8.06E-01	-8.38E-09	5.79E-08	-1.45E-01	5.58E-01
Ovarian cancer	H3K4me1	Breast_luminal_epithelial	1.92E-03	-3.01E-02	6.05E-02	-1.57E+01	3.16E+01	5.81E-01	-1.45E-07	2.44E-07	-5.95E-01	7.24E-01
Ovarian cancer	H3K4me1	Breast_myoepithelial	4.76E-02	1.25E-02	1.14E-01	2.63E-01	2.39E+00	7.53E-01	-2.91E-08	2.21E-08	-1.32E+00	9.06E-01
Ovarian cancer	H3K4me1	Breast_vHMEC	3.43E-02	-1.89E-01	1.24E-01	-5.52E+00	3.63E+00	5.68E-02	-9.56E-08	3.51E-08	-2.72E+00	9.97E-01
Ovarian cancer	H3K4me1	CD14_primary	4.01E-02	9.78E-02	9.77E-02	2.44E+00	2.44E+00	5.57E-01	-1.35E-08	2.28E-08	-5.92E-01	7.23E-01
Ovarian cancer	H3K4me1	CD15_primary	3.64E-02	9.99E-02	8.53E-02	2.74E+00	2.34E+00	4.53E-01	-8.44E-09	2.28E-08	-3.70E-01	6.44E-01
Ovarian cancer	H3K4me1	CD19_primary_(BI)	4.09E-02	1.42E-01	9.42E-02	3.49E+00	2.31E+00	2.96E-01	-1.24E-08	2.29E-08	-5.40E-01	7.06E-01
Ovarian cancer	H3K4me1	CD19_primary_(UW)	4.10E-02	1.01E-01	8.94E-02	2.47E+00	2.18E+00	5.10E-01	-1.65E-08	2.15E-08	-7.68E-01	7.79E-01
Ovarian cancer	H3K4me1	CD3_primary_(BI)	2.99E-02	1.64E-01	8.05E-02	5.47E+00	2.69E+00	1.09E-01	1.84E-08	2.53E-08	7.25E-01	2.34E-01
Ovarian cancer	H3K4me1	CD3_primary_(UW)	3.65E-02	1.87E-01	9.28E-02	5.12E+00	2.55E+00	1.26E-01	2.32E-08	2.66E-08	8.71E-01	1.92E-01
Ovarian cancer	H3K4me1	CD34_primary	3.24E-02	6.37E-02	1.24E-01	1.97E+00	3.82E+00	7.99E-01	-2.54E-08	3.57E-08	-7.10E-01	7.61E-01

Phenotypes	Mark	Category	Proportion of SNPs	Proportion of h^2	Proportion of h^2 SE	Enrichment	Enrichment SE	Enrichment *P*-value	Coefficient	Coefficient SE	Coefficient *Z*-value	Coefficient *P*-value
Ovarian cancer	H3K4me1	CD4_memory_primary	4.12E-02	2.45E-01	8.96E-02	5.94E+00	2.17E+00	3.01E-02	2.87E-08	2.20E-08	1.31E+00	9.56E-02
Ovarian cancer	H3K4me1	CD4_naive_primary	3.24E-02	1.70E-01	8.05E-02	5.23E+00	2.48E+00	1.00E-01	1.82E-08	2.46E-08	7.43E-01	2.29E-01
Ovarian cancer	H3K4me1	CD4+_CD25-_CD45R0+_memory_primary	3.45E-02	1.97E-01	8.58E-02	5.72E+00	2.49E+00	7.12E-02	2.28E-08	2.45E-08	9.31E-01	1.76E-01
Ovarian cancer	H3K4me1	CD4+_CD25-_CD45RA+_naive_primary	3.84E-02	2.10E-01	8.95E-02	5.47E+00	2.33E+00	6.81E-02	2.45E-08	2.26E-08	1.08E+00	1.40E-01
Ovarian cancer	H3K4me1	CD4+_CD25-_IL17-_PMA_Ionomycin_stim_MACS_Th_sprimary	5.24E-02	2.79E-01	1.00E-01	5.31E+00	1.91E+00	3.20E-02	2.76E-08	1.90E-08	1.45E+00	7.31E-02
Ovarian cancer	H3K4me1	CD4+_CD25-_IL17+_PMA_Ionomycin_stim_Th17_primary	3.95E-02	2.04E-01	8.96E-02	5.17E+00	2.27E+00	8.13E-02	2.06E-08	2.39E-08	8.62E-01	1.94E-01
Ovarian cancer	H3K4me1	CD4+_CD25-_Th_primary	4.31E-02	2.37E-01	9.84E-02	5.50E+00	2.28E+00	6.31E-02	2.26E-08	2.28E-08	9.93E-01	1.60E-01
Ovarian cancer	H3K4me1	CD4+_CD25+_CD127-_Treg_primary	2.86E-02	1.59E-01	8.78E-02	5.55E+00	3.07E+00	1.59E-01	1.76E-08	2.95E-08	5.97E-01	2.75E-01
Ovarian cancer	H3K4me1	CD4+_CD25int_CD127+_Tmem_primary	1.59E-02	7.36E-02	6.46E-02	4.64E+00	4.07E+00	3.83E-01	7.26E-09	3.99E-08	1.82E-01	4.28E-01
Ovarian cancer	H3K4me1	CD56_primary	3.49E-02	8.57E-02	9.43E-02	2.46E+00	2.70E+00	5.94E-01	-1.25E-08	2.79E-08	-4.49E-01	6.73E-01
Ovarian cancer	H3K4me1	CD8_memory_primary	3.18E-02	1.64E-01	7.90E-02	5.16E+00	2.48E+00	1.05E-01	1.65E-08	2.50E-08	6.62E-01	2.54E-01
Ovarian cancer	H3K4me1	CD8_naive_primary_(BI)	3.19E-02	1.74E-01	8.34E-02	5.47E+00	2.62E+00	9.70E-02	2.15E-08	2.57E-08	8.35E-01	2.02E-01
Ovarian cancer	H3K4me1	CD8_naive_primary_(UCSF-UBC)	2.48E-02	8.41E-02	7.28E-02	3.39E+00	2.93E+00	4.20E-01	-2.02E-09	2.82E-08	-7.17E-02	5.29E-01
Ovarian cancer	H3K4me1	Cingulate_gyrus	4.10E-02	2.92E-02	1.12E-01	7.11E-01	2.74E+00	9.15E-01	-1.81E-08	2.60E-08	-6.96E-01	7.57E-01
Ovarian cancer	H3K4me1	Colon_smooth_muscle	3.52E-02	1.99E-01	1.07E-01	5.66E+00	3.03E+00	1.26E-01	3.18E-08	2.70E-08	1.18E+00	1.20E-01
Ovarian cancer	H3K4me1	Colonic_mucosa	2.17E-02	6.00E-02	1.04E-01	2.77E+00	4.81E+00	7.14E-01	-2.96E-09	4.63E-08	-6.38E-02	5.25E-01
Ovarian cancer	H3K4me1	Duodenum_Mucosa	4.83E-02	1.41E-01	1.23E-01	2.92E+00	2.55E+00	4.56E-01	-4.82E-09	3.05E-08	-1.58E-01	5.63E-01
Ovarian cancer	H3K4me1	Duodenum_smooth_muscle	6.00E-03	2.00E-02	8.26E-02	3.34E+00	1.38E+01	8.65E-01	-2.59E-09	1.21E-07	-2.14E-02	5.09E-01
Ovarian cancer	H3K4me1	Esophagus	5.79E-03	-1.13E-01	6.58E-02	-1.94E+01	1.14E+01	5.05E-02	-1.91E-07	8.95E-08	-2.13E+00	9.83E-01
Ovarian cancer	H3K4me1	Fetal_adrenal	4.34E-02	-1.58E-02	1.31E-01	-3.64E-01	3.02E+00	6.42E-01	-4.03E-08	3.03E-08	-1.33E+00	9.08E-01

Phenotypes	Mark	Category	Proportion of SNPs	Proportion of h^2	Proportion of h^2 SE	Enrichment	Enrichment SE	Enrichment *P*-value	Coefficient	Coefficient SE	Coefficient *Z*-value	Coefficient *P*-value
Ovarian cancer	H3K4me1	Fetal_brain	3.32E-02	4.39E-02	6.70E-02	1.33E+00	2.02E+00	8.72E-01	-9.07E-09	2.08E-08	-4.37E-01	6.69E-01
Ovarian cancer	H3K4me1	Fetal_heart	4.15E-02	6.37E-02	8.86E-02	1.54E+00	2.14E+00	8.03E-01	-1.20E-08	2.19E-08	-5.47E-01	7.08E-01
Ovarian cancer	H3K4me1	Fetal_large_intestine	3.04E-02	1.78E-01	1.06E-01	5.86E+00	3.49E+00	1.70E-01	2.74E-08	3.38E-08	8.12E-01	2.08E-01
Ovarian cancer	H3K4me1	Fetal_leg_muscle	4.09E-02	1.15E-02	1.20E-01	2.81E-01	2.93E+00	8.03E-01	-2.81E-08	2.79E-08	-1.01E+00	8.43E-01
Ovarian cancer	H3K4me1	Fetal_lung	6.99E-02	2.11E-01	1.43E-01	3.01E+00	2.04E+00	3.30E-01	6.05E-09	2.25E-08	2.69E-01	3.94E-01
Ovarian cancer	H3K4me1	Fetal_placenta	2.53E-02	-4.37E-02	1.02E-01	-1.73E+00	4.05E+00	4.83E-01	-5.10E-08	3.53E-08	-1.44E+00	9.26E-01
Ovarian cancer	H3K4me1	Fetal_small_intestine	3.38E-02	1.52E-01	9.97E-02	4.49E+00	2.95E+00	2.49E-01	1.44E-08	2.93E-08	4.90E-01	3.12E-01
Ovarian cancer	H3K4me1	Fetal_stomach	3.43E-02	-1.41E-02	1.28E-01	-4.10E-01	3.73E+00	6.93E-01	-3.39E-08	3.14E-08	-1.08E+00	8.60E-01
Ovarian cancer	H3K4me1	Fetal_thymus	3.62E-02	6.66E-02	9.70E-02	1.84E+00	2.68E+00	7.55E-01	-1.43E-08	2.49E-08	-5.73E-01	7.17E-01
Ovarian cancer	H3K4me1	Fetal_trunk_muscle	4.14E-02	5.09E-02	1.13E-01	1.23E+00	2.74E+00	9.33E-01	-1.46E-08	2.61E-08	-5.61E-01	7.13E-01
Ovarian cancer	H3K4me1	Gastric	1.36E-02	-3.41E-02	8.73E-02	-2.51E+00	6.42E+00	5.79E-01	-4.63E-08	5.96E-08	-7.77E-01	7.81E-01
Ovarian cancer	H3K4me1	Hippocampus_middle	5.18E-02	3.73E-02	1.26E-01	7.20E-01	2.43E+00	9.07E-01	-2.19E-08	2.55E-08	-8.57E-01	8.04E-01
Ovarian cancer	H3K4me1	Inferior_temporal_lobe	4.76E-02	5.76E-02	1.18E-01	1.21E+00	2.48E+00	9.32E-01	-1.40E-08	2.47E-08	-5.69E-01	7.15E-01
Ovarian cancer	H3K4me1	Kidney	8.50E-03	8.82E-02	6.66E-02	1.04E+01	7.84E+00	2.27E-01	7.18E-08	7.19E-08	9.98E-01	1.59E-01
Ovarian cancer	H3K4me1	Left_Ventricle	2.66E-02	-1.21E-02	9.66E-02	-4.56E-01	3.63E+00	6.88E-01	-2.66E-08	3.76E-08	-7.08E-01	7.61E-01
Ovarian cancer	H3K4me1	Liver_(BI)	5.50E-02	2.54E-01	1.13E-01	4.63E+00	2.06E+00	9.08E-02	1.56E-08	2.08E-08	7.54E-01	2.26E-01
Ovarian cancer	H3K4me1	Liver_(UCSD)	1.46E-03	2.56E-02	5.12E-02	1.76E+01	3.51E+01	6.27E-01	1.16E-07	2.77E-07	4.19E-01	3.37E-01
Ovarian cancer	H3K4me1	Lung	1.75E-02	-7.35E-02	8.65E-02	-4.19E+00	4.93E+00	3.01E-01	-7.34E-08	5.17E-08	-1.42E+00	9.22E-01
Ovarian cancer	H3K4me1	Mid_frontal_lobe	3.47E-03	4.58E-02	5.87E-02	1.32E+01	1.69E+01	4.66E-01	1.04E-07	1.46E-07	7.13E-01	2.38E-01
Ovarian cancer	H3K4me1	Mobilized_CD34_primary	6.92E-02	2.43E-01	1.44E-01	3.50E+00	2.08E+00	2.45E-01	2.36E-09	2.07E-08	1.14E-01	4.55E-01

**Phenotypes**	**Mark**	**Category**	**Proportion of SNPs**	**Proportion of *h*^{2}**	**Proportion of *h*^{2} SE**	**Enrichment**	**Enrichment SE**	**Enrichment *P*-value**	**Coefficient**	**Coefficient SE**	**Coefficient *Z*-value**	**Coefficient *P*-value**
Ovarian cancer	H3K4me1	Ovary	8.71E-03	1.10E-01	9.66E-02	1.26E+01	1.11E+01	2.79E-01	9.70E-08	9.30E-08	1.04E+00	1.48E-01
Ovarian cancer	H3K4me1	Pancreas	3.00E-02	2.96E-01	1.13E-01	9.86E+00	3.78E+00	1.69E-02	8.19E-08	3.55E-08	2.30E+00	1.06E-02
Ovarian cancer	H3K4me1	Pancreatic_islets	8.52E-03	7.13E-02	6.95E-02	8.37E+00	8.17E+00	3.45E-01	3.72E-08	6.37E-08	5.84E-01	2.80E-01
Ovarian cancer	H3K4me1	Pancreatic_islets	6.28E-03	5.41E-02	6.46E-02	8.61E+00	1.03E+01	4.42E-01	4.14E-08	8.58E-08	4.83E-01	3.15E-01
Ovarian cancer	H3K4me1	Penis_foreskin_fibroblast_primary	7.73E-02	1.23E-01	1.26E-01	1.59E+00	1.63E+00	7.20E-01	-2.76E-08	1.81E-08	-1.52E+00	9.36E-01
Ovarian cancer	H3K4me1	Penis_foreskin_keratinocyte_primary	6.09E-02	-4.19E-02	1.33E-01	-6.88E-01	2.18E+00	4.18E-01	-5.25E-08	2.17E-08	-2.42E+00	9.92E-01
Ovarian cancer	H3K4me1	Penis_foreskin_melanocyte_primary	6.53E-02	2.37E-01	1.36E-01	3.63E+00	2.08E+00	2.10E-01	8.67E-09	2.08E-08	4.17E-01	3.39E-01
Ovarian cancer	H3K4me1	Peripheralblood_mononuclear_primary	8.97E-03	4.65E-02	9.32E-02	5.19E+00	1.04E+01	6.88E-01	1.34E-08	8.99E-08	1.49E-01	4.41E-01
Ovarian cancer	H3K4me1	Placenta_amnion	4.05E-03	-1.58E-02	5.38E-02	-3.89E+00	1.33E+01	7.05E-01	-5.99E-08	1.08E-07	-5.55E-01	7.11E-01
Ovarian cancer	H3K4me1	Placenta_chorion	7.54E-03	-8.28E-02	6.01E-02	-1.10E+01	7.97E+00	1.20E-01	-1.22E-07	6.68E-08	-1.83E+00	9.67E-01
Ovarian cancer	H3K4me1	Psoas_muscle	3.93E-03	-2.66E-02	5.27E-02	-6.76E+00	1.34E+01	5.70E-01	-7.47E-08	1.18E-07	-6.34E-01	7.37E-01
Ovarian cancer	H3K4me1	Rectal_mucosa	3.50E-02	2.19E-01	1.30E-01	6.26E+00	3.71E+00	1.68E-01	3.54E-08	3.79E-08	9.32E-01	1.76E-01
Ovarian cancer	H3K4me1	Rectal_smooth_muscle	9.29E-03	5.60E-02	7.00E-02	6.03E+00	7.53E+00	5.06E-01	3.44E-08	6.50E-08	5.29E-01	2.98E-01
Ovarian cancer	H3K4me1	Right_atrium	9.22E-03	-1.40E-01	6.69E-02	-1.52E+01	7.25E+00	2.47E-02	-1.59E-07	6.81E-08	-2.33E+00	9.90E-01
Ovarian cancer	H3K4me1	Right_ventricle	8.10E-04	-4.38E-02	2.13E-02	-5.41E+01	2.62E+01	3.35E-02	-4.61E-07	2.20E-07	-2.09E+00	9.82E-01
Ovarian cancer	H3K4me1	Sigmoid_colon	3.01E-03	-1.25E-01	5.40E-02	-4.15E+01	1.79E+01	9.72E-03	-3.85E-07	1.48E-07	-2.61E+00	9.95E-01
Ovarian cancer	H3K4me1	Skeletal_muscle	6.68E-02	7.95E-02	1.29E-01	1.19E+00	1.93E+00	9.22E-01	-2.49E-08	1.80E-08	-1.38E+00	9.16E-01
Ovarian cancer	H3K4me1	Small_intestine	1.04E-03	-1.10E-02	3.76E-02	-1.06E+01	3.62E+01	7.48E-01	-1.13E-07	2.98E-07	-3.79E-01	6.48E-01
Ovarian cancer	H3K4me1	Spleen	4.59E-02	1.09E-01	1.25E-01	2.37E+00	2.72E+00	6.09E-01	-1.16E-08	2.94E-08	-3.94E-01	6.53E-01
Ovarian cancer	H3K4me1	Stomach_mucosa	3.36E-02	7.18E-02	1.01E-01	2.13E+00	3.00E+00	7.08E-01	-1.12E-08	3.10E-08	-3.60E-01	6.41E-01

Phenotypes	Mark	Category	Proportion of SNPs	Proportion of h^2	Proportion of h^2 SE	Enrichment	Enrichment SE	Enrichment *P*-value	Coefficient	Coefficient SE	Coefficient *Z*-value	Coefficient *P*-value
Ovarian cancer	H3K4me1	Stomach_smooth_muscle	2.39E-02	-5.45E-02	9.62E-02	-2.28E+00	4.02E+00	3.90E-01	-5.95E-08	3.49E-08	-1.70E+00	9.56E-01
Ovarian cancer	H3K4me1	Substantia_nigra	3.79E-02	5.31E-02	9.84E-02	1.40E+00	2.60E+00	8.77E-01	-1.07E-08	2.49E-08	-4.31E-01	6.67E-01
Ovarian cancer	H3K4me1	Thymus	8.80E-03	-7.35E-04	6.18E-02	-8.35E-02	7.02E+00	8.76E-01	-3.11E-08	6.12E-08	-5.09E-01	6.95E-01
Ovarian cancer	H3K4me3	Adipose_nuclei	2.93E-02	1.23E-01	1.27E-01	4.19E+00	4.36E+00	4.76E-01	1.66E-08	3.61E-08	4.59E-01	3.23E-01
Ovarian cancer	H3K4me3	Angular_gyrus	1.07E-02	-4.42E-02	1.05E-01	-4.11E+00	9.78E+00	5.92E-01	-1.18E-07	1.01E-07	-1.17E+00	8.78E-01
Ovarian cancer	H3K4me3	Anterior_caudate	1.92E-02	5.68E-02	1.24E-01	2.97E+00	6.48E+00	7.63E-01	-1.57E-08	7.72E-08	-2.04E-01	5.81E-01
Ovarian cancer	H3K4me3	Aorta	8.53E-03	5.21E-02	9.48E-02	6.10E+00	1.11E+01	6.49E-01	9.39E-09	1.00E-07	9.39E-02	4.63E-01
Ovarian cancer	H3K4me3	Breast_fibroblast_primary	5.80E-03	6.77E-02	1.04E-01	1.17E+01	1.80E+01	5.58E-01	6.14E-08	1.56E-07	3.94E-01	3.47E-01
Ovarian cancer	H3K4me3	Breast_myoepithelial	1.39E-02	-7.44E-02	1.12E-01	-5.35E+00	8.04E+00	4.03E-01	-1.46E-07	8.11E-08	-1.80E+00	9.64E-01
Ovarian cancer	H3K4me3	Breast_vHMEC	7.75E-03	-1.11E-04	9.45E-02	-1.43E-02	1.22E+01	9.32E-01	-8.10E-08	1.08E-07	-7.48E-01	7.73E-01
Ovarian cancer	H3K4me3	CD14_primary	8.37E-03	-2.44E-02	1.02E-01	-2.92E+00	1.22E+01	7.43E-01	-1.06E-07	1.14E-07	-9.25E-01	8.22E-01
Ovarian cancer	H3K4me3	CD15_primary	1.40E-02	4.11E-02	9.61E-02	2.93E+00	6.85E+00	7.79E-01	-2.46E-08	6.54E-08	-3.77E-01	6.47E-01
Ovarian cancer	H3K4me3	CD19_primary_(BI)	1.21E-02	1.79E-02	1.08E-01	1.48E+00	8.93E+00	9.57E-01	-5.95E-08	9.17E-08	-6.49E-01	7.42E-01
Ovarian cancer	H3K4me3	CD19_primary_(UW)	8.86E-03	-3.18E-03	1.06E-01	-3.59E-01	1.20E+01	9.08E-01	-7.99E-08	1.14E-07	-7.02E-01	7.59E-01
Ovarian cancer	H3K4me3	CD3_primary_(BI)	1.42E-02	1.23E-01	1.10E-01	8.70E+00	7.78E+00	3.39E-01	5.10E-08	7.18E-08	7.11E-01	2.39E-01
Ovarian cancer	H3K4me3	CD3_primary_(UW)	1.06E-02	1.97E-02	9.49E-02	1.85E+00	8.93E+00	9.23E-01	-4.50E-08	8.01E-08	-5.61E-01	7.13E-01
Ovarian cancer	H3K4me3	CD34_primary	1.07E-02	1.13E-01	1.16E-01	1.06E+01	1.09E+01	3.85E-01	6.41E-08	1.05E-07	6.13E-01	2.70E-01
Ovarian cancer	H3K4me3	CD4_memory_primary	1.17E-02	4.47E-02	9.67E-02	3.82E+00	8.26E+00	7.35E-01	-1.78E-08	7.35E-08	-2.42E-01	5.96E-01
Ovarian cancer	H3K4me3	CD4_naive_primary	1.27E-02	1.04E-01	9.59E-02	8.23E+00	7.57E+00	3.55E-01	4.59E-08	7.16E-08	6.41E-01	2.61E-01
Ovarian cancer	H3K4me3	CD4_primary	1.27E-02	3.99E-02	9.80E-02	3.15E+00	7.74E+00	7.82E-01	-2.91E-08	6.84E-08	-4.26E-01	6.65E-01

**Phenotypes**	**Mark**	**Category**	**Proportion of SNPs**	**Proportion of h^2**	**Proportion of h^2 SE**	**Enrichment**	**Enrichment SE**	**Enrichment *P*-value**	**Coefficient**	**Coefficient SE**	**Coefficient *Z*-value**	**Coefficient *P*-value**
Ovarian cancer	H3K4me3	CD4+_CD25-_CD45R0+_memory_primary	8.79E-03	5.35E-02	9.02E-02	6.09E+00	1.03E+01	6.24E-01	9.90E-09	8.62E-08	1.15E-01	4.54E-01
Ovarian cancer	H3K4me3	CD4+_CD25-_CD45RA+_naive_primary	1.31E-02	9.86E-02	1.04E-01	7.55E+00	7.95E+00	4.23E-01	3.60E-08	7.04E-08	5.12E-01	3.04E-01
Ovarian cancer	H3K4me3	CD4+_CD25-_IL17-_PMA_Ionomycin_stim_MACS_Th_sprimary	1.72E-02	1.10E-01	9.60E-02	6.40E+00	5.58E+00	3.51E-01	1.96E-08	4.85E-08	4.04E-01	3.43E-01
Ovarian cancer	H3K4me3	CD4+_CD25-_IL17+_PMA_Ionomycin_stim_Th17_primary	1.26E-02	1.05E-02	9.63E-02	8.33E-01	7.63E+00	9.82E-01	-5.91E-08	6.54E-08	-9.04E-01	8.17E-01
Ovarian cancer	H3K4me3	CD4+_CD25-_Th_primary	1.27E-02	8.13E-02	1.03E-01	6.40E+00	8.12E+00	5.17E-01	1.31E-08	7.57E-08	1.73E-01	4.31E-01
Ovarian cancer	H3K4me3	CD4+_CD25+_CD127-_Treg_primary	1.42E-02	6.55E-02	1.05E-01	4.62E+00	7.41E+00	6.30E-01	-1.03E-08	6.50E-08	-1.58E-01	5.63E-01
Ovarian cancer	H3K4me3	CD4+_CD25int_CD127+_Tmem_primary	1.08E-02	6.87E-02	1.04E-01	6.34E+00	9.60E+00	5.83E-01	1.77E-08	8.50E-08	2.08E-01	4.18E-01
Ovarian cancer	H3K4me3	CD56_primary	8.42E-03	-2.41E-02	1.01E-01	-2.87E+00	1.20E+01	7.37E-01	-1.09E-07	1.08E-07	-1.01E+00	8.45E-01
Ovarian cancer	H3K4me3	CD8_memory_primary	1.11E-02	6.00E-02	9.22E-02	5.39E+00	8.28E+00	6.01E-01	5.50E-09	7.86E-08	7.00E-02	4.72E-01
Ovarian cancer	H3K4me3	CD8_naive_primary_(BI)	1.14E-02	6.61E-02	9.75E-02	5.79E+00	8.54E+00	5.81E-01	1.21E-08	7.71E-08	1.56E-01	4.38E-01
Ovarian cancer	H3K4me3	CD8_naive_primary_(UCSF-UBC)	6.19E-03	-3.72E-02	9.00E-02	-6.02E+00	1.45E+01	6.16E-01	-1.39E-07	1.26E-07	-1.09E+00	8.63E-01
Ovarian cancer	H3K4me3	CD8_primary	1.04E-02	-5.79E-03	9.75E-02	-5.57E-01	9.38E+00	8.65E-01	-7.78E-08	8.24E-08	-9.44E-01	8.27E-01
Ovarian cancer	H3K4me3	Cingulate_gyrus	1.77E-02	1.24E-04	1.26E-01	7.03E-03	7.13E+00	8.88E-01	-5.64E-08	7.60E-08	-7.43E-01	7.71E-01
Ovarian cancer	H3K4me3	Colon_smooth_muscle	1.44E-02	2.78E-01	1.33E-01	1.93E+01	9.21E+00	6.62E-02	2.05E-07	9.16E-08	2.24E+00	1.25E-02
Ovarian cancer	H3K4me3	Colonic_mucosa	1.20E-02	1.99E-01	1.52E-01	1.66E+01	1.26E+01	2.46E-01	1.68E-07	1.33E-07	1.27E+00	1.02E-01
Ovarian cancer	H3K4me3	Duodenum_Mucosa	2.02E-02	3.07E-01	1.37E-01	1.52E+01	6.79E+00	5.36E-02	1.68E-07	7.12E-08	2.37E+00	8.98E-03
Ovarian cancer	H3K4me3	Duodenum_smooth_muscle	2.03E-02	1.71E-01	1.11E-01	8.42E+00	5.47E+00	2.01E-01	6.84E-08	4.80E-08	1.42E+00	7.73E-02
Ovarian cancer	H3K4me3	Esophagus	8.70E-03	-1.22E-02	1.08E-01	-1.40E+00	1.25E+01	8.44E-01	-8.58E-08	1.08E-07	-7.97E-01	7.87E-01
Ovarian cancer	H3K4me3	Fetal_adrenal	9.83E-03	9.74E-02	1.21E-01	9.91E+00	1.23E+01	4.79E-01	5.79E-08	1.22E-07	4.74E-01	3.18E-01
Ovarian cancer	H3K4me3	Fetal_brain	4.09E-03	-1.88E-02	7.64E-02	-4.61E+00	1.87E+01	7.60E-01	-1.07E-07	1.54E-07	-6.96E-01	7.57E-01

**Phenotypes**	**Mark**	**Category**	**Proportion of SNPs**	**Proportion of h^2**	**Proportion of h^2 SE**	**Enrichment**	**Enrichment SE**	**Enrichment *P*-value**	**Coefficient**	**Coefficient SE**	**Coefficient *Z*-value**	**Coefficient *P*-value**
Ovarian cancer	H3K4me3	Fetal_brain	1.29E-02	-4.53E-02	1.01E-01	-3.52E+00	7.82E+00	5.55E-01	-1.22E-07	8.19E-08	-1.49E+00	9.32E-01
Ovarian cancer	H3K4me3	Fetal_heart	5.82E-03	3.92E-02	7.82E-02	6.73E+00	1.34E+01	6.71E-01	1.46E-08	1.20E-07	1.22E-01	4.51E-01
Ovarian cancer	H3K4me3	Fetal_large_intestine	1.10E-02	2.24E-01	1.11E-01	2.05E+01	1.01E+01	7.84E-02	1.94E-07	1.02E-07	1.90E+00	2.86E-02
Ovarian cancer	H3K4me3	Fetal_leg_muscle	9.82E-03	1.40E-01	1.11E-01	1.43E+01	1.13E+01	2.56E-01	1.12E-07	1.10E-07	1.02E+00	1.54E-01
Ovarian cancer	H3K4me3	Fetal_lung	1.07E-02	1.84E-01	1.13E-01	1.71E+01	1.05E+01	1.42E-01	1.47E-07	1.03E-07	1.43E+00	7.61E-02
Ovarian cancer	H3K4me3	Fetal_placenta	8.25E-03	9.16E-02	9.25E-02	1.11E+01	1.12E+01	3.87E-01	6.73E-08	1.02E-07	6.59E-01	2.55E-01
Ovarian cancer	H3K4me3	Fetal_small_intestine	1.12E-02	2.50E-01	1.14E-01	2.23E+01	1.02E+01	5.79E-02	2.22E-07	1.02E-07	2.18E+00	1.47E-02
Ovarian cancer	H3K4me3	Fetal_stomach	1.02E-02	2.09E-01	1.24E-01	2.05E+01	1.21E+01	1.36E-01	1.96E-07	1.19E-07	1.65E+00	4.95E-02
Ovarian cancer	H3K4me3	Fetal_thymus	9.28E-03	3.89E-02	9.64E-02	4.20E+00	1.04E+01	7.60E-01	-2.00E-08	9.93E-08	-2.02E-01	5.80E-01
Ovarian cancer	H3K4me3	Fetal_trunk_muscle	8.87E-03	1.32E-01	1.07E-01	1.49E+01	1.21E+01	2.67E-01	1.18E-07	1.16E-07	1.02E+00	1.53E-01
Ovarian cancer	H3K4me3	Gastric	5.91E-03	-3.55E-02	9.50E-02	-6.00E+00	1.61E+01	6.57E-01	-1.28E-07	1.51E-07	-8.43E-01	8.00E-01
Ovarian cancer	H3K4me3	Germinal_matrix	1.16E-02	-5.98E-02	1.00E-01	-5.17E+00	8.67E+00	4.66E-01	-1.41E-07	9.15E-08	-1.54E+00	9.38E-01
Ovarian cancer	H3K4me3	Hippocampus_middle	1.89E-02	4.20E-02	1.28E-01	2.23E+00	6.80E+00	8.58E-01	-2.64E-08	7.48E-08	-3.52E-01	6.38E-01
Ovarian cancer	H3K4me3	Inferior_temporal_lobe	1.75E-02	1.33E-02	1.17E-01	7.60E-01	6.68E+00	9.71E-01	-4.58E-08	7.47E-08	-6.13E-01	7.30E-01
Ovarian cancer	H3K4me3	Kidney	1.74E-02	4.08E-01	1.73E-01	2.34E+01	9.93E+00	3.79E-02	2.49E-07	1.07E-07	2.32E+00	1.02E-02
Ovarian cancer	H3K4me3	Left_Ventricle	8.83E-03	2.53E-02	9.79E-02	2.86E+00	1.11E+01	8.67E-01	-3.21E-08	1.08E-07	-2.98E-01	6.17E-01
Ovarian cancer	H3K4me3	Liver_(BI)	1.63E-02	1.54E-01	9.80E-02	9.46E+00	6.03E+00	1.78E-01	6.58E-08	5.13E-08	1.28E+00	9.99E-02
Ovarian cancer	H3K4me3	Liver_(UCSD)	1.19E-02	1.30E-01	1.07E-01	1.10E+01	8.99E+00	2.87E-01	6.77E-08	8.68E-08	7.80E-01	2.18E-01
Ovarian cancer	H3K4me3	Lung	5.63E-03	3.27E-02	9.24E-02	5.81E+00	1.64E+01	7.70E-01	3.25E-09	1.55E-07	2.09E-02	4.92E-01
Ovarian cancer	H3K4me3	Mid_frontal_lobe	1.67E-02	-7.85E-03	1.20E-01	-4.71E-01	7.21E+00	8.37E-01	-6.98E-08	7.96E-08	-8.77E-01	8.10E-01

Phenotypes	Mark	Category	Proportion of SNPs	Proportion of h^2	Proportion of h^2 SE	Enrichment	Enrichment SE	Enrichment *P*-value	Coefficient	Coefficient SE	Coefficient *Z*-value	Coefficient *P*-value
Ovarian cancer	H3K4me3	Mobilized_CD34_primary	2.26E-02	1.12E-01	1.51E-01	4.97E+00	6.71E+00	5.60E-01	7.67E-09	6.55E-08	1.17E-01	4.53E-01
Ovarian cancer	H3K4me3	Ovary	9.60E-03	2.90E-01	1.46E-01	3.02E+01	1.52E+01	5.27E-02	2.96E-07	1.59E-07	1.86E+00	3.16E-02
Ovarian cancer	H3K4me3	Pancreas	8.35E-03	1.10E-01	1.01E-01	1.31E+01	1.21E+01	3.33E-01	1.00E-07	1.14E-07	8.81E-01	1.89E-01
Ovarian cancer	H3K4me3	Pancreatic_islets	1.01E-02	7.86E-02	9.20E-02	7.80E+00	9.13E+00	4.56E-01	2.18E-08	7.84E-08	2.78E-01	3.90E-01
Ovarian cancer	H3K4me3	Pancreatic_islets	1.14E-02	7.90E-02	1.11E-01	6.95E+00	9.75E+00	5.47E-01	1.56E-08	9.72E-08	1.61E-01	4.36E-01
Ovarian cancer	H3K4me3	Penis_foreskin_fibroblast_primary	3.74E-02	3.37E-02	1.13E-01	9.00E-01	3.01E+00	9.73E-01	-6.60E-08	3.01E-08	-2.19E+00	9.86E-01
Ovarian cancer	H3K4me3	Penis_foreskin_keratinocyte_primary	1.92E-02	-6.87E-03	1.21E-01	-3.58E-01	6.31E+00	8.27E-01	-8.22E-08	6.06E-08	-1.36E+00	9.12E-01
Ovarian cancer	H3K4me3	Penis_foreskin_melanocyte_primary	1.79E-02	1.96E-01	1.23E-01	1.10E+01	6.88E+00	1.66E-01	8.49E-08	6.63E-08	1.28E+00	1.00E-01
Ovarian cancer	H3K4me3	Peripheralblood_mononuclear_primary	9.72E-03	1.47E-02	1.05E-01	1.51E+00	1.08E+01	9.62E-01	-5.26E-08	9.70E-08	-5.43E-01	7.06E-01
Ovarian cancer	H3K4me3	Placenta_amnion	6.38E-03	6.03E-02	8.74E-02	9.46E+00	1.37E+01	5.43E-01	4.65E-08	1.16E-07	4.00E-01	3.45E-01
Ovarian cancer	H3K4me3	Placenta_chorion	8.67E-03	6.96E-03	8.26E-02	8.03E-01	9.53E+00	9.83E-01	-6.62E-08	8.82E-08	-7.51E-01	7.74E-01
Ovarian cancer	H3K4me3	Psoas_muscle	9.91E-03	9.73E-02	1.12E-01	9.82E+00	1.13E+01	4.47E-01	4.84E-08	1.11E-07	4.35E-01	3.32E-01
Ovarian cancer	H3K4me3	Rectal_mucosa	1.84E-02	2.86E-01	1.67E-01	1.56E+01	9.09E+00	1.31E-01	1.63E-07	9.62E-08	1.70E+00	4.48E-02
Ovarian cancer	H3K4me3	Rectal_smooth_muscle	1.30E-02	2.62E-01	1.52E-01	2.01E+01	1.16E+01	1.27E-01	2.21E-07	1.23E-07	1.80E+00	3.59E-02
Ovarian cancer	H3K4me3	Right_atrium	1.11E-02	1.18E-01	1.07E-01	1.06E+01	9.64E+00	3.39E-01	6.32E-08	9.79E-08	6.46E-01	2.59E-01
Ovarian cancer	H3K4me3	Right_ventricle	1.08E-02	4.81E-02	1.14E-01	4.44E+00	1.05E+01	7.45E-01	-1.44E-08	9.87E-08	-1.45E-01	5.58E-01
Ovarian cancer	H3K4me3	Sigmoid_colon	5.57E-03	1.88E-01	1.04E-01	3.38E+01	1.87E+01	1.01E-01	3.18E-07	1.70E-07	1.87E+00	3.05E-02
Ovarian cancer	H3K4me3	Skeletal_muscle	2.21E-02	1.60E-01	1.35E-01	7.25E+00	6.10E+00	3.23E-01	5.13E-08	5.99E-08	8.57E-01	1.96E-01
Ovarian cancer	H3K4me3	Small_intestine	4.95E-03	1.41E-01	1.06E-01	2.85E+01	2.15E+01	2.22E-01	2.50E-07	1.91E-07	1.31E+00	9.53E-02
Ovarian cancer	H3K4me3	Spleen	4.17E-03	1.24E-02	8.85E-02	2.97E+00	2.12E+01	9.26E-01	-2.11E-08	1.90E-07	-1.11E-01	5.44E-01

**Phenotypes**	**Mark**	**Category**	**Proportion of SNPs**	**Proportion of h^2**	**Proportion of h^2 SE**	**Enrichment**	**Enrichment SE**	**Enrichment *P*-value**	**Coefficient**	**Coefficient SE**	**Coefficient *Z*-value**	**Coefficient *P*-value**
Ovarian cancer	H3K4me3	Stomach_mucosa	5.91E-03	6.22E-03	9.63E-02	1.05E+00	1.63E+01	9.97E-01	-4.88E-08	1.42E-07	-3.43E-01	6.34E-01
Ovarian cancer	H3K4me3	Stomach_smooth_muscle	2.01E-02	7.88E-02	1.37E-01	3.92E+00	6.82E+00	6.71E-01	-1.16E-10	6.18E-08	-1.88E-03	5.01E-01
Ovarian cancer	H3K4me3	Substantia_nigra	1.44E-02	8.30E-02	1.19E-01	5.77E+00	8.24E+00	5.69E-01	2.32E-08	8.61E-08	2.69E-01	3.94E-01
Ovarian cancer	H3K4me3	Treg_primary	1.78E-02	1.06E-01	8.00E-02	5.94E+00	4.50E+00	2.90E-01	1.45E-08	3.68E-08	3.95E-01	3.47E-01
Ovarian cancer	H3K9ac	Adipose_nuclei	2.86E-02	-1.01E-01	1.36E-01	-3.53E+00	4.75E+00	2.92E-01	-9.71E-08	3.84E-08	-2.53E+00	9.94E-01
Ovarian cancer	H3K9ac	Angular_gyrus	1.10E-02	-1.58E-03	1.01E-01	-1.44E-01	9.23E+00	9.00E-01	-3.28E-08	8.61E-08	-3.81E-01	6.48E-01
Ovarian cancer	H3K9ac	Anterior_caudate	1.46E-02	5.98E-02	1.13E-01	4.09E+00	7.76E+00	6.93E-01	1.46E-08	7.80E-08	1.87E-01	4.26E-01
Ovarian cancer	H3K9ac	Breast_myoepithelial	4.64E-03	-3.79E-02	9.42E-02	-8.17E+00	2.03E+01	6.47E-01	-1.12E-07	1.73E-07	-6.47E-01	7.41E-01
Ovarian cancer	H3K9ac	CD8_naive_primary_(UCSF-UBC)	2.18E-03	-3.50E-02	5.77E-02	-1.60E+01	2.64E+01	4.92E-01	-1.89E-07	1.94E-07	-9.75E-01	8.35E-01
Ovarian cancer	H3K9ac	Cingulate_gyrus	1.92E-02	2.79E-03	1.14E-01	1.46E-01	5.92E+00	8.83E-01	-2.60E-08	5.97E-08	-4.35E-01	6.68E-01
Ovarian cancer	H3K9ac	Colon_smooth_muscle	5.67E-03	9.17E-02	9.14E-02	1.62E+01	1.61E+01	3.62E-01	1.13E-07	1.31E-07	8.62E-01	1.94E-01
Ovarian cancer	H3K9ac	Colonic_mucosa	1.81E-02	1.90E-01	1.25E-01	1.05E+01	6.86E+00	1.93E-01	9.61E-08	6.53E-08	1.47E+00	7.05E-02
Ovarian cancer	H3K9ac	Duodenum_Mucosa	1.51E-02	1.19E-01	1.21E-01	7.86E+00	8.00E+00	4.05E-01	5.06E-08	8.13E-08	6.22E-01	2.67E-01
Ovarian cancer	H3K9ac	Fetal_brain	1.27E-02	-4.83E-02	1.09E-01	-3.81E+00	8.60E+00	5.61E-01	-9.09E-08	7.89E-08	-1.15E+00	8.75E-01
Ovarian cancer	H3K9ac	Fetal_heart	2.05E-02	5.21E-02	1.01E-01	2.54E+00	4.94E+00	7.57E-01	-1.30E-08	4.44E-08	-2.92E-01	6.15E-01
Ovarian cancer	H3K9ac	Fetal_kidney	5.04E-03	2.28E-01	1.49E-01	4.53E+01	2.96E+01	1.50E-01	4.14E-07	2.75E-07	1.51E+00	6.60E-02
Ovarian cancer	H3K9ac	Fetal_lung	1.30E-02	8.84E-02	1.31E-01	6.80E+00	1.01E+01	5.64E-01	3.74E-08	9.34E-08	4.01E-01	3.44E-01
Ovarian cancer	H3K9ac	Hippocampus_middle	1.79E-02	-2.28E-02	1.14E-01	-1.27E+00	6.38E+00	7.13E-01	-4.83E-08	5.82E-08	-8.31E-01	7.97E-01
Ovarian cancer	H3K9ac	Inferior_temporal_lobe	1.68E-02	2.72E-02	1.13E-01	1.62E+00	6.72E+00	9.27E-01	-7.25E-09	6.33E-08	-1.15E-01	5.46E-01
Ovarian cancer	H3K9ac	Kidney	1.08E-02	2.75E-01	1.66E-01	2.56E+01	1.54E+01	1.32E-01	2.56E-07	1.57E-07	1.63E+00	5.12E-02

**Phenotypes**	**Mark**	**Category**	**Proportion of SNPs**	**Proportion of h^2**	**Proportion of h^2 SE**	**Enrichment**	**Enrichment SE**	**Enrichment *P*-value**	**Coefficient**	**Coefficient SE**	**Coefficient *Z*-value**	**Coefficient *P*-value**
Ovarian cancer	H3K9ac	Liver_(BI)	1.41E-02	1.88E-01	9.82E-02	1.34E+01	6.97E+00	8.61E-02	9.66E-08	5.92E-08	1.63E+00	5.13E-02
Ovarian cancer	H3K9ac	Mid_frontal_lobe	1.51E-02	3.52E-03	1.11E-01	2.33E-01	7.36E+00	9.16E-01	-2.80E-08	7.67E-08	-3.65E-01	6.42E-01
Ovarian cancer	H3K9ac	Pancreatic_islets	6.24E-03	-3.38E-02	9.57E-02	-5.42E+00	1.53E+01	6.72E-01	-1.18E-07	1.44E-07	-8.18E-01	7.93E-01
Ovarian cancer	H3K9ac	Penis_foreskin_keratinocyte_primary	3.27E-02	-6.30E-02	1.30E-01	-1.93E+00	3.99E+00	4.38E-01	-5.72E-08	3.97E-08	-1.44E+00	9.25E-01
Ovarian cancer	H3K9ac	Peripheralblood_mononuclear_primary	1.32E-02	-3.52E-02	1.07E-01	-2.66E+00	8.10E+00	6.37E-01	-8.55E-08	7.31E-08	-1.17E+00	8.79E-01
Ovarian cancer	H3K9ac	Rectal_mucosa	1.61E-02	2.24E-01	1.30E-01	1.39E+01	8.03E+00	1.29E-01	1.37E-07	7.56E-08	1.82E+00	3.45E-02
Ovarian cancer	H3K9ac	Rectal_smooth_muscle	3.57E-03	1.95E-02	7.46E-02	5.46E+00	2.09E+01	8.31E-01	4.24E-09	1.73E-07	2.46E-02	4.90E-01
Ovarian cancer	H3K9ac	Skeletal_muscle	2.76E-02	2.91E-02	1.20E-01	1.05E+00	4.34E+00	9.90E-01	-2.67E-08	3.45E-08	-7.74E-01	7.80E-01
Ovarian cancer	H3K9ac	Stomach_mucosa	1.31E-02	1.50E-01	1.20E-01	1.15E+01	9.17E+00	2.70E-01	9.14E-08	8.74E-08	1.05E+00	1.48E-01
Ovarian cancer	H3K9ac	Stomach_smooth_muscle	1.40E-02	-2.07E-02	1.15E-01	-1.48E+00	8.23E+00	7.54E-01	-6.81E-08	6.89E-08	-9.90E-01	8.39E-01
Ovarian cancer	H3K9ac	Substantia_nigra	1.69E-02	4.00E-02	1.07E-01	2.37E+00	6.35E+00	8.30E-01	4.06E-09	5.79E-08	7.01E-02	4.72E-01
