# Supplementary material for: Investigating the shared genetic architecture between breast and ovarian cancers
Source: Genet Mol Biol. 2024 Apr 15;47(2):e20230181. doi: 10.1590/1678-4685-GMB-2023-0181 (PMC11021043; doi:10.1590/1678-4685-GMB-2023-0181)
Supplement: Table S6 - [file 1415-4757-GMB-47-02-e20230181-s6.pdf]

Genetics and Molecular Biology

https://doi.org/10.1590/1678-4685-GMB-2023-0181

Supplementary Material to "Investigating the shared genetic architecture between breast and ovarian cancers"

Table S6 - The genes identified by the MTaSPUsSet and aSPUs test.

Chromosome	Gene	MTaSPUsSet *P*-value	aSPUs *P*-value	
			Breast cancer	Ovarian cancer
2	*ACOXL*	**9.99E-07**	**3.00E-06**	2.70E-04
4	*ADAM29*	**9.99E-07**	**9.99E-07**	2.00E-04
2	*ALS2CR12*	**9.99E-07**	**9.99E-07**	7.75E-03
8	*ANXA13*	**9.99E-07**	**9.99E-07**	6.05E-01
9	*ASTN2*	**9.99E-07**	**9.99E-07**	8.57E-01
10	*ATE1*	**9.99E-07**	**9.99E-07**	9.20E-01
6	*ATXN1*	**9.99E-07**	**9.99E-07**	4.53E-01
9	*BNC2*	**9.99E-07**	8.93E-03	**9.99E-07**
6	*BTN2A1*	**9.99E-07**	**9.99E-07**	9.23E-01
5	*C5orf56*	**9.99E-07**	**9.99E-07**	4.58E-01
16	*CASC16*	**9.99E-07**	**9.99E-07**	9.83E-01
8	*CASC21*	**9.99E-07**	**9.99E-07**	4.84E-01
8	*CASC8*	**9.99E-07**	**9.99E-07**	7.29E-01
8	*CASC9*	**9.99E-07**	**9.99E-07**	7.26E-01
6	*CCDC170*	**9.99E-07**	**9.99E-07**	7.16E-03
14	*CCDC88C*	**9.99E-07**	**9.99E-07**	3.01E-01
12	*CCDC91*	**9.99E-07**	**9.99E-07**	7.59E-01
9	*CDKN2A*	**9.99E-07**	**9.99E-07**	1.67E-01
16	*CDYL2*	**9.99E-07**	**9.99E-07**	8.70E-01
8	*CHMP4C*	**9.99E-07**	1.20E-02	**9.99E-07**
18	*CHST9*	**9.99E-07**	**9.99E-07**	5.70E-04
3	*CMSS1*	**9.99E-07**	**9.99E-07**	4.68E-01
3	*COL8A1*	**9.99E-07**	**9.99E-07**	4.93E-03
17	*CRHR1*	**9.99E-07**	1.00E-04	**9.99E-07**
2	*DIRC3*	**9.99E-07**	**9.99E-07**	5.06E-01
5	*EBF1*	**9.99E-07**	**9.99E-07**	3.40E-02

Chromosome	Gene	MTaSPUsSet *P*-value	aSPUs *P*-value	
			Breast cancer	Ovarian cancer
2	*EFR3B*	**9.99E-07**	**9.99E-07**	2.04E-02
6	*ESR1*	**9.99E-07**	**9.99E-07**	2.37E-02
5	*FGF10*	**9.99E-07**	**9.99E-07**	4.82E-01
10	*FGFR2*	**9.99E-07**	**9.99E-07**	5.12E-01
3	*FILIP1L*	**9.99E-07**	**9.99E-07**	6.00E-04
19	*FKBP8*	**9.99E-07**	**9.99E-07**	5.09E-01
16	*FTO*	**9.99E-07**	**9.99E-07**	5.34E-01
19	*GATAD2A*	**9.99E-07**	**9.99E-07**	4.46E-02
19	*GIPR*	**9.99E-07**	**9.99E-07**	2.91E-01
5	*HCN1*	**9.99E-07**	**9.99E-07**	5.79E-01
2	*HOXD3*	**9.99E-07**	6.32E-01	**9.99E-07**
3	*ITPR1*	**9.99E-07**	**9.99E-07**	9.64E-01
8	*KCNU1*	**9.99E-07**	**9.99E-07**	5.08E-01
9	*LMX1B*	**9.99E-07**	**9.99E-07**	4.01E-01
9	*LOC100128505*	**9.99E-07**	**9.99E-07**	5.61E-01
11	*LSP1*	**9.99E-07**	**9.99E-07**	4.26E-01
19	*LYPD5*	**9.99E-07**	**9.99E-07**	8.41E-01
3	*MIR548G*	**9.99E-07**	**9.99E-07**	8.30E-04
22	*MKL1*	**9.99E-07**	**9.99E-07**	6.77E-01
10	*MLLT10*	**9.99E-07**	**9.99E-07**	1.00E-05
3	*MYL3*	**9.99E-07**	**9.99E-07**	5.54E-01
1	*NBPF10*	**9.99E-07**	**9.99E-07**	6.97E-01
1	*NBPF20*	**9.99E-07**	**9.99E-07**	7.21E-01
21	*NRIP1*	**9.99E-07**	**9.99E-07**	2.31E-03
17	*NSF*	**9.99E-07**	7.60E-04	**9.99E-07**
5	*PDE4D*	**9.99E-07**	**9.99E-07**	5.14E-02
1	*PEX14*	**9.99E-07**	**9.99E-07**	9.95E-01
10	*PIP4K2A*	**9.99E-07**	**9.99E-07**	2.27E-01
9	*PRRX2*	**9.99E-07**	**9.99E-07**	5.44E-01
14	*RAD51B*	**9.99E-07**	**9.99E-07**	9.25E-01
1	*RUSC1*	**9.99E-07**	**9.99E-07**	7.77E-02
14	*SLC25A21*	**9.99E-07**	**9.99E-07**	6.03E-01
3	*SLC4A7*	**9.99E-07**	**9.99E-07**	9.47E-02
17	*STXBP4*	**9.99E-07**	**9.99E-07**	3.72E-01
6	*SYNE1*	**9.99E-07**	**9.99E-07**	6.44E-02
10	*TCF7L2*	**9.99E-07**	**9.99E-07**	2.77E-01
5	*TERT*	**9.99E-07**	**9.99E-07**	**9.99E-07**
3	*TGFBR2*	**9.99E-07**	**9.99E-07**	7.53E-01
11	*TNNT3*	**9.99E-07**	**9.99E-07**	1.91E-01
2	*TNS1*	**9.99E-07**	**9.99E-07**	8.03E-01
16	*TOX3*	**9.99E-07**	**9.99E-07**	9.52E-01

Chromosome	Gene	MTaSPUsSet *P*-value	aSPUs *P*-value	
			Breast cancer	Ovarian cancer
22	*TTC28*	**9.99E-07**	**9.99E-07**	1.03E-03
5	*WWC1*	**9.99E-07**	2.99E-01	**9.99E-07**
3	*ZBTB38*	**9.99E-07**	**9.99E-07**	7.92E-01
8	*ZFPM2*	**9.99E-07**	**9.99E-07**	7.06E-01
10	*ZMIZ1*	**9.99E-07**	**9.99E-07**	6.85E-01
10	*ZNF365*	**9.99E-07**	**9.99E-07**	1.52E-02
19	*ZNF45*	**9.99E-07**	**9.99E-07**	9.03E-01
