# Supplementary material for: Investigating the shared genetic architecture between breast and ovarian cancers
Source: Genet Mol Biol. 2024 Apr 15;47(2):e20230181. doi: 10.1590/1678-4685-GMB-2023-0181 (PMC11021043; doi:10.1590/1678-4685-GMB-2023-0181)
Supplement: Table S7 - [file 1415-4757-GMB-47-02-e20230181-s7.pdf]

Genetics and Molecular Biology

https://doi.org/10.1590/1678-4685-GMB-2023-0181

Supplementary Material to "Investigating the shared genetic architecture between breast and ovarian cancers"

Table S7 - The significant pathway enrichment of the pleiotropic genes.

GeneSet	N	n	*P*-value	adjusted P	genes
**GO biological processes**					
GO_ORGAN_GROWTH	196	7	1.48E-07	2.73E-04	FGFR2 ,TGFBR2 ,FGF10 ,WWC1 ,ESR1 ,ZFPM2 ,BNC2
GO_MAMMARY_GLAND_MORPHOGENESIS	46	4	2.34E-06	2.15E-03	FGFR2 ,TGFBR2 ,FGF10 ,ESR1
GO_BRANCH_ELONGATION_OF_AN_EPITHELIUM	20	3	8.79E-06	5.87E-03	FGFR2 ,FGF10 ,ESR1
GO_MAMMARY_GLAND_DUCT_MORPHOGENESIS	33	3	4.13E-05	1.38E-02	FGFR2 ,FGF10 ,ESR1
GO_REGULATION_OF_ORGAN_GROWTH	109	4	7.25E-05	1.67E-02	FGFR2 ,TGFBR2 ,WWC1 ,ZFPM2
GO_MAMMARY_GLAND_FORMATION	7	2	8.40E-05	1.67E-02	FGFR2 ,FGF10
GO_FEMALE_SEX_DIFFERENTIATION	116	4	9.23E-05	1.79E-02	NRIP1 ,FGF10 ,ESR1 ,ZFPM2
GO_POSITIVE_REGULATION_OF_CELL_POPULATION_PROLIFERATION	965	9	1.39E-04	2.13E-02	ZMIZ1 ,TCF7L2 ,FGFR2 ,STXBP4 ,TGFBR2 ,TERT ,FGF10 ,ESR1 ,ZFPM2
GO_MAMMARY_GLAND_DEVELOPMENT	140	4	1.91E-04	2.26E-02	FGFR2 ,TGFBR2 ,FGF10 ,ESR1

GO_EPITHELIAL_CELL_PROLIFERATION	415	6	1.96E-04	2.26E-02	TCF7L2 ,FGFR2 ,STXBP4 ,COL8A1 ,FGF10 ,ESR1
GO_POSITIVE_REGULATION_OF_CELL_CYCLE_G1_S_PHASE_TRANSITION	60	3	2.49E-04	2.50E-02	STXBP4 ,TERT ,FGF10
GO_REPLICATIVE_SENESCENCE	14	2	3.61E-04	3.06E-02	TERT ,CDKN2A
GO_REGULATION_OF_CELL_CYCLE_PHASE_TRANSITION	468	6	3.73E-04	3.06E-02	RAD51B ,STXBP4 ,TERT ,FGF10 ,CHMP4C ,CDKN2A
GO_MAMMARY_GLAND_EPITHELIUM_DEVELOPMENT	71	3	4.09E-04	3.10E-02	FGFR2 ,FGF10 ,ESR1
GO_REGULATION_OF_CELL_POPULATION_PROLIFERATION	1684	11	5.36E-04	3.42E-02	ZMIZ1 ,TCF7L2 ,FGFR2 ,FTO ,STXBP4 ,TGFBR2 ,TERT ,FGF10 ,ESR1 ,ZFPM2 ,CDKN2A
GO_FEMALE_GENITALIA_DEVELOPMENT	17	2	5.37E-04	3.42E-02	FGF10 ,ESR1
GO_REGULATION_OF_CELL_CYCLE_G1_S_PHASE_TRANSITION	193	4	6.43E-04	3.81E-02	STXBP4 ,TERT ,FGF10 ,CDKN2A
GO_REGULATION_OF_CELL_CYCLE	1201	9	6.86E-04	3.94E-02	TCF7L2 ,FGFR2 ,RAD51B ,STXBP4 ,TTC28 ,TERT ,FGF10 ,CHMP4C ,CDKN2A
GO_POSITIVE_REGULATION_OF_EPITHELIAL_CELL_PROLIFERATION	202	4	7.62E-04	4.21E-02	TCF7L2 ,FGFR2 ,STXBP4 ,FGF10
GO_NEGATIVE_REGULATION_OF_CELL_DEATH	1003	8	9.30E-04	4.71E-02	TCF7L2 ,FGFR2 ,TOX3 ,CRHR1 ,FKBP8 ,TERT ,FGF10 ,ZFPM2
**GO molecular functions**					
GO_DNA_BINDING_TRANSCRIPTION_FACTOR_ACTIVITY	1691	13	3.02E-05	2.49E-02	MLLT10 ,TCF7L2 ,TOX3 ,GATAD2A ,ZNF45 ,HOXD3 ,ZBTB38 ,EBF1 ,ESR1 ,ZFPM2 ,BNC2 ,LMX1B ,PRRX2
GO_SEQUENCE_SPECIFIC_DNA_BINDING	1114	10	7.87E-05	4.31E-02	TCF7L2 ,TOX3 ,GATAD2A ,HOXD3 ,NRIP1 ,ZBTB38 ,TERT ,ESR1 ,LMX1B ,PRRX2
GO_CYTOSKELETAL_PROTEIN_BINDING	948	9	1.21E-04	4.99E-02	PEX14 ,RUSC1 ,LSP1 ,TNNT3 ,LSP1 ,CCDC88C ,TNS1 ,MKL1 ,MYL3 ,SYNE1
**Cancer gene neighborhoods**					
MORF_ARL3	304	6	3.56E-05	1.52E-02	MLLT10 ,CRHR1 ,MYL3 ,COL8A1 ,PDE4D ,ESR1
MORF_FOSL1	407	6	1.77E-04	3.77E-02	MLLT10 ,CRHR1 ,MYL3 ,COL8A1 ,PDE4D ,ESR1
MORF_NOS2A	288	5	2.99E-04	3.85E-02	MLLT10 ,CRHR1 ,COL8A1 ,PDE4D ,ESR1
MORF_ERCC4	320	5	4.83E-04	3.85E-02	MLLT10 ,CRHR1 ,MYL3 ,COL8A1 ,PDE4D
MORF_PRKCA	181	4	5.05E-04	3.85E-02	MLLT10 ,CRHR1 ,COL8A1 ,PDE4D
MORF_BCL2L11	185	4	5.49E-04	3.85E-02	MLLT10 ,CRHR1 ,COL8A1 ,PDE4D
MORF_IL4	192	4	6.31E-04	3.85E-02	MLLT10 ,CRHR1 ,COL8A1 ,PDE4D

MORF_IFNA1	204	4	7.91E-04	4.22E-02	MLLT10 ,CRHR1 ,COL8A1 ,PDE4D
MORF_KDR	94	3	9.29E-04	4.41E-02	MLLT10 ,MYL3 ,COL8A1
MORF_TFDP2	230	4	1.23E-03	4.95E-02	MLLT10 ,CRHR1 ,PDE4D ,ESR1
MORF_IL16	239	4	1.42E-03	4.95E-02	MLLT10 ,CRHR1 ,COL8A1 ,ESR1
MORF_MAGEA9	422	5	1.66E-03	4.95E-02	MLLT10 ,CRHR1 ,COL8A1 ,PDE4D ,ESR1
MORF_CASP10	117	3	1.74E-03	4.95E-02	MLLT10 ,CRHR1 ,ESR1
MORF_PTPRB	253	4	1.75E-03	4.95E-02	MLLT10 ,MYL3 ,COL8A1 ,PDE4D
MORF_MAGEA8	259	4	1.90E-03	4.95E-02	MLLT10 ,CRHR1 ,COL8A1 ,PDE4D
MORF_BRCA1	264	4	2.04E-03	4.95E-02	MLLT10 ,CRHR1 ,PDE4D ,ESR1
MORF_PAX7	267	4	2.12E-03	4.95E-02	MLLT10 ,CRHR1 ,PDE4D ,ESR1
MORF_CD8A	126	3	2.15E-03	4.95E-02	MLLT10 ,COL8A1 ,PDE4D
MORF_THPO	127	3	2.20E-03	4.95E-02	MLLT10 ,CRHR1 ,COL8A1
**Cancer_modules**					
MODULE_202	28	3	2.50E-05	1.08E-02	LSP1 ,TNNT3 ,LSP1 ,MYL3
