# Supplementary material for: Investigating the shared genetic architecture between breast and ovarian cancers
Source: Genet Mol Biol. 2024 Apr 15;47(2):e20230181. doi: 10.1590/1678-4685-GMB-2023-0181 (PMC11021043; doi:10.1590/1678-4685-GMB-2023-0181)
Supplement: Figure S1 - [file 1415-4757-GMB-47-02-e20230181-s8.pdf]

Genetics and Molecular Biology
https://doi.org/10.1590/1678-4685-GMB-2023-0181

Supplementary Material to "Investigating the shared genetic architecture between breast and ovarian cancers"

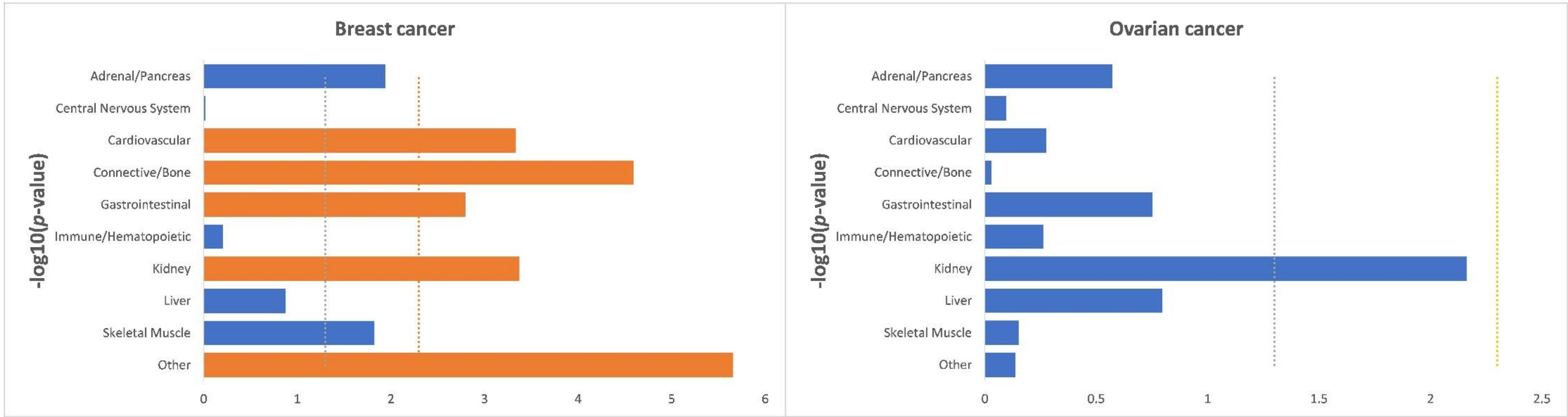

Figure S1 - Cell-type-group-specific enrichment of SNP heritability for cancers. The x-axis represented each of the 10 cell type groups, y-axis represented the log-transformed P-values of coefficient Z scores. Statistically significant annotations after Bonferroni corrections (P<0.05/10) were plotted in orange, otherwise in blue. The horizontal grey dash line indicated P-threshold of 0.05; horizontal red dash line indicated P-threshold of 0.05/10.
