# Supplementary material for: Investigating the shared genetic architecture between breast and ovarian cancers
Source: Genet Mol Biol. 2024 Apr 15;47(2):e20230181. doi: 10.1590/1678-4685-GMB-2023-0181 (PMC11021043; doi:10.1590/1678-4685-GMB-2023-0181)
Supplement: Figure S2 - [file 1415-4757-GMB-47-02-e20230181-s9.pdf]

Genetics and Molecular Biology
https://doi.org/10.1590/1678-4685-GMB-2023-0181

Supplementary Material to "Investigating the shared genetic architecture between breast and ovarian cancers"

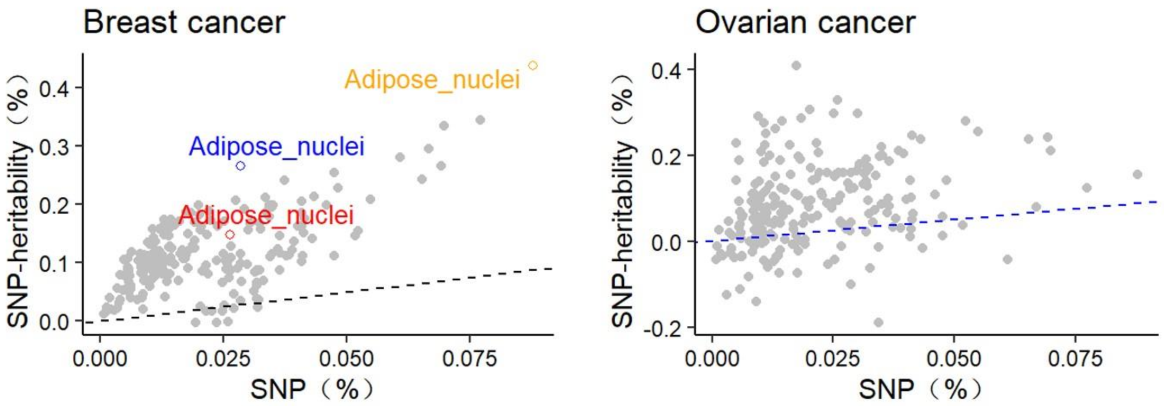

Figure S2 - Cell-type-specific enrichment of SNP heritability for cancers. The x-axis represented the proportion of SNPs in annotations, y-axis represented the proportion of heritability explained by that annotation. Statistically significant annotations after Bonferroni corrections ($P<0.05/220$) were plotted in color (H3K4me1, H3K4me3, H3K9ac and H3K27ac were marked in orange, blue, red and green, respectively), the remaining annotations were marked in grey.
