# Supplementary material for: Investigating the shared genetic architecture between breast and ovarian cancers
Source: Genet Mol Biol. 2024 Apr 15;47(2):e20230181. doi: 10.1590/1678-4685-GMB-2023-0181 (PMC11021043; doi:10.1590/1678-4685-GMB-2023-0181)
Supplement: Figure S3 - [file 1415-4757-GMB-47-02-e20230181-s10.pdf]

Genetics and Molecular Biology
https://doi.org/10.1590/1678-4685-GMB-2023-0181

Supplementary Material to “Investigating the shared genetic architecture between breast and ovarian cancers”

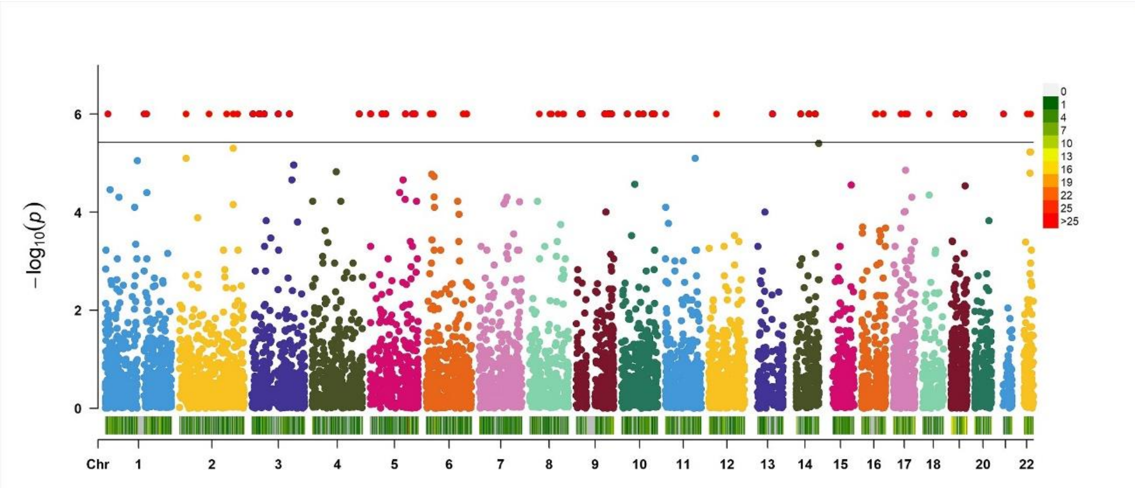

Figure S3 - Manhattan plot with –log10(*P*) values of MTaSPUsSet test for single gene with two cancers analysis. The black line is a threshold with the –log10(*P*) value of 5.40 corresponding to P < 3.75×10-6. If the –log10(P) value of a certain gene was >5.40, this gene was identified as significant for breast cancer and ovarian cancer. Gene density plots indicate the distribution of genes on chromosomes.
